# Supplementary material for: Edge-strand of BepA interacts with immature LptD on the β-barrel assembly machine to direct it to on- and off-pathways
Source: eLife. 2021 Aug 31;10:e70541. doi: 10.7554/eLife.70541 (PMC8423444; doi:10.7554/eLife.70541)
Supplement: Figure 1—source data 1. — (B–D) For the immunoblotting experiments using the anti-BepA and anti-LptD antibodies and quantified band intensity data for the pulse-chase experiments using the anti-LptD antibody. [file elife-70541-fig1-data1.zip › Figure 1 Source data files(revised)/Figure 1-Source data 1 (B-D,_used area & Source for quantitation).pdf]

Figure 1-Source Data

Figure 1B

αLptD Original image Used area

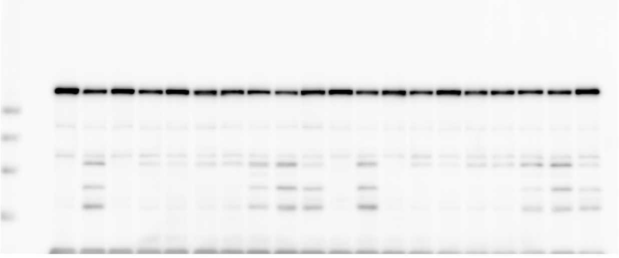

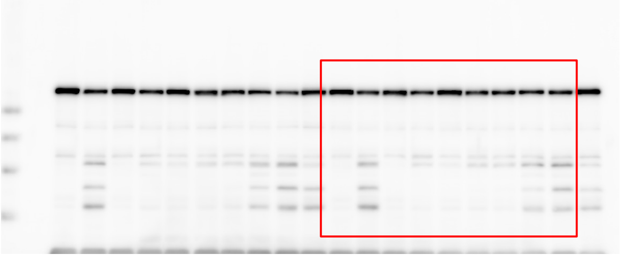

αBepA Original image Used area

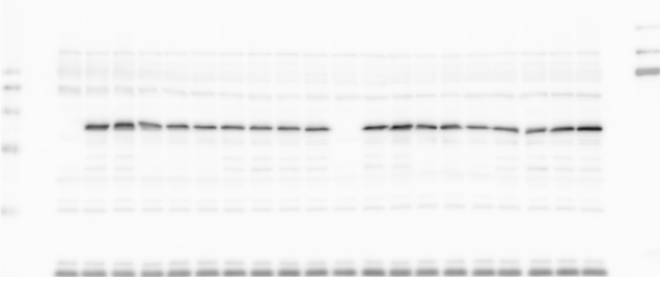

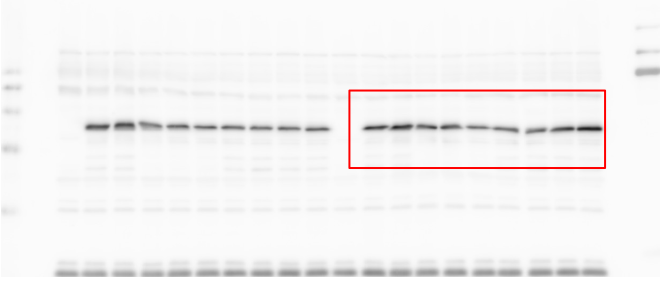

Figure 1C

αLptD Original image Used area

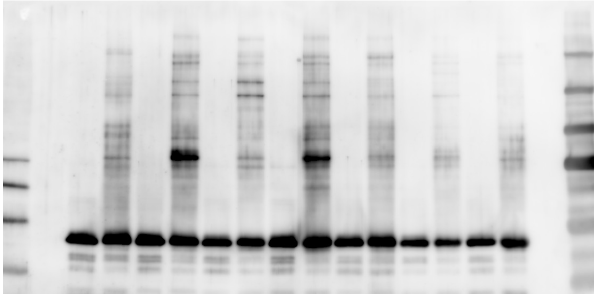

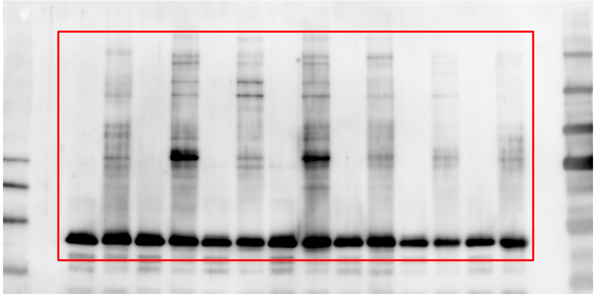

αBepA Original image Used area

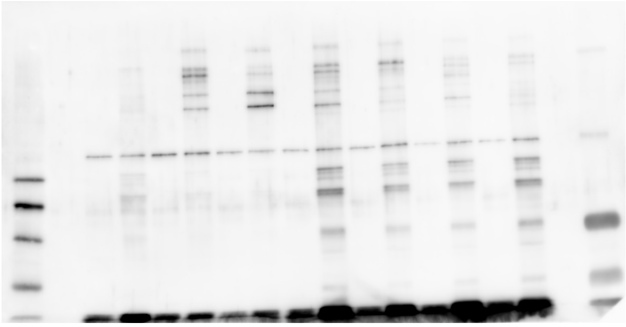

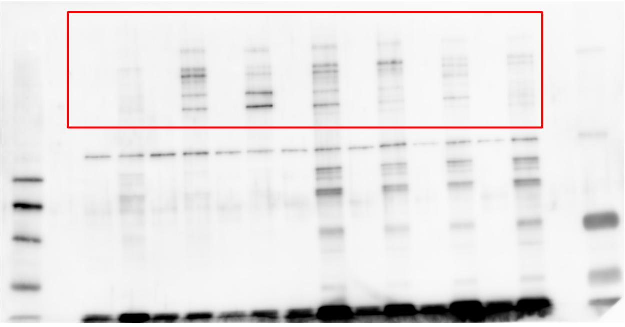

Figure 1-Source Data

Figure 1D

αLptD (no ME) Original image

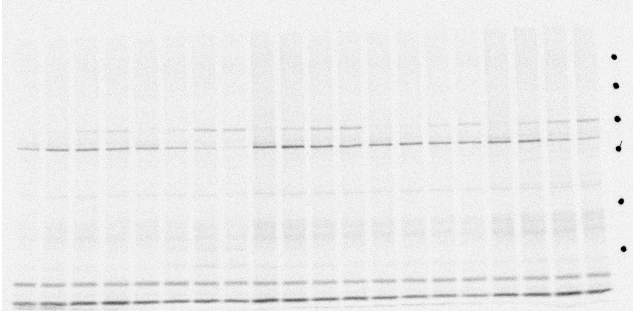

Used area

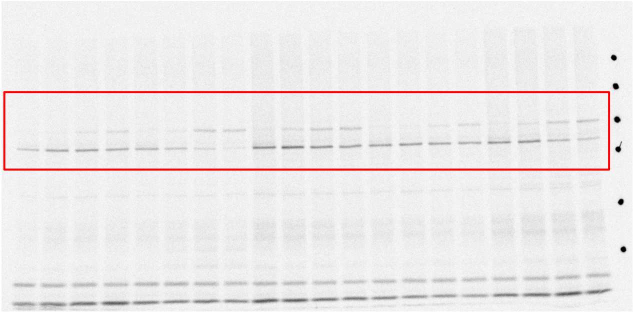

αLptD (+ ME) Original image

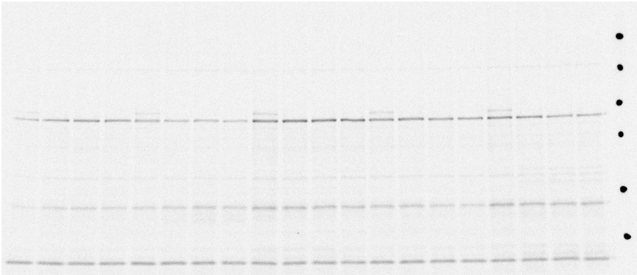

Used area

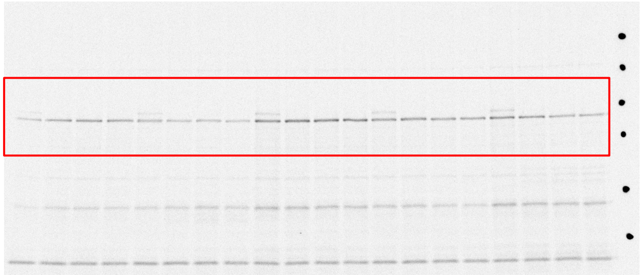

Figure 1D Source for quantitation

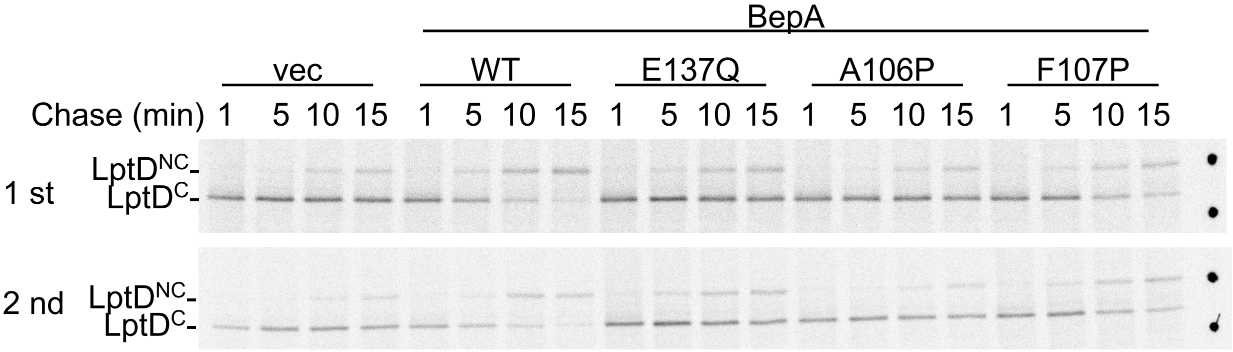

The "original" images were constructed by the image processing (including rotation, flip, contrast adjusting, and/or cropping) of the corresponding raw data.
