# Supplementary figures and images for: Edge-strand of BepA interacts with immature LptD on the β-barrel assembly machine to direct it to on- and off-pathways
Source: eLife. 2021 Aug 31;10:e70541. doi: 10.7554/eLife.70541 (PMC8423444; doi:10.7554/eLife.70541)

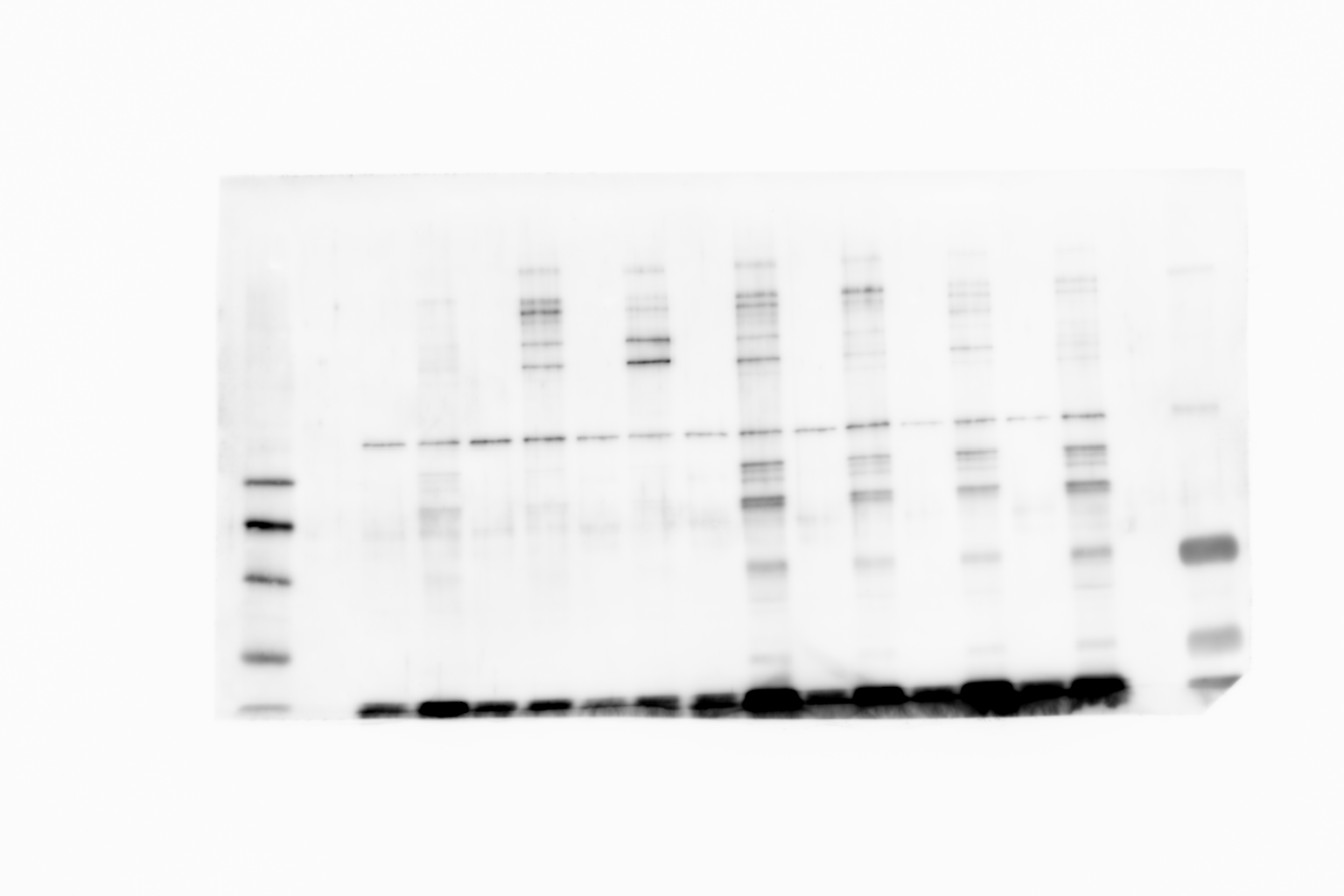

Supplement: Figure 1—source data 1. — (B–D) For the immunoblotting experiments using the anti-BepA and anti-LptD antibodies and quantified band intensity data for the pulse-chase experiments using the anti-LptD antibody. [file elife-70541-fig1-data1.zip › Figure 1 Source data files(revised)/Figure 1-Source data raw data/Figure 1-Source data 5 (C_raw data-LptD).tif]

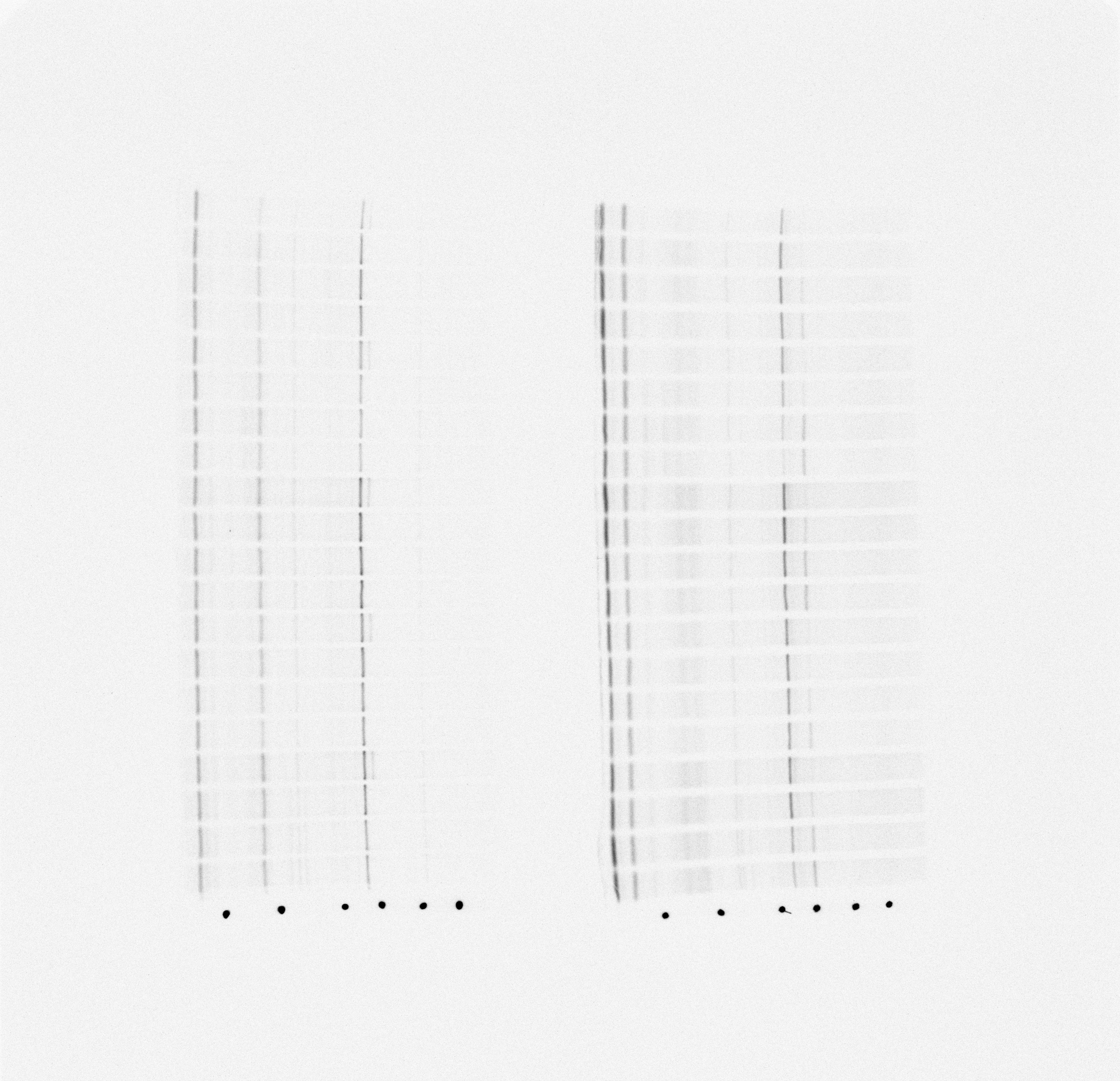

Supplement: Figure 1—source data 1. — (B–D) For the immunoblotting experiments using the anti-BepA and anti-LptD antibodies and quantified band intensity data for the pulse-chase experiments using the anti-LptD antibody. [file elife-70541-fig1-data1.zip › Figure 1 Source data files(revised)/Figure 1-Source data raw data/Figure 1-Source data 7 (D_raw data-2nd noME and +ME).tif]

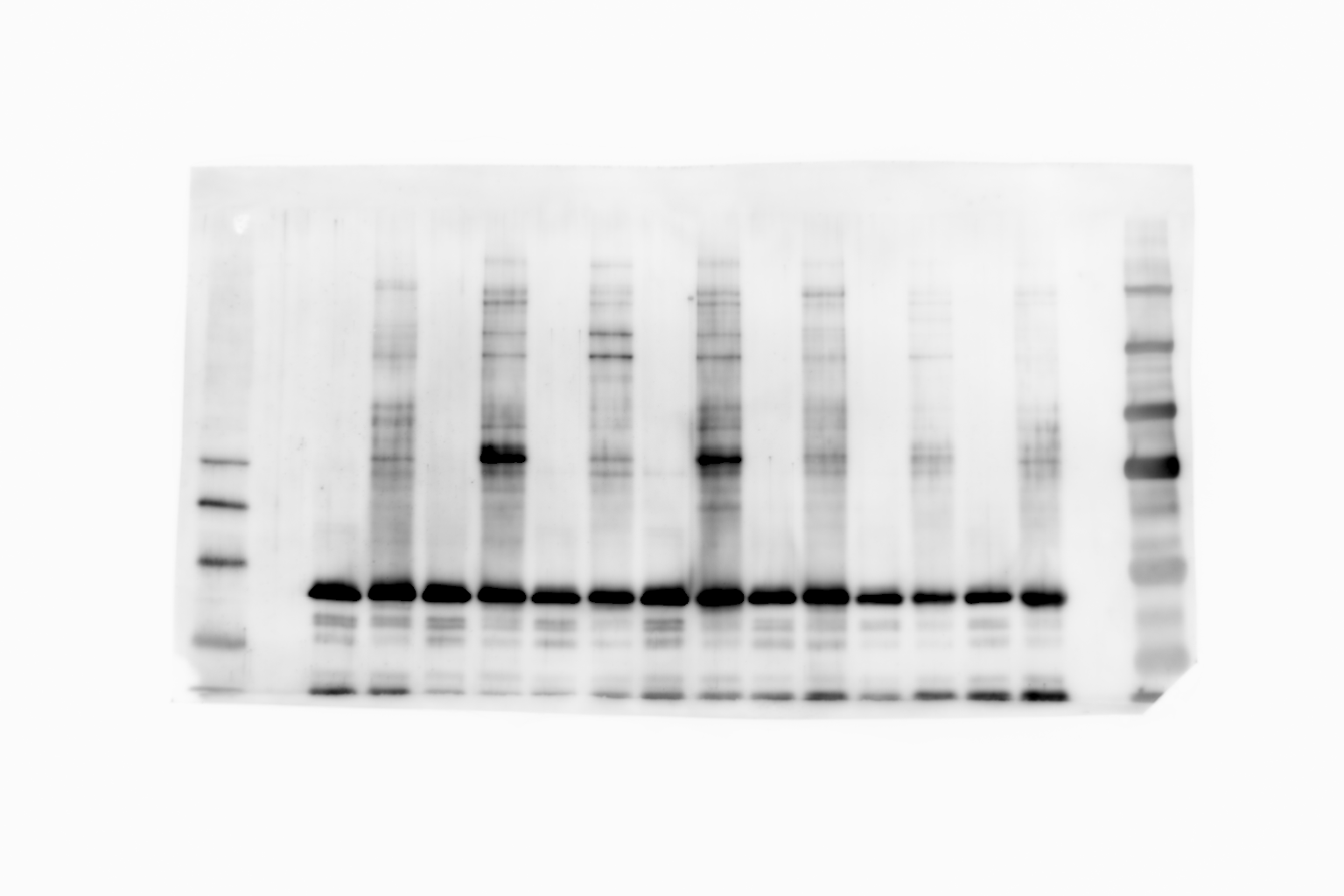

Supplement: Figure 1—source data 1. — (B–D) For the immunoblotting experiments using the anti-BepA and anti-LptD antibodies and quantified band intensity data for the pulse-chase experiments using the anti-LptD antibody. [file elife-70541-fig1-data1.zip › Figure 1 Source data files(revised)/Figure 1-Source data raw data/Figure 1-Source data 4(C_raw data-BepA).tif]

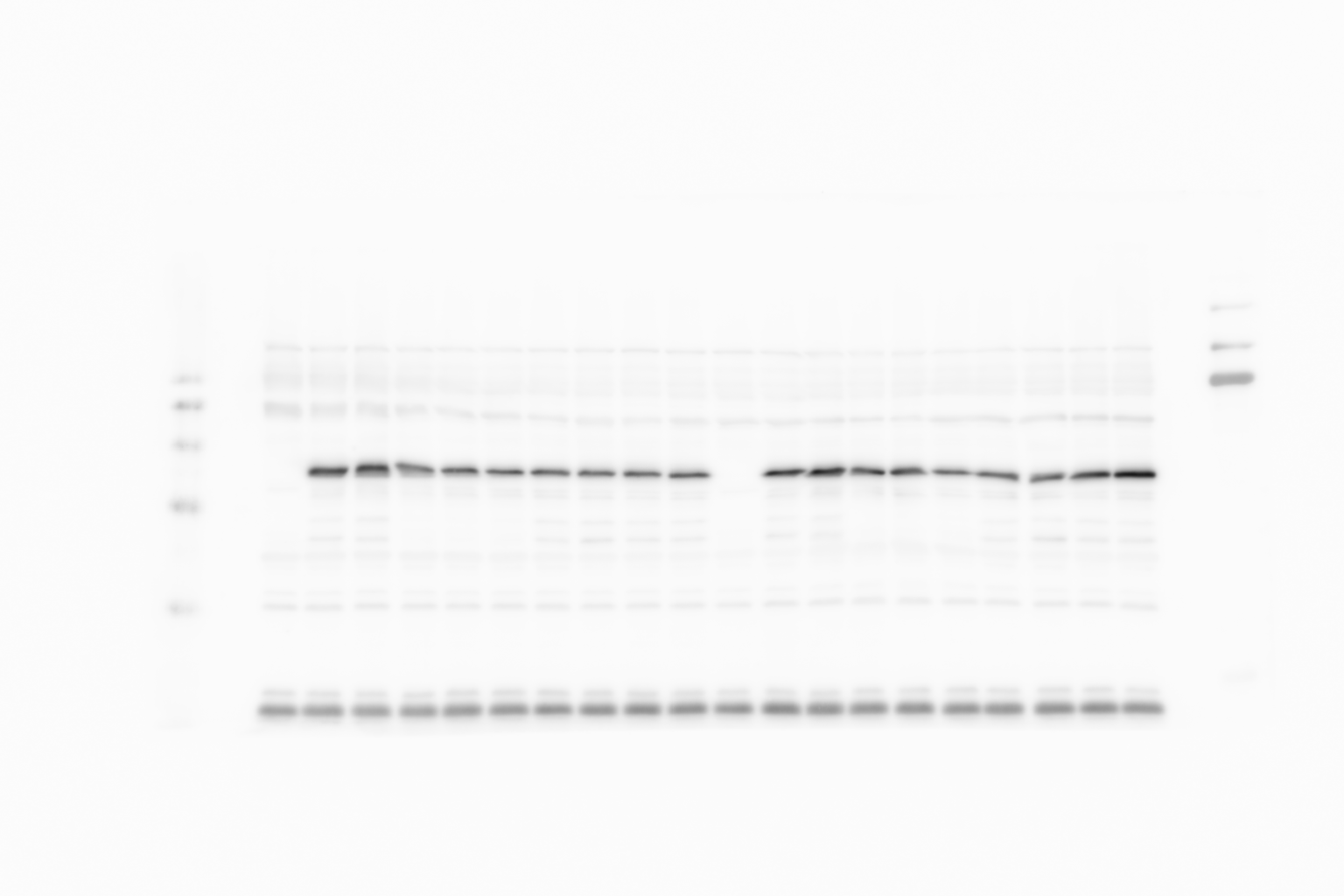

Supplement: Figure 1—source data 1. — (B–D) For the immunoblotting experiments using the anti-BepA and anti-LptD antibodies and quantified band intensity data for the pulse-chase experiments using the anti-LptD antibody. [file elife-70541-fig1-data1.zip › Figure 1 Source data files(revised)/Figure 1-Source data raw data/Figure 1-Source data 2 (B_raw data-BepA).tif]

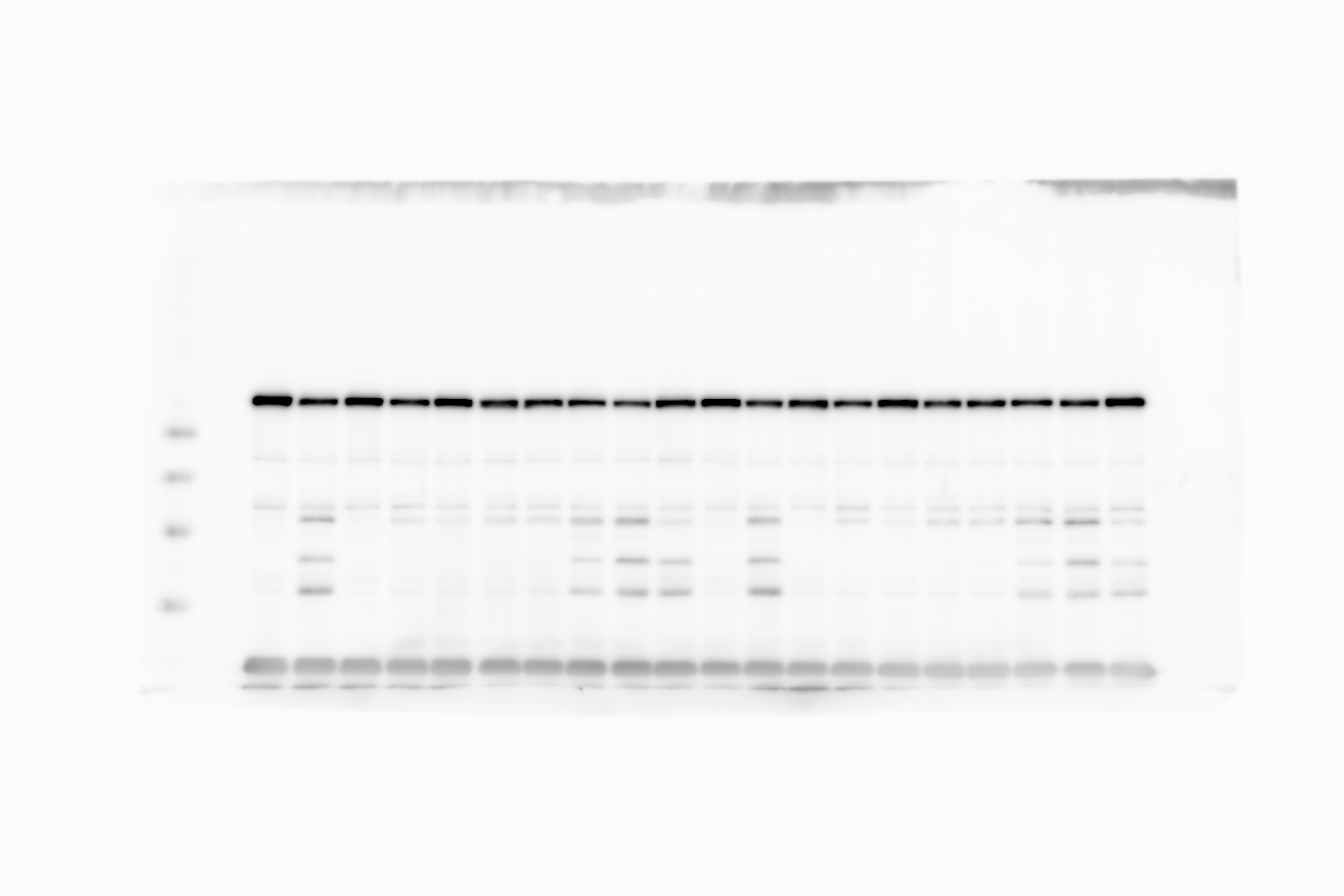

Supplement: Figure 1—source data 1. — (B–D) For the immunoblotting experiments using the anti-BepA and anti-LptD antibodies and quantified band intensity data for the pulse-chase experiments using the anti-LptD antibody. [file elife-70541-fig1-data1.zip › Figure 1 Source data files(revised)/Figure 1-Source data raw data/Figure 1-Source data 3 (B_raw data-LptD).tif]

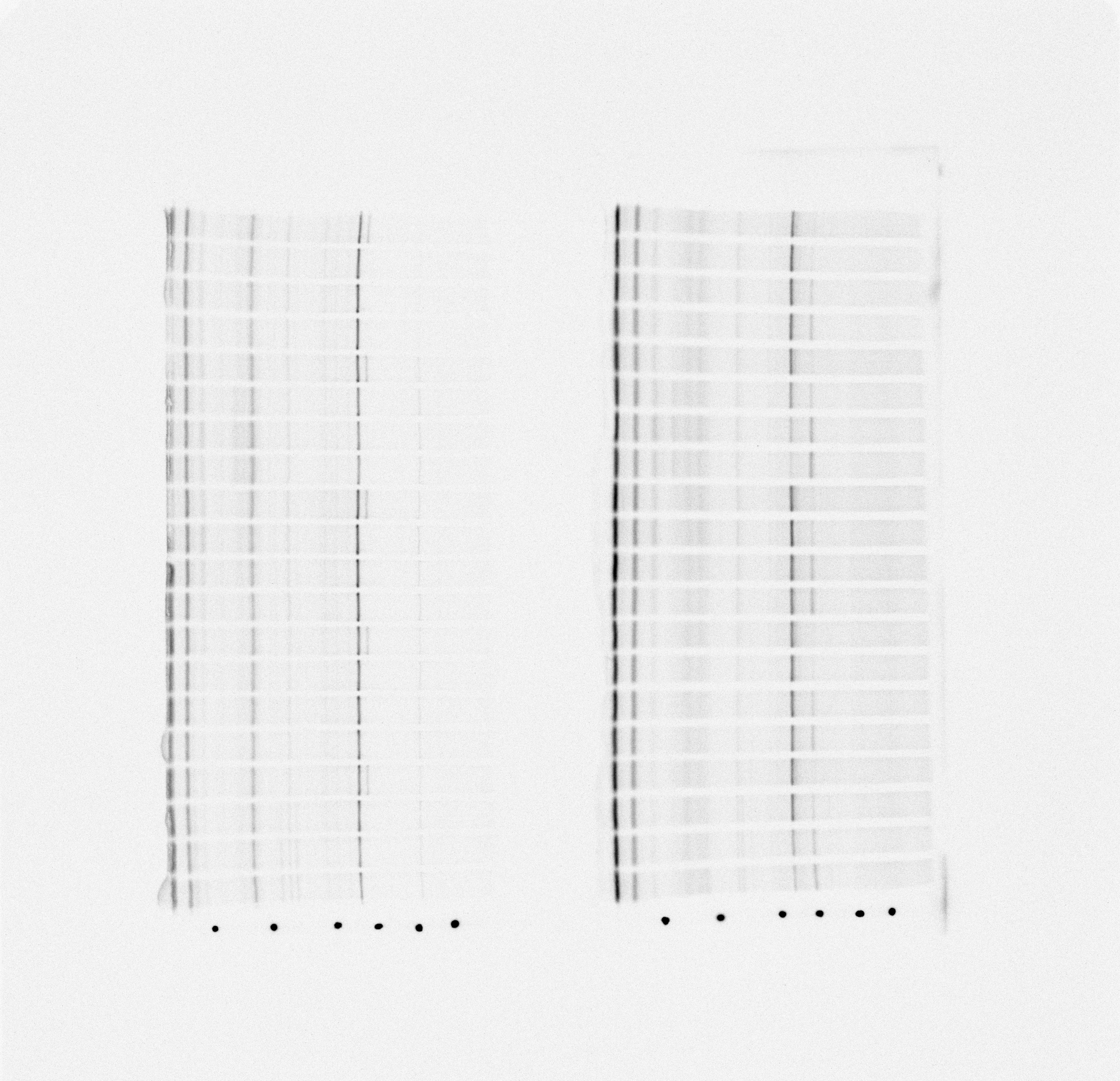

Supplement: Figure 1—source data 1. — (B–D) For the immunoblotting experiments using the anti-BepA and anti-LptD antibodies and quantified band intensity data for the pulse-chase experiments using the anti-LptD antibody. [file elife-70541-fig1-data1.zip › Figure 1 Source data files(revised)/Figure 1-Source data raw data/Figure 1-Source data 6 (D_raw data-1st noME and +ME).tif]

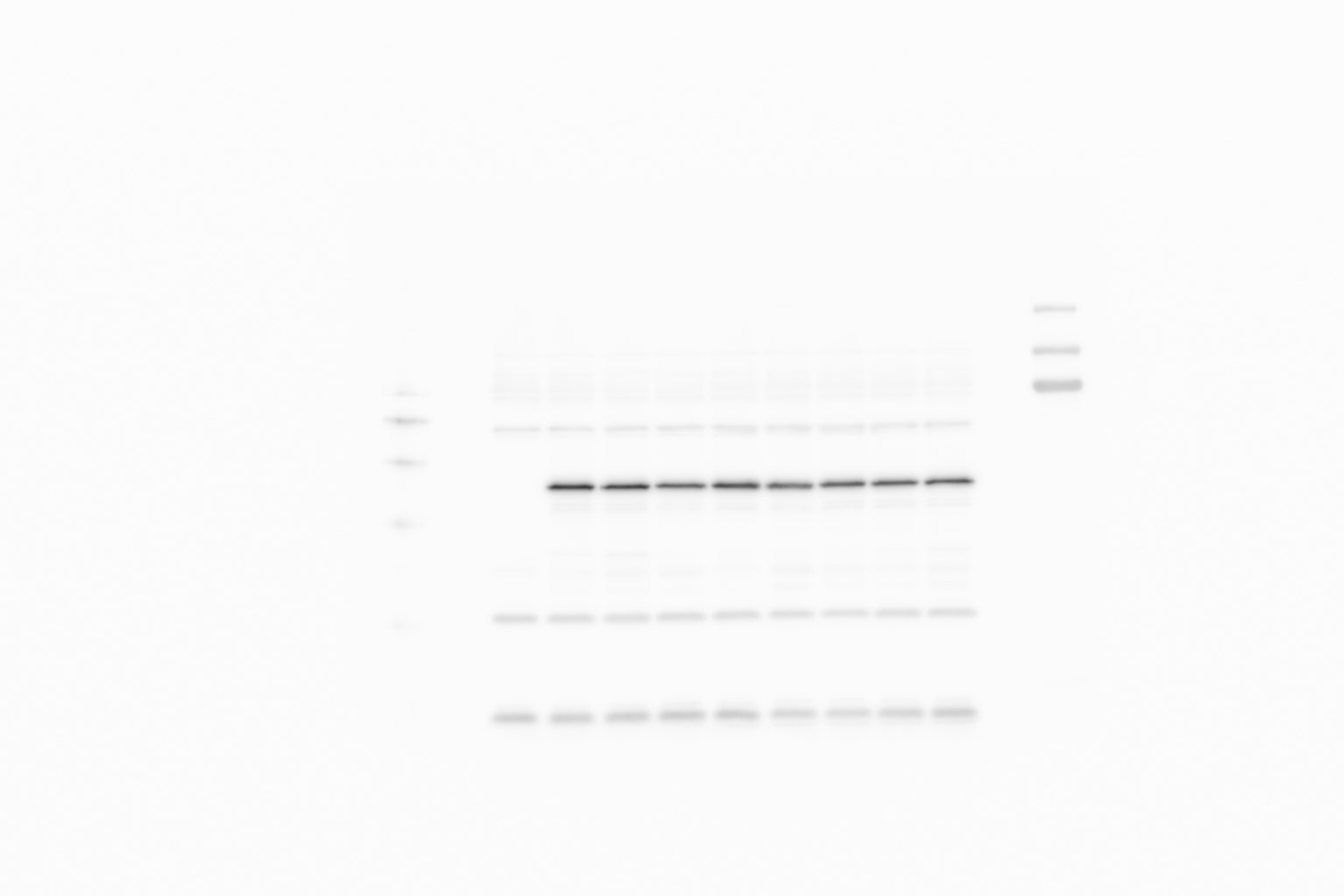

Supplement: Figure 1—figure supplement 2—source data 1. [file elife-70541-fig1-figsupp2-data1.zip › Figure 1-figure supplement 2 Source data files/Figure 1-figure supplement 2-Source data (raw data)/Figure 1-figure supplement 2-Source data 3 (A_raw data-BepA).tif]

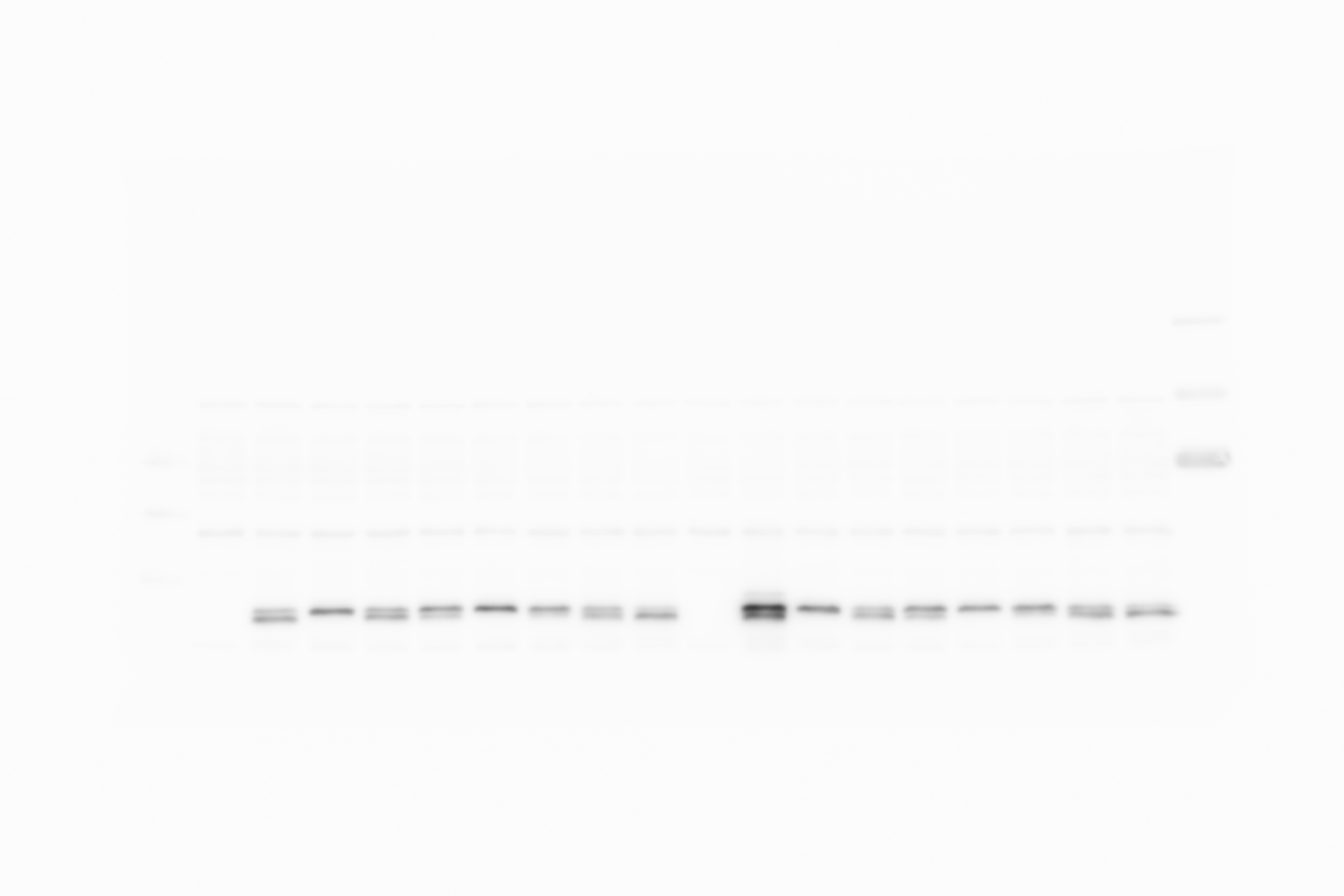

Supplement: Figure 1—figure supplement 2—source data 1. [file elife-70541-fig1-figsupp2-data1.zip › Figure 1-figure supplement 2 Source data files/Figure 1-figure supplement 2-Source data (raw data)/Figure 1-figure supplement 2-Source data 4 (B_raw data-BepA).tif]

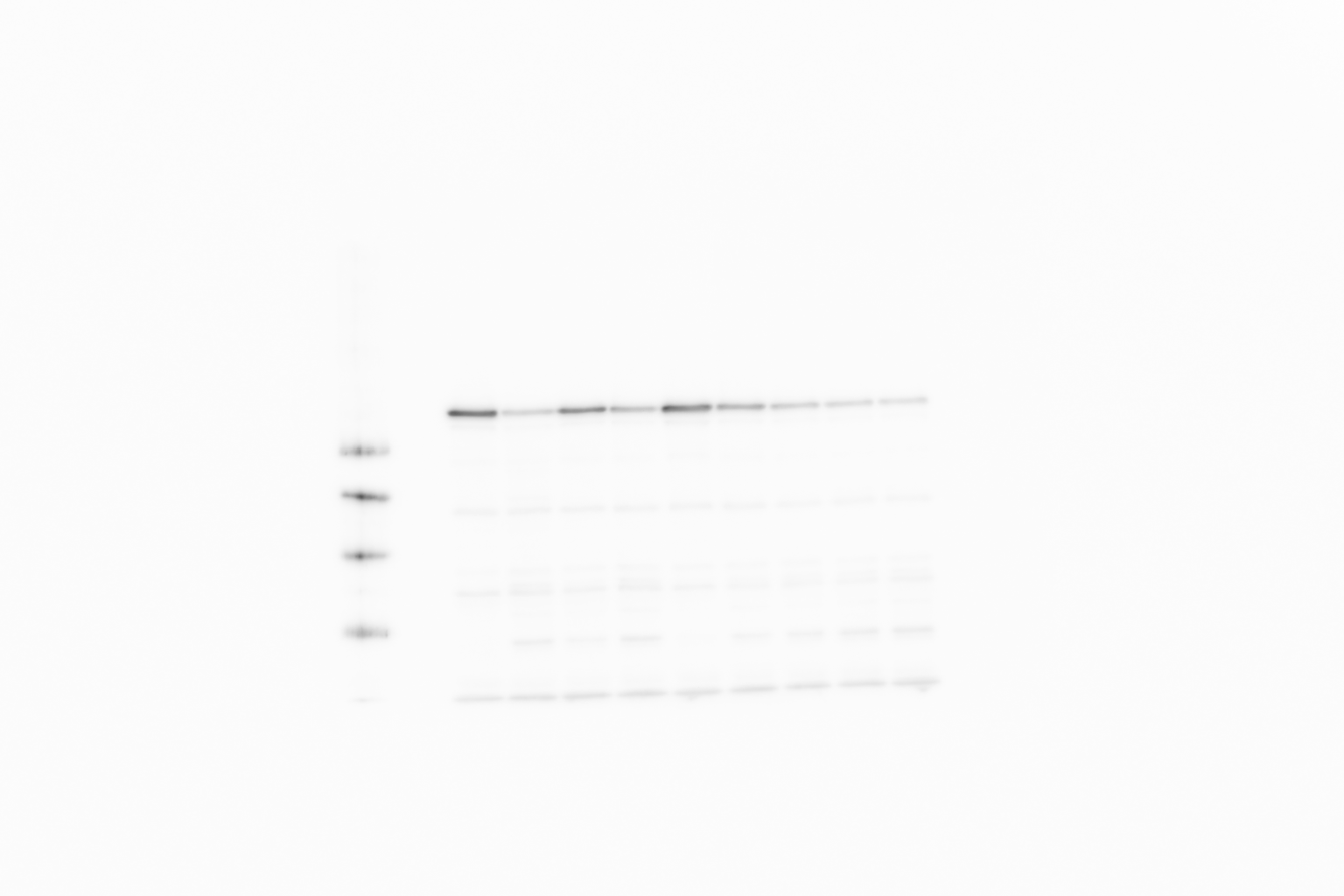

Supplement: Figure 1—figure supplement 2—source data 1. [file elife-70541-fig1-figsupp2-data1.zip › Figure 1-figure supplement 2 Source data files/Figure 1-figure supplement 2-Source data (raw data)/Figure 1-figure supplement 2-Source data 2 (A_raw data-BamA).tif]

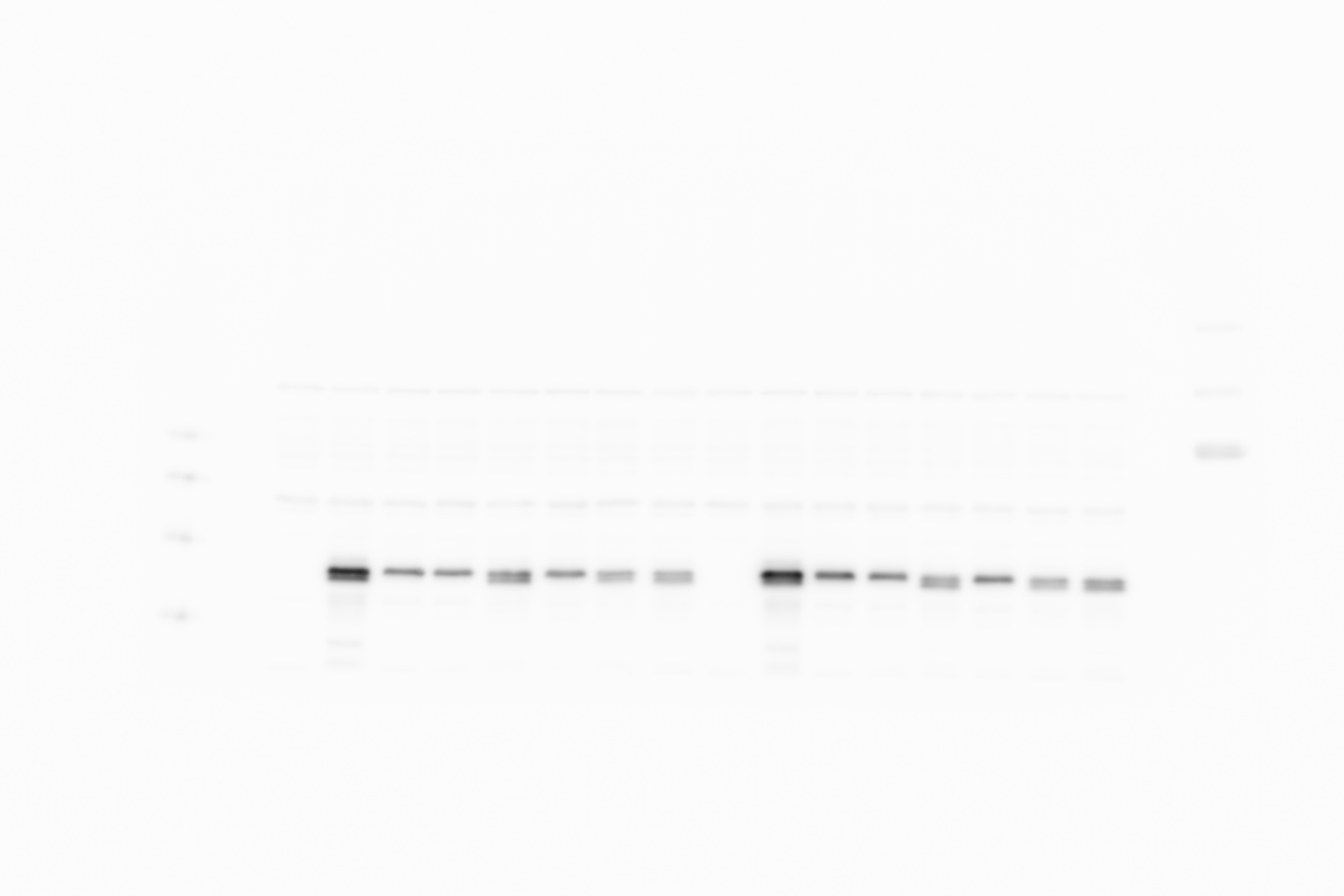

Supplement: Figure 1—figure supplement 3—source data 1. [file elife-70541-fig1-figsupp3-data1.zip › Figure 1-figure supplment 3 Source data files/Figure 1-figure supplement 3-Source data (raw data)/Figure 1-figure supplement 3-Source data 3 (B_raw data-BepA).tif]

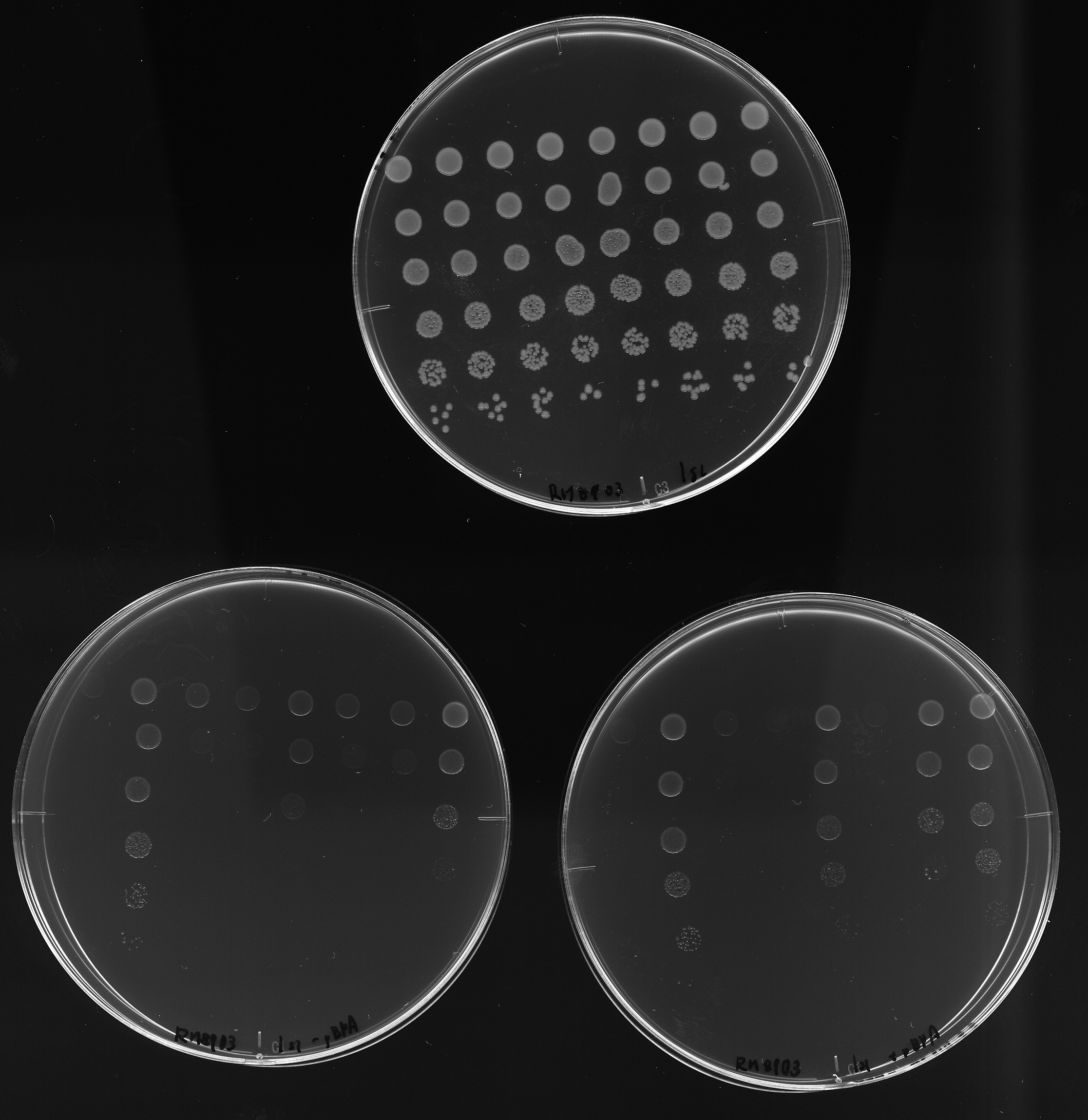

Supplement: Figure 1—figure supplement 3—source data 1. [file elife-70541-fig1-figsupp3-data1.zip › Figure 1-figure supplment 3 Source data files/Figure 1-figure supplement 3-Source data (raw data)/Figure 1-figure supplement 3-Source data 2 (A_raw data).jpg]

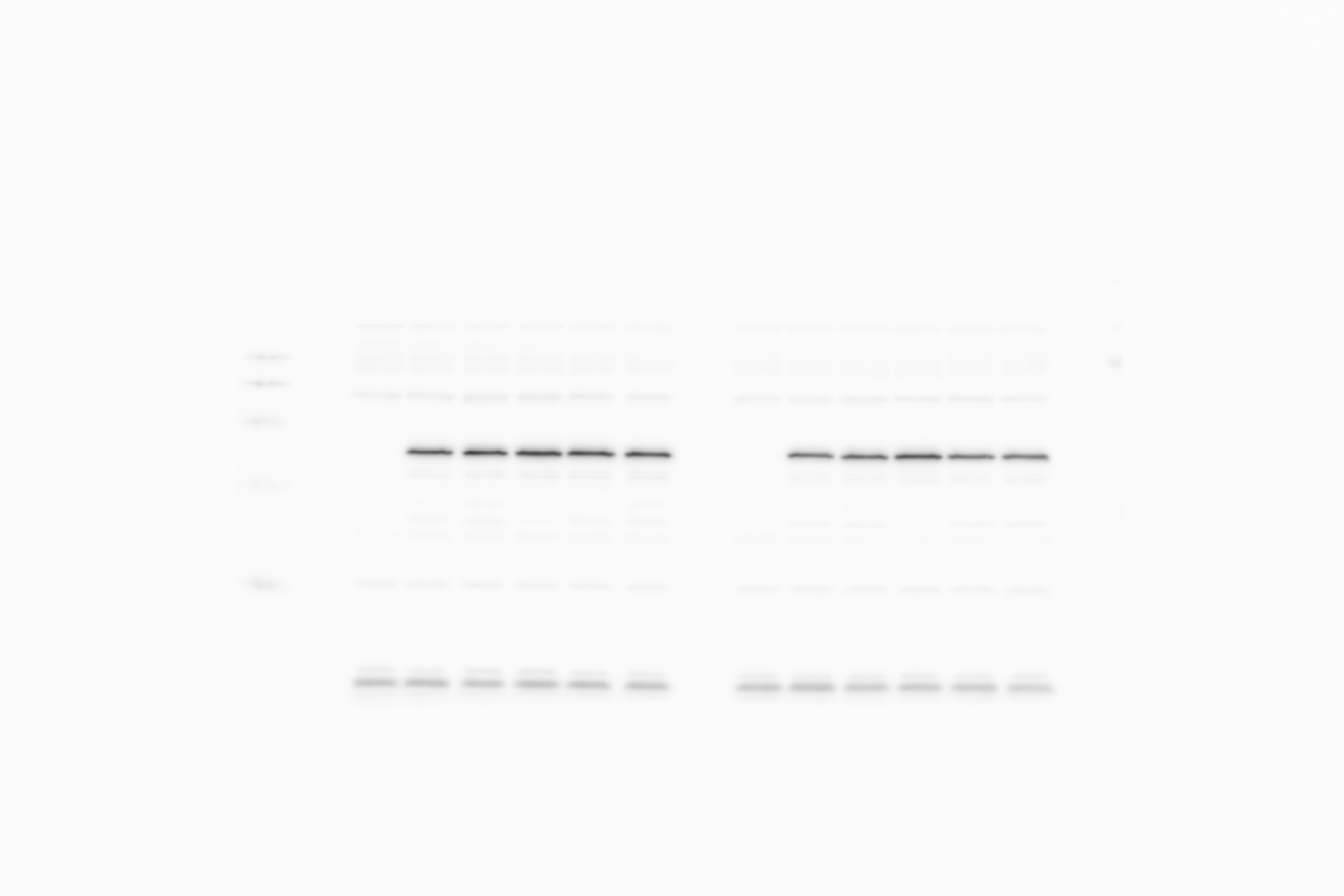

Supplement: Figure 1—figure supplement 4—source data 1. [file elife-70541-fig1-figsupp4-data1.zip › Figure 1-figure supplment 4 Source data files/Figure 1-figure supplement 4-Source data (raw data)/Figure 1-figure supplement 4-Source data 6 (C_raw data-BepA).tif]

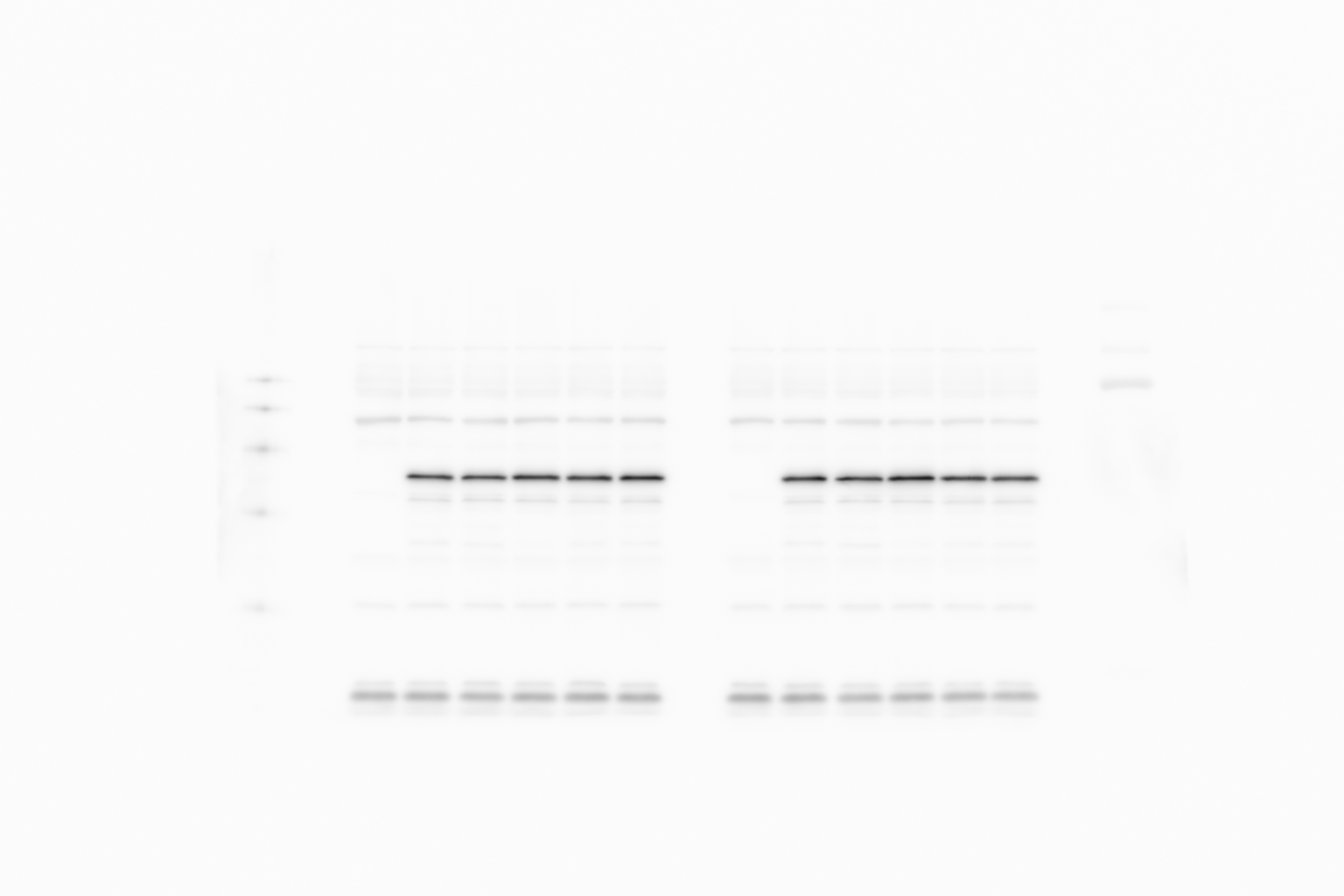

Supplement: Figure 1—figure supplement 4—source data 1. [file elife-70541-fig1-figsupp4-data1.zip › Figure 1-figure supplment 4 Source data files/Figure 1-figure supplement 4-Source data (raw data)/Figure 1-figure supplement 4-Source data 4 (B_raw data-BepA).tif]

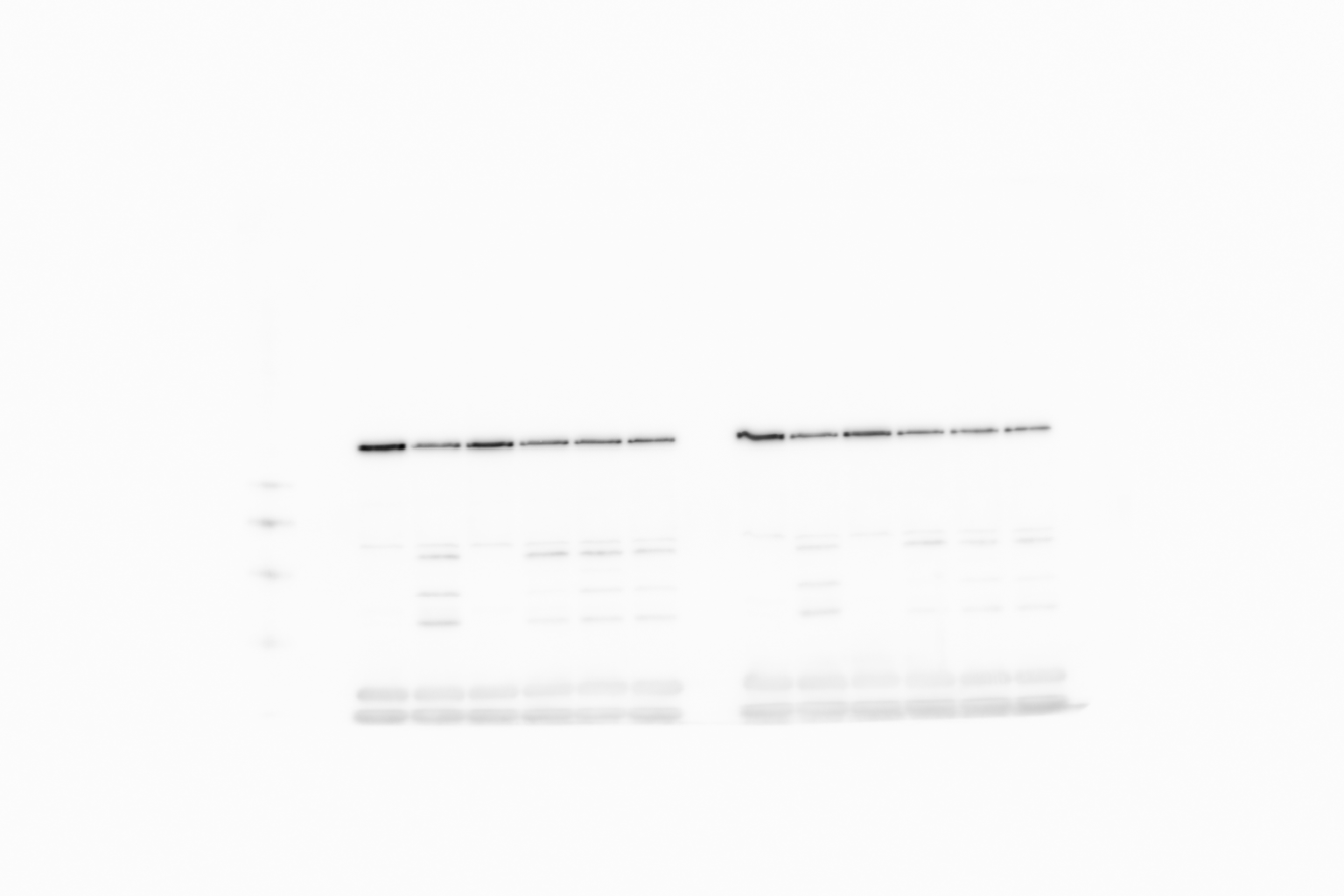

Supplement: Figure 1—figure supplement 4—source data 1. [file elife-70541-fig1-figsupp4-data1.zip › Figure 1-figure supplment 4 Source data files/Figure 1-figure supplement 4-Source data (raw data)/Figure 1-figure supplement 4-Source data 7(C_raw data-LptD).tif]

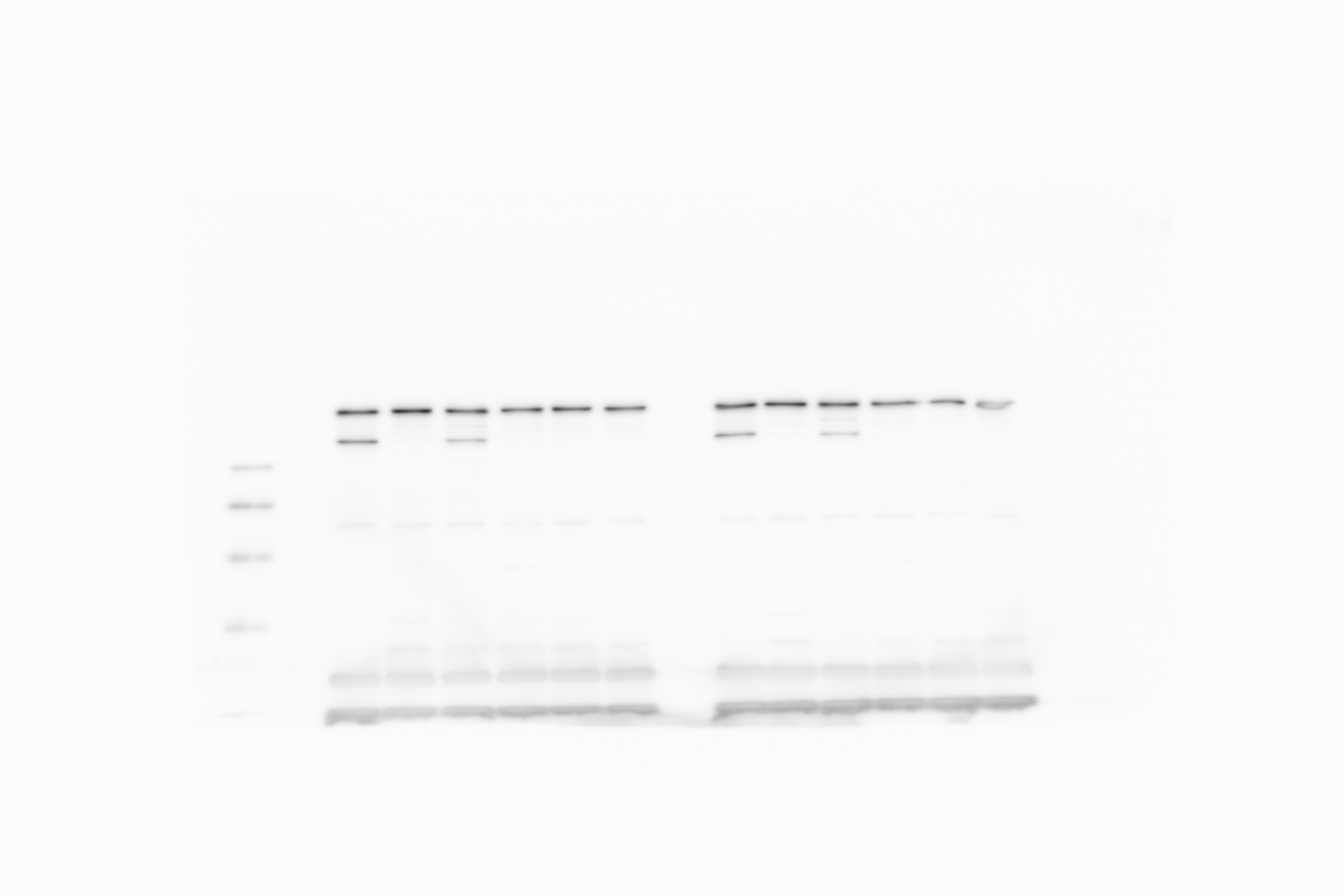

Supplement: Figure 1—figure supplement 4—source data 1. [file elife-70541-fig1-figsupp4-data1.zip › Figure 1-figure supplment 4 Source data files/Figure 1-figure supplement 4-Source data (raw data)/Figure 1-figure supplement 4-Source data 5 (B_raw data-LptD).tif]

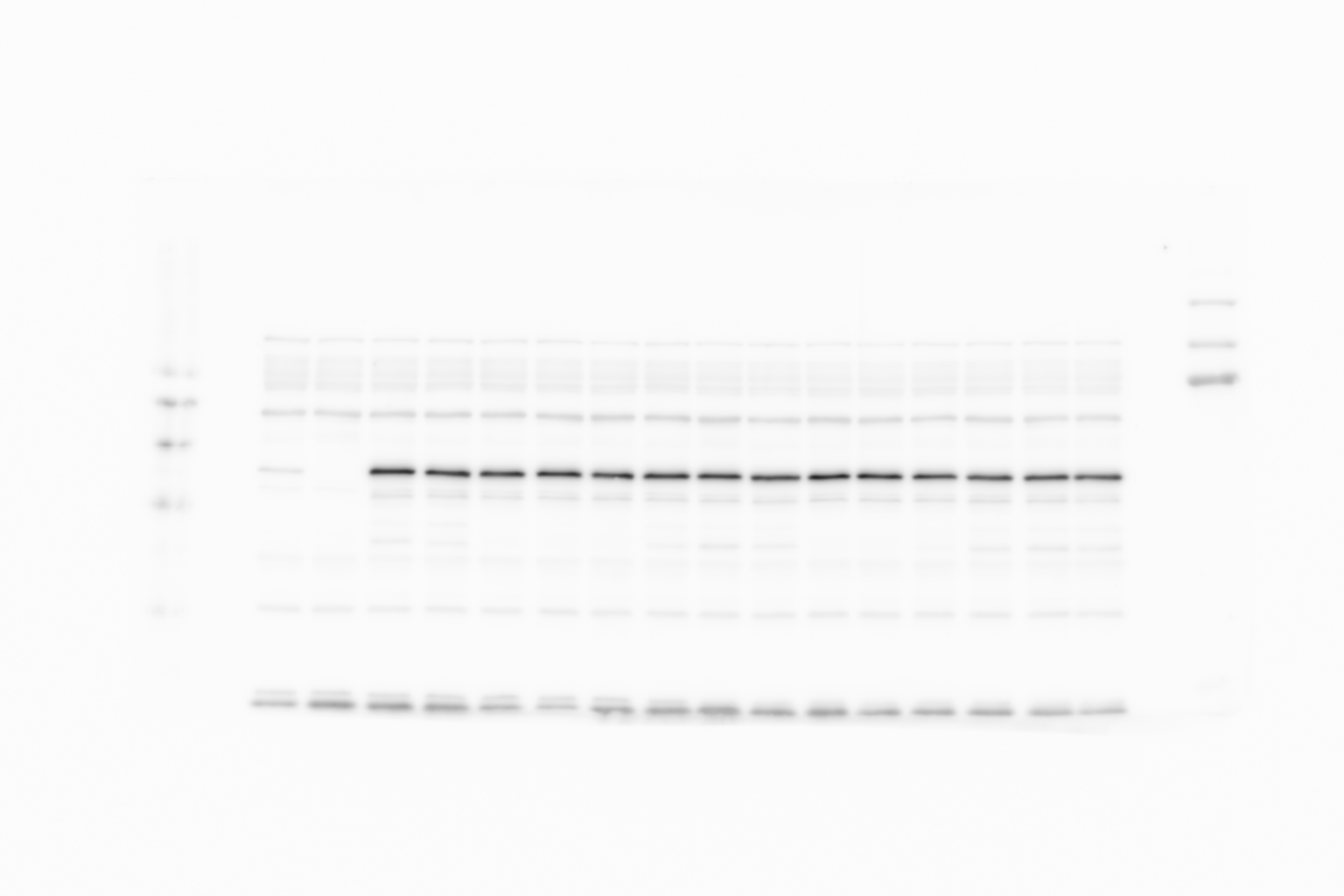

Supplement: Figure 1—figure supplement 4—source data 1. [file elife-70541-fig1-figsupp4-data1.zip › Figure 1-figure supplment 4 Source data files/Figure 1-figure supplement 4-Source data (raw data)/Figure 1-figure supplement 4-Source data 2 (A_raw data-BepA).tif]

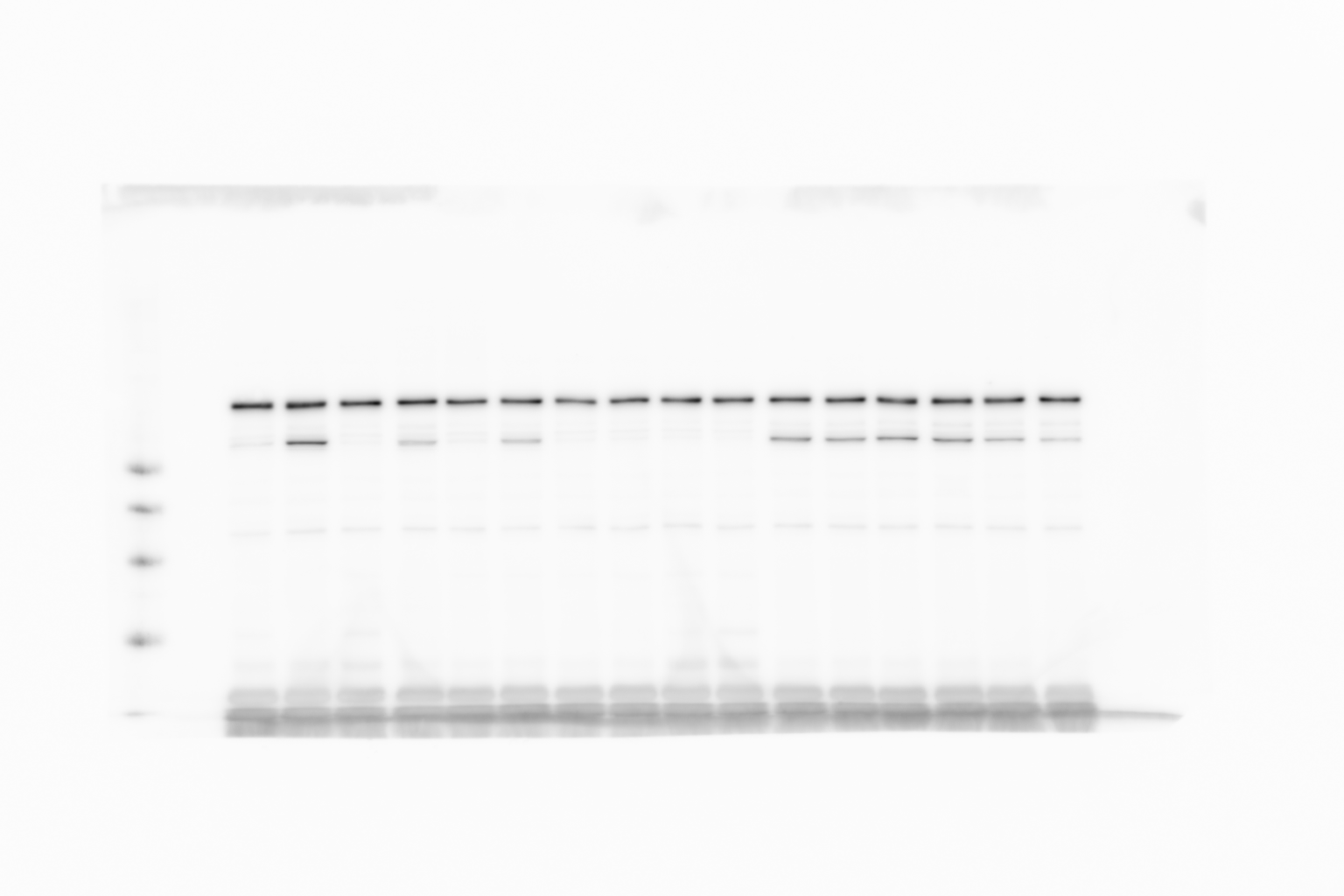

Supplement: Figure 1—figure supplement 4—source data 1. [file elife-70541-fig1-figsupp4-data1.zip › Figure 1-figure supplment 4 Source data files/Figure 1-figure supplement 4-Source data (raw data)/Figure 1-figure supplement 4-Source data 3 (A_raw data-LptD).tif]

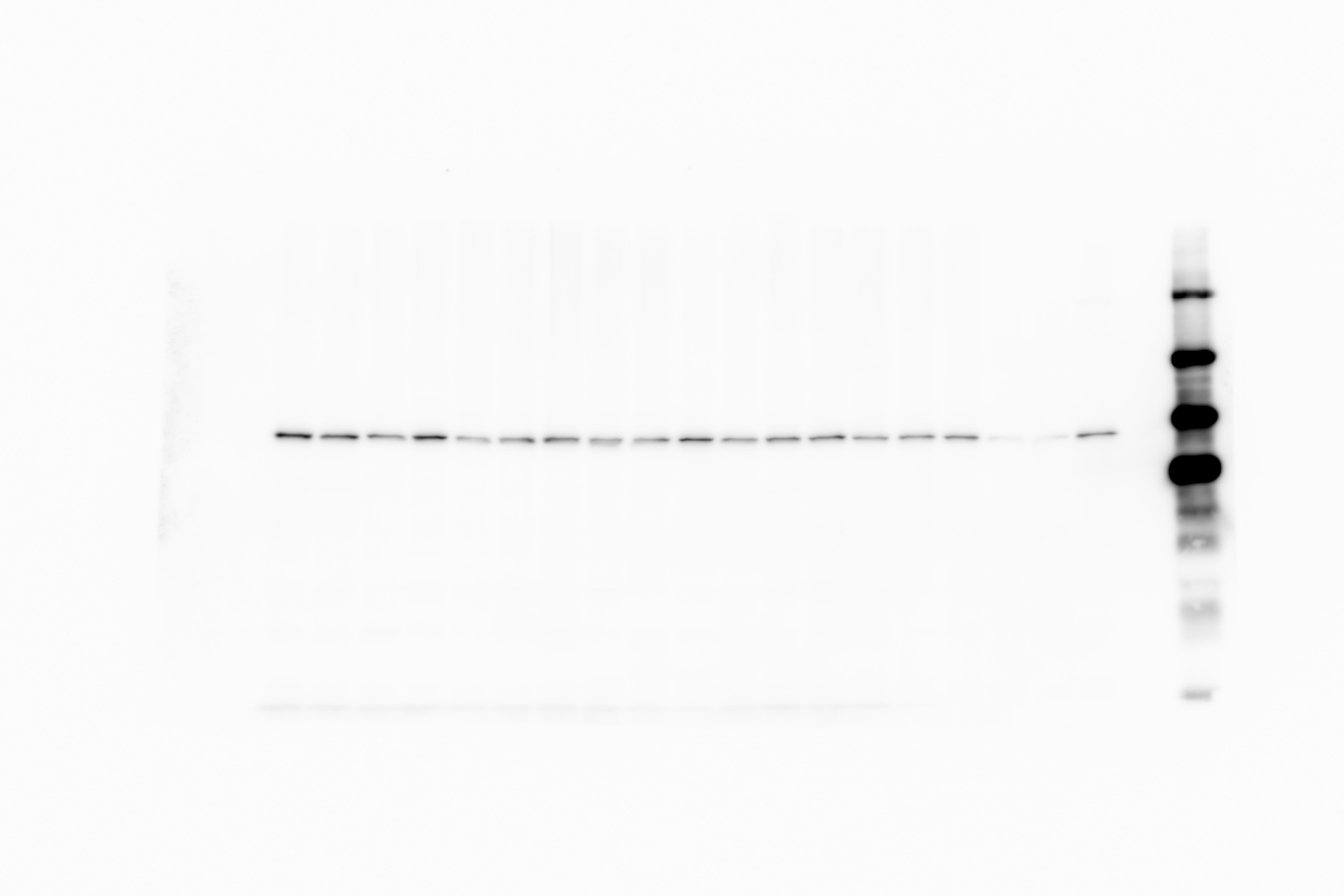

Supplement: Figure 2—source data 1. [file elife-70541-fig2-data1.zip › Figure 2 Source data files/Figure 2-Source data (raw data)/Figure 2-Source data 3 (A_raw data-beta-C His).tif]

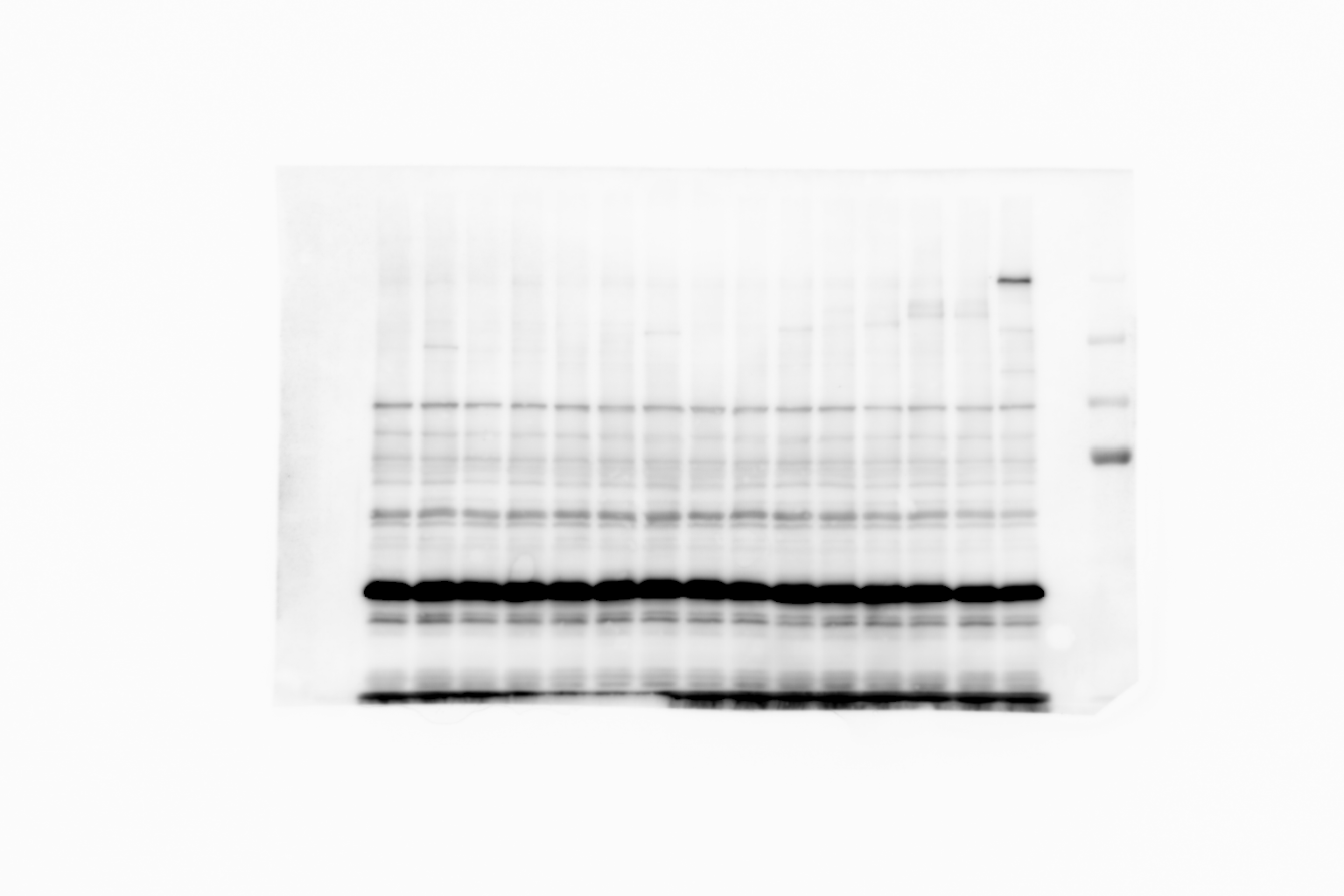

Supplement: Figure 2—source data 1. [file elife-70541-fig2-data1.zip › Figure 2 Source data files/Figure 2-Source data (raw data)/Figure 2-Source data 6 (A_raw data-Peri BepA).tif]

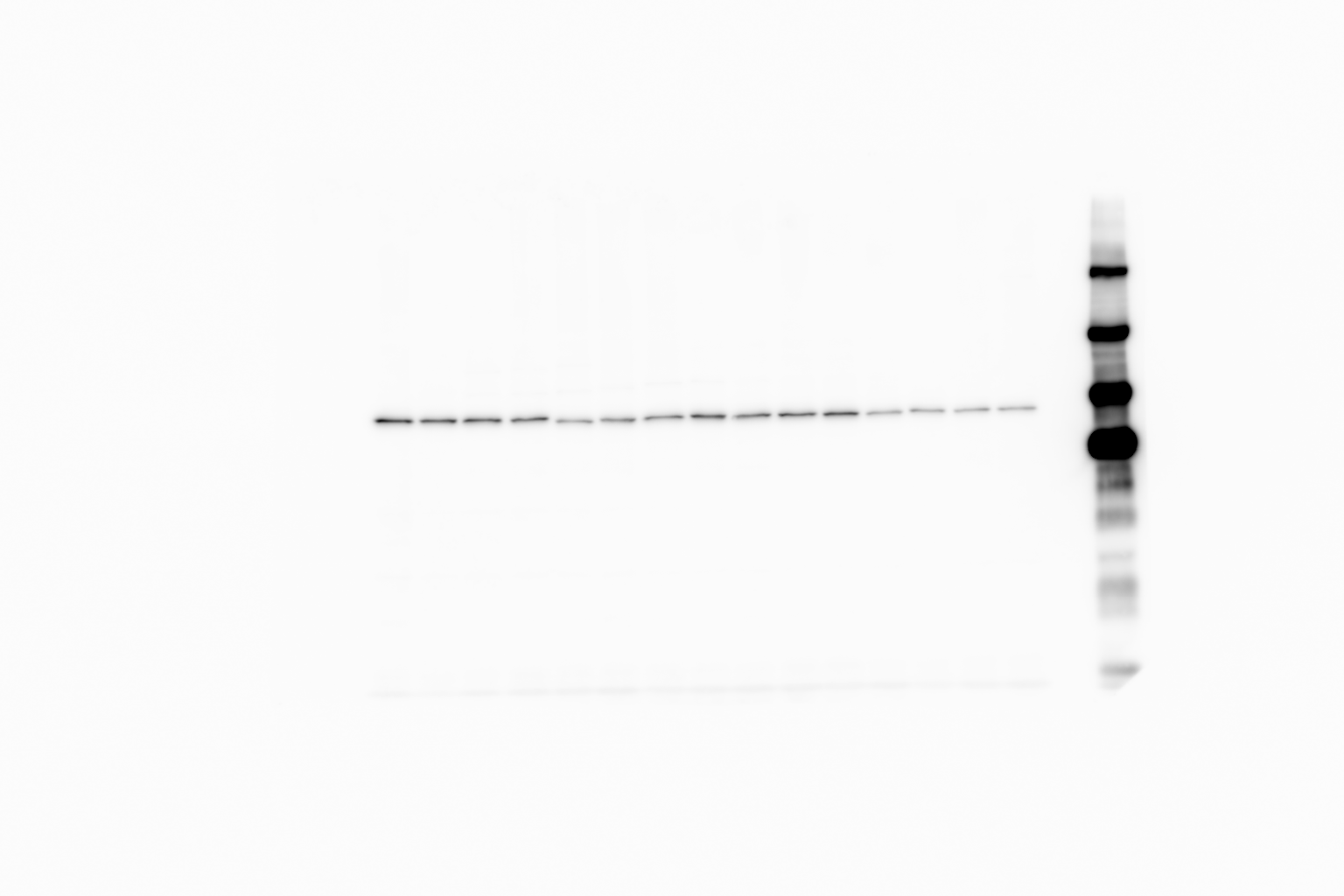

Supplement: Figure 2—source data 1. [file elife-70541-fig2-data1.zip › Figure 2 Source data files/Figure 2-Source data (raw data)/Figure 2-Source data 7 (A_raw data-Peri His).tif]

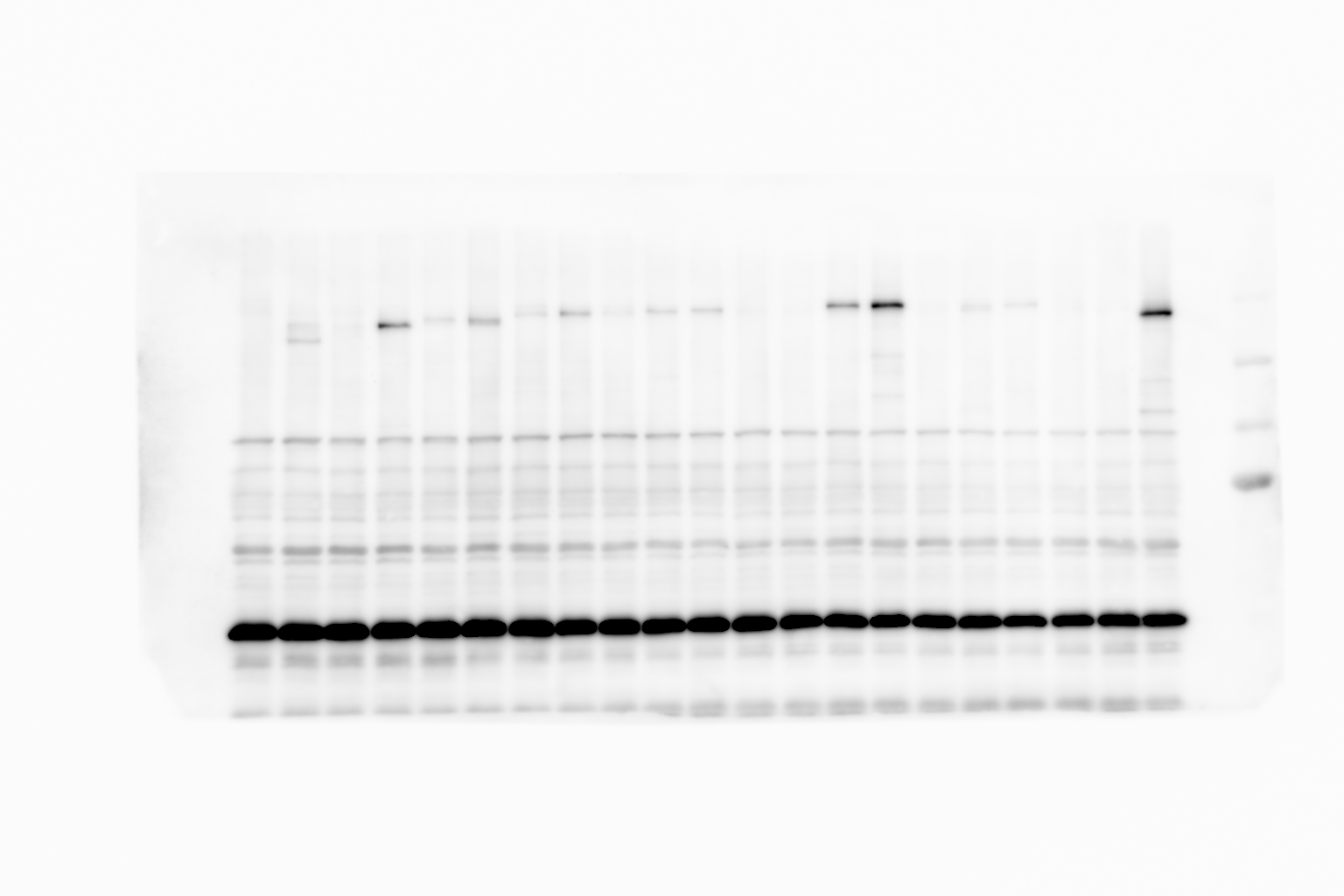

Supplement: Figure 2—source data 1. [file elife-70541-fig2-data1.zip › Figure 2 Source data files/Figure 2-Source data (raw data)/Figure 2-Source data 4 (A_raw data-beta-N BepA).tif]

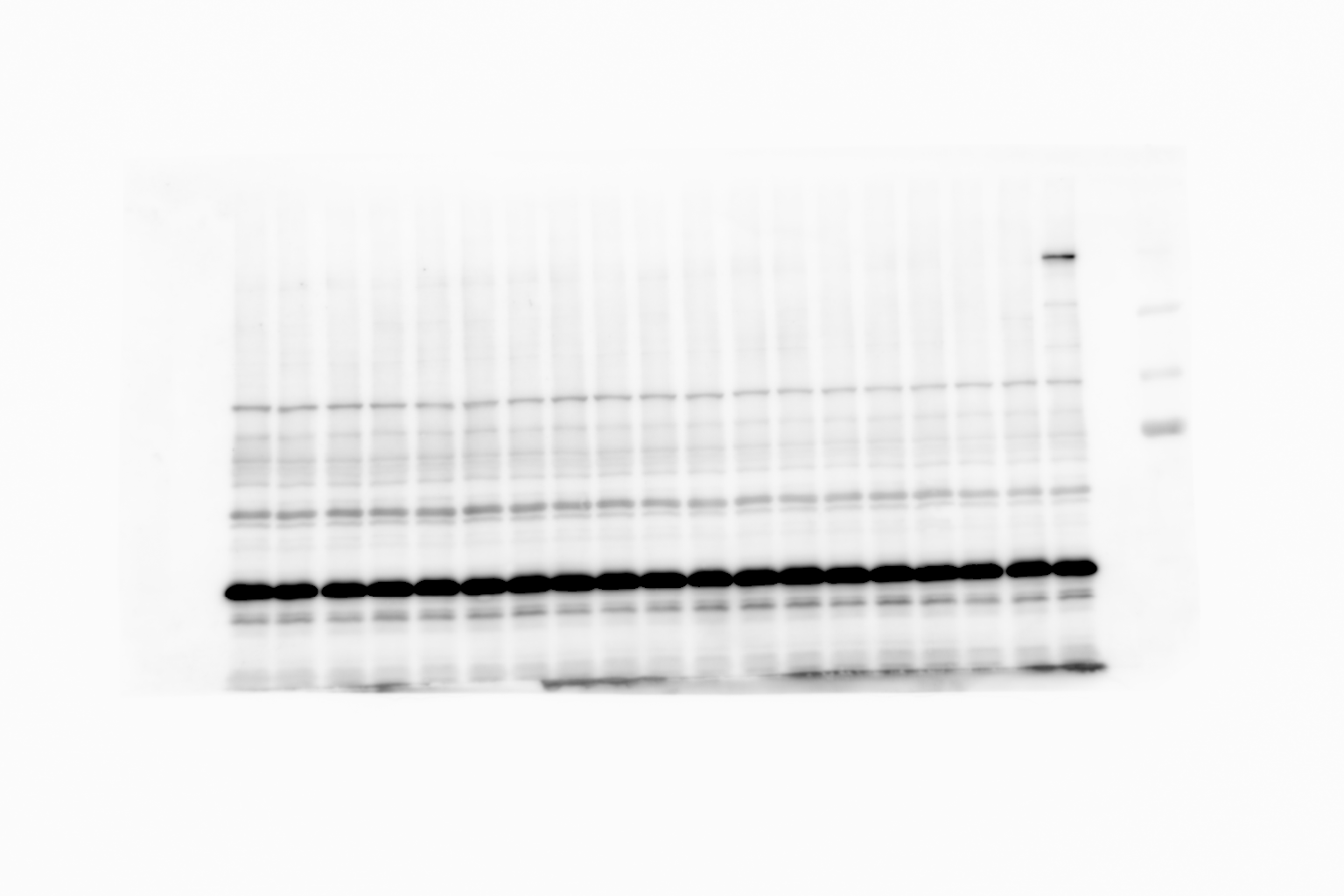

Supplement: Figure 2—source data 1. [file elife-70541-fig2-data1.zip › Figure 2 Source data files/Figure 2-Source data (raw data)/Figure 2-Source data 2 (A_raw data-beta-C BepA).tif]

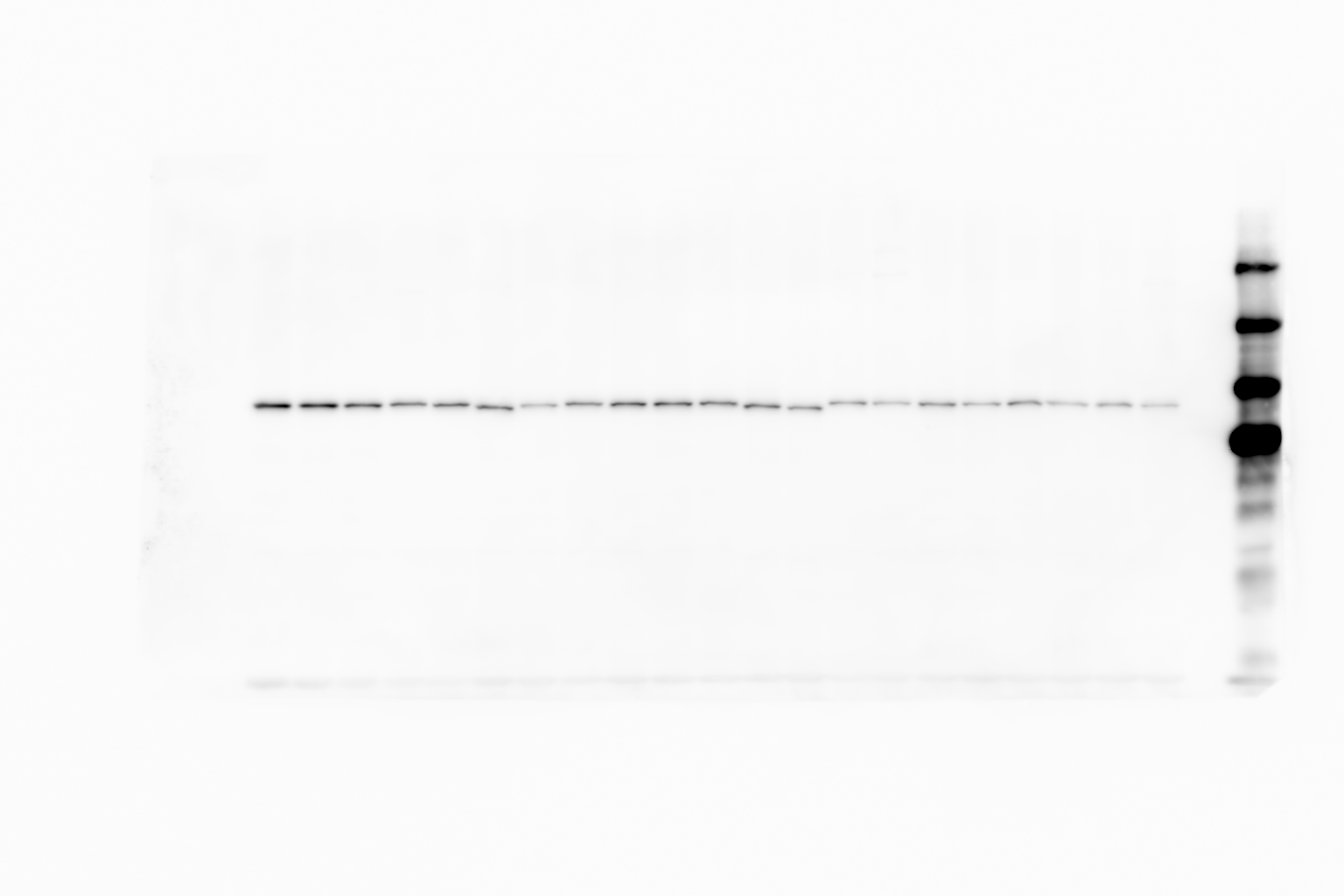

Supplement: Figure 2—source data 1. [file elife-70541-fig2-data1.zip › Figure 2 Source data files/Figure 2-Source data (raw data)/Figure 2-Source data 5 (A_raw data-beta-N His).tif]

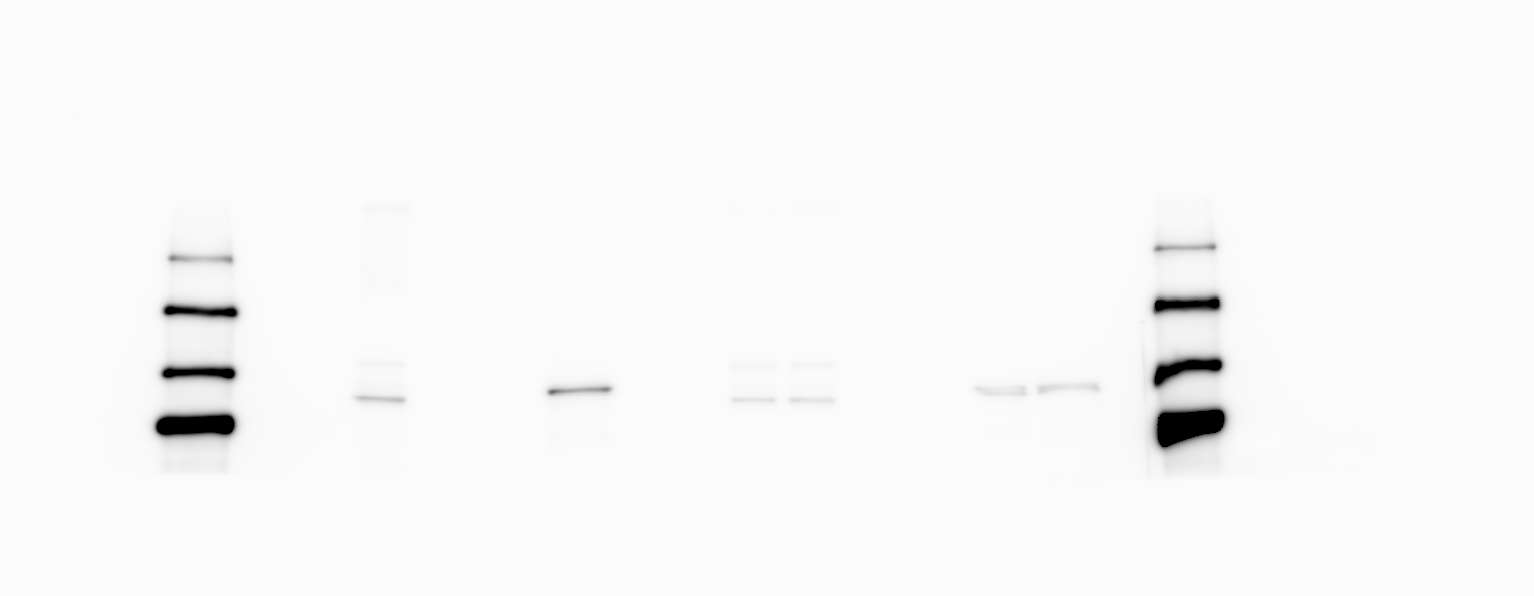

Supplement: Figure 2—figure supplement 1—source data 1. [file elife-70541-fig2-figsupp1-data1.zip › Figure 2-figure supplment 1 Source data files/Figure 2-figure supplement 1-Source data (raw data)/Figure 2-figure supplement 1-Source data 3 (raw data-His).tif]

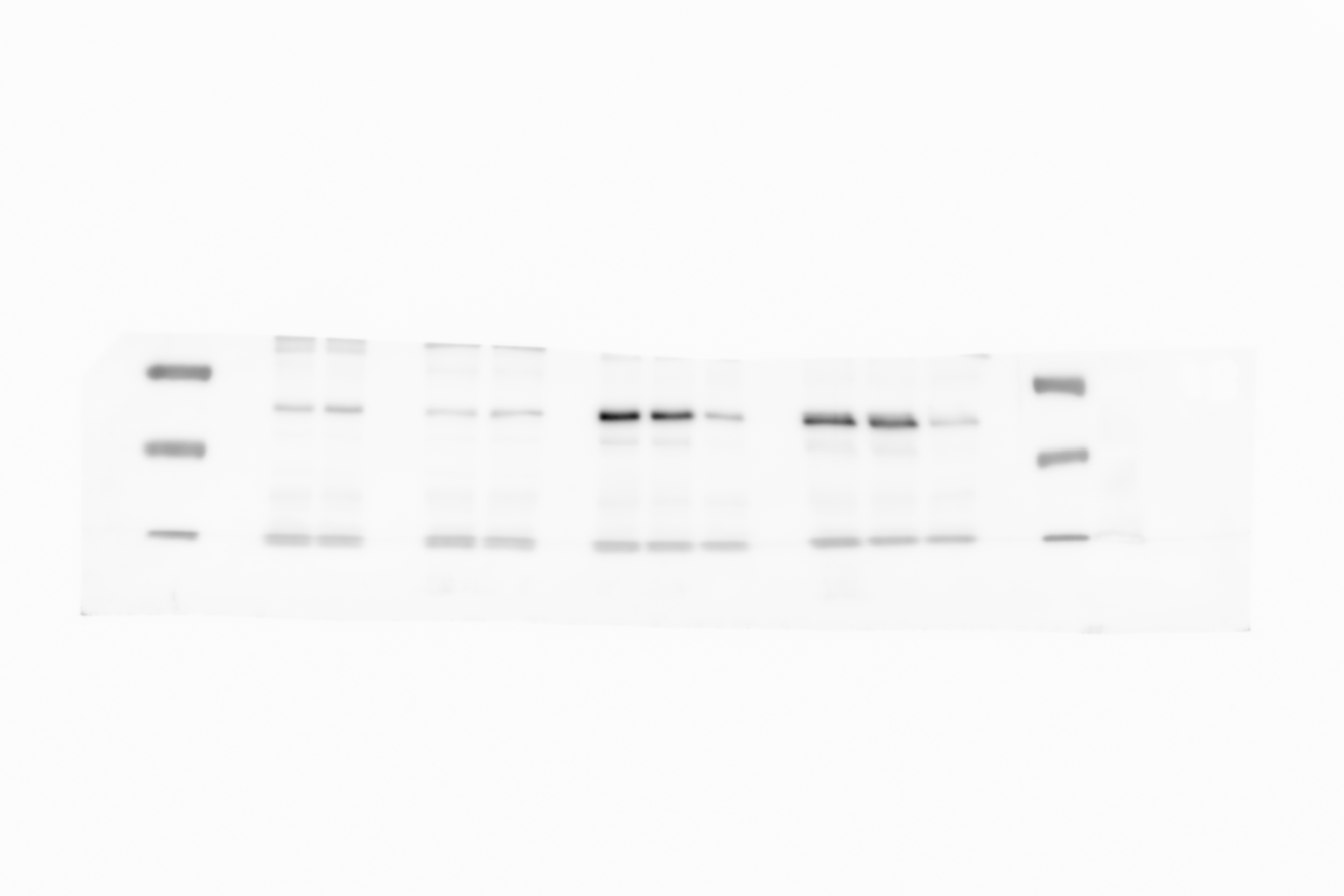

Supplement: Figure 2—figure supplement 1—source data 1. [file elife-70541-fig2-figsupp1-data1.zip › Figure 2-figure supplment 1 Source data files/Figure 2-figure supplement 1-Source data (raw data)/Figure 2-figure supplement 1-Source data 2 (raw data-BepA).tif]

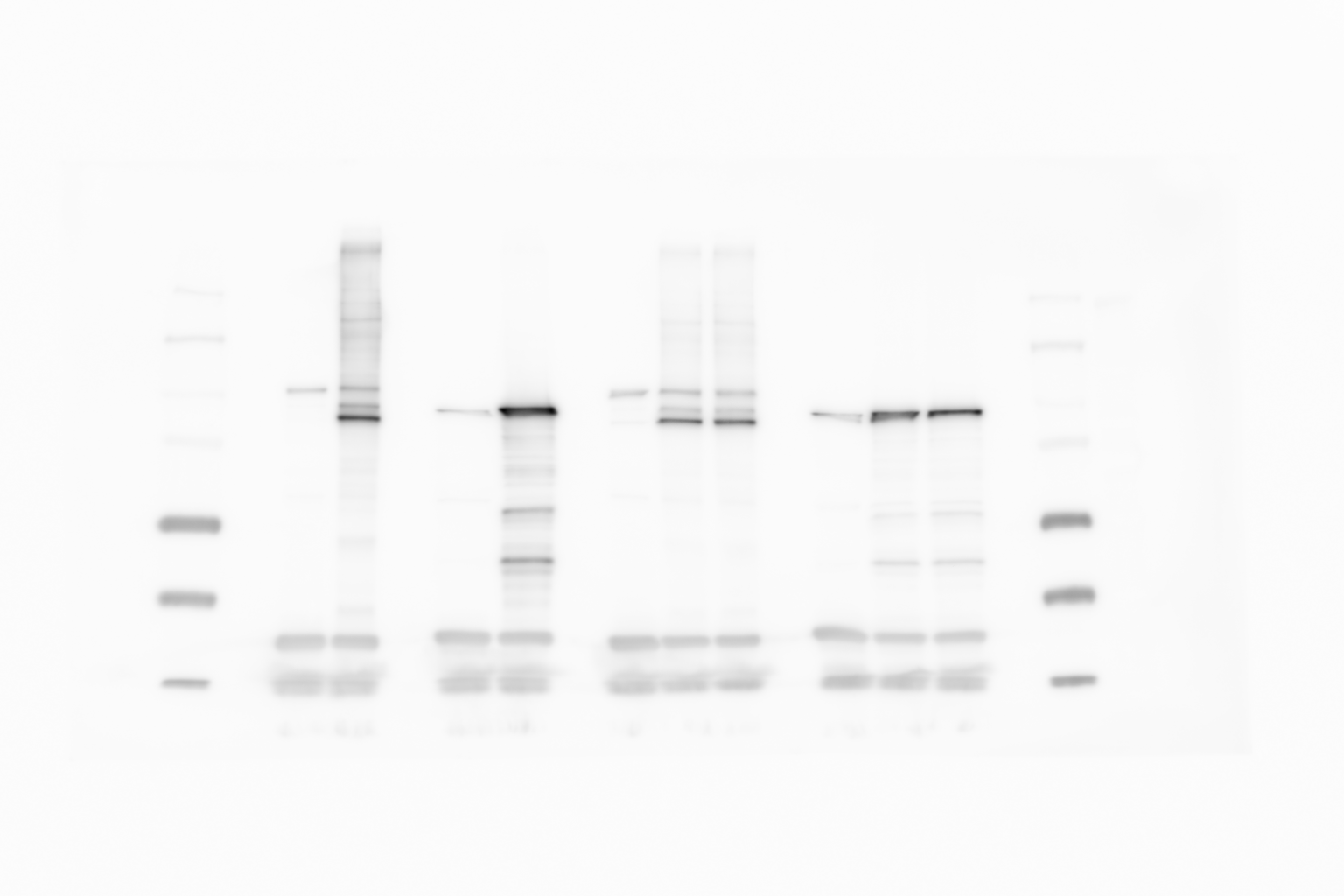

Supplement: Figure 2—figure supplement 1—source data 1. [file elife-70541-fig2-figsupp1-data1.zip › Figure 2-figure supplment 1 Source data files/Figure 2-figure supplement 1-Source data (raw data)/Figure 2-figure supplement 1-Source data 4 (raw data-LptD).tif]

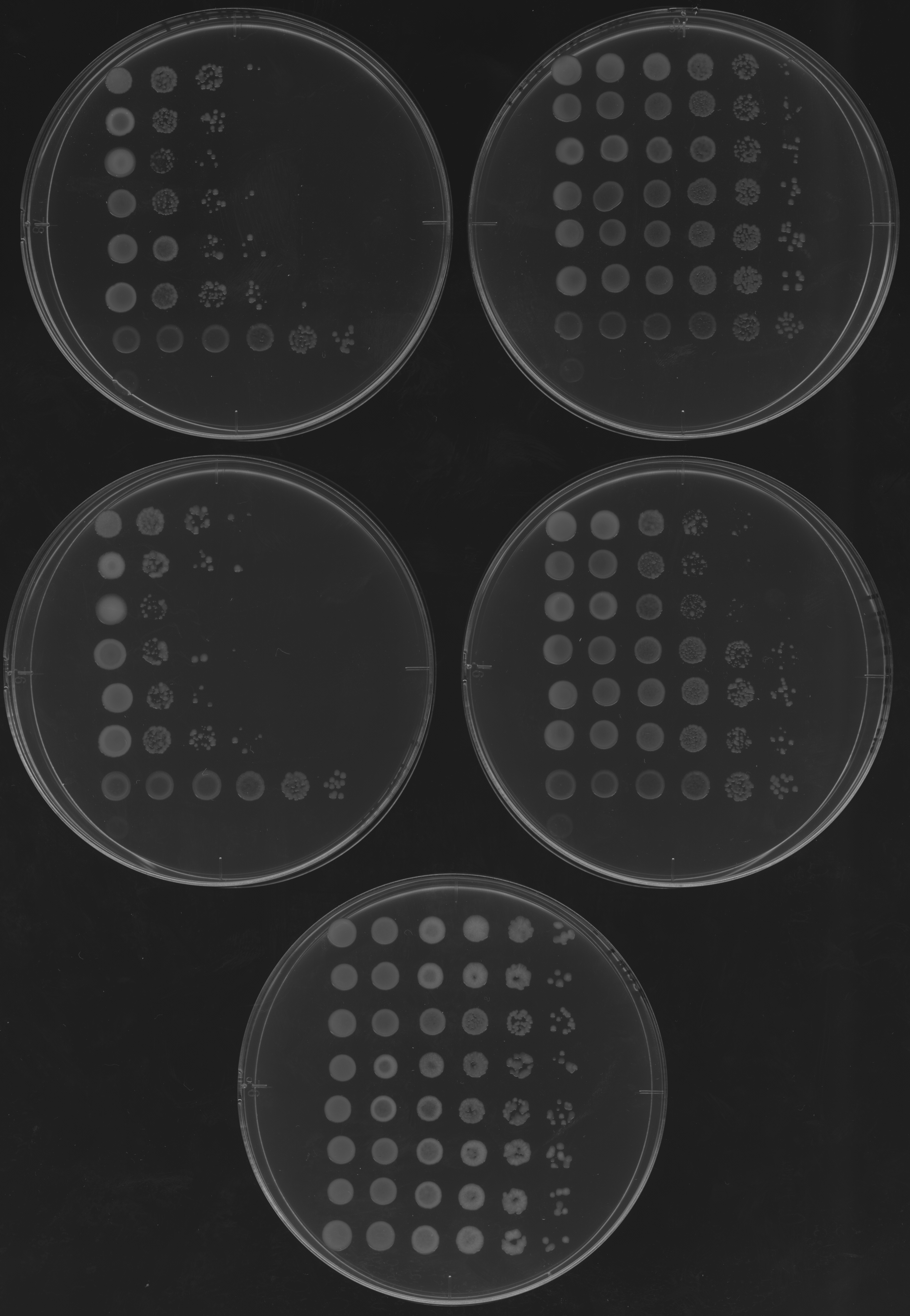

Supplement: Figure 2—figure supplement 2—source data 1. [file elife-70541-fig2-figsupp2-data1.zip › Figure 2-figure supplment 2 Source data files/Figure 2-figure supplement 2-Source data (raw data)/Figure 2-figure supplement 2-Source data 2 (raw data).jpg]

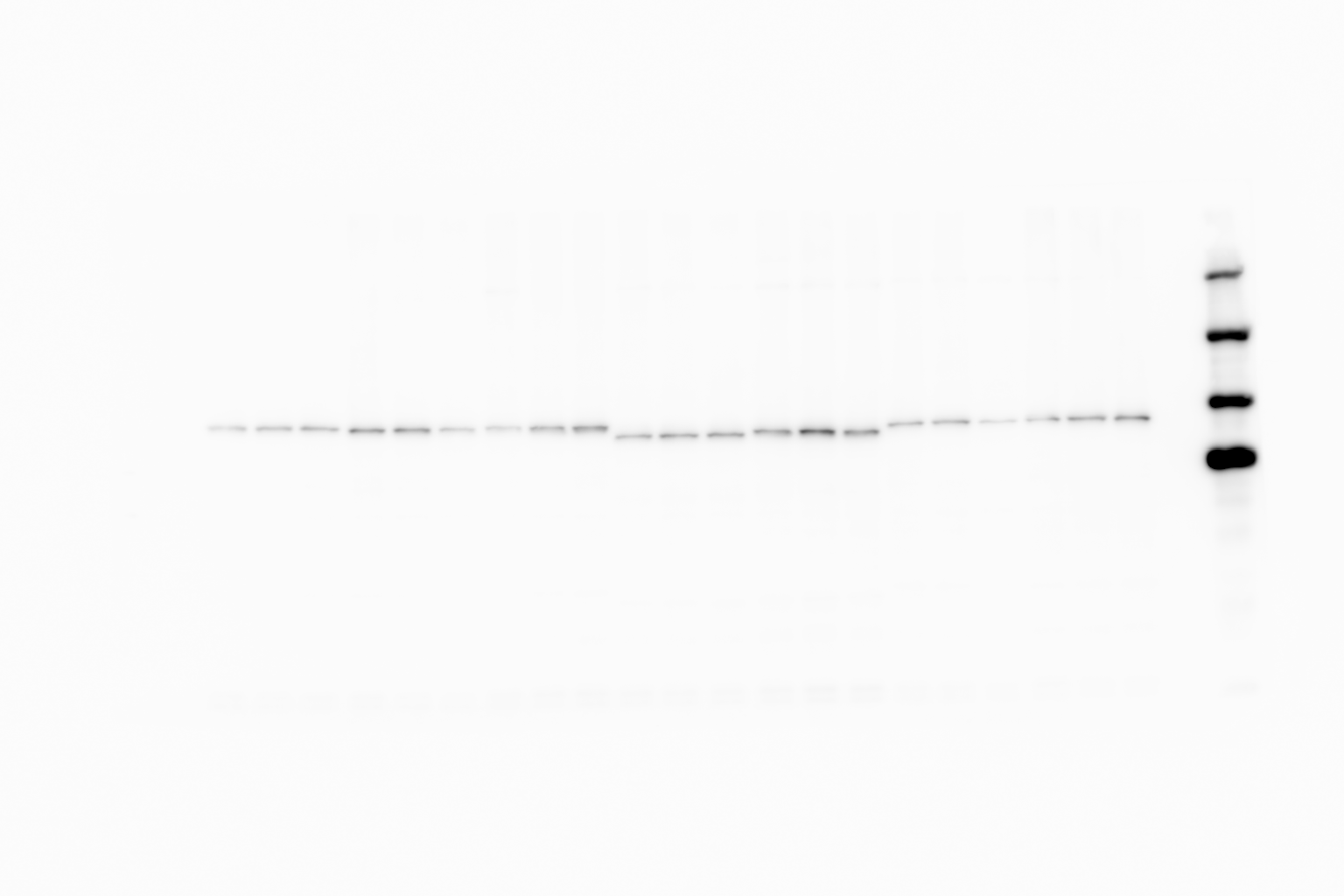

Supplement: Figure 3—source data 1. [file elife-70541-fig3-data1.zip › Figure 3 Source data files/Figure 3-Source data (raw data)/Figure 3-Source data 3 (A_raw data-His).tif]

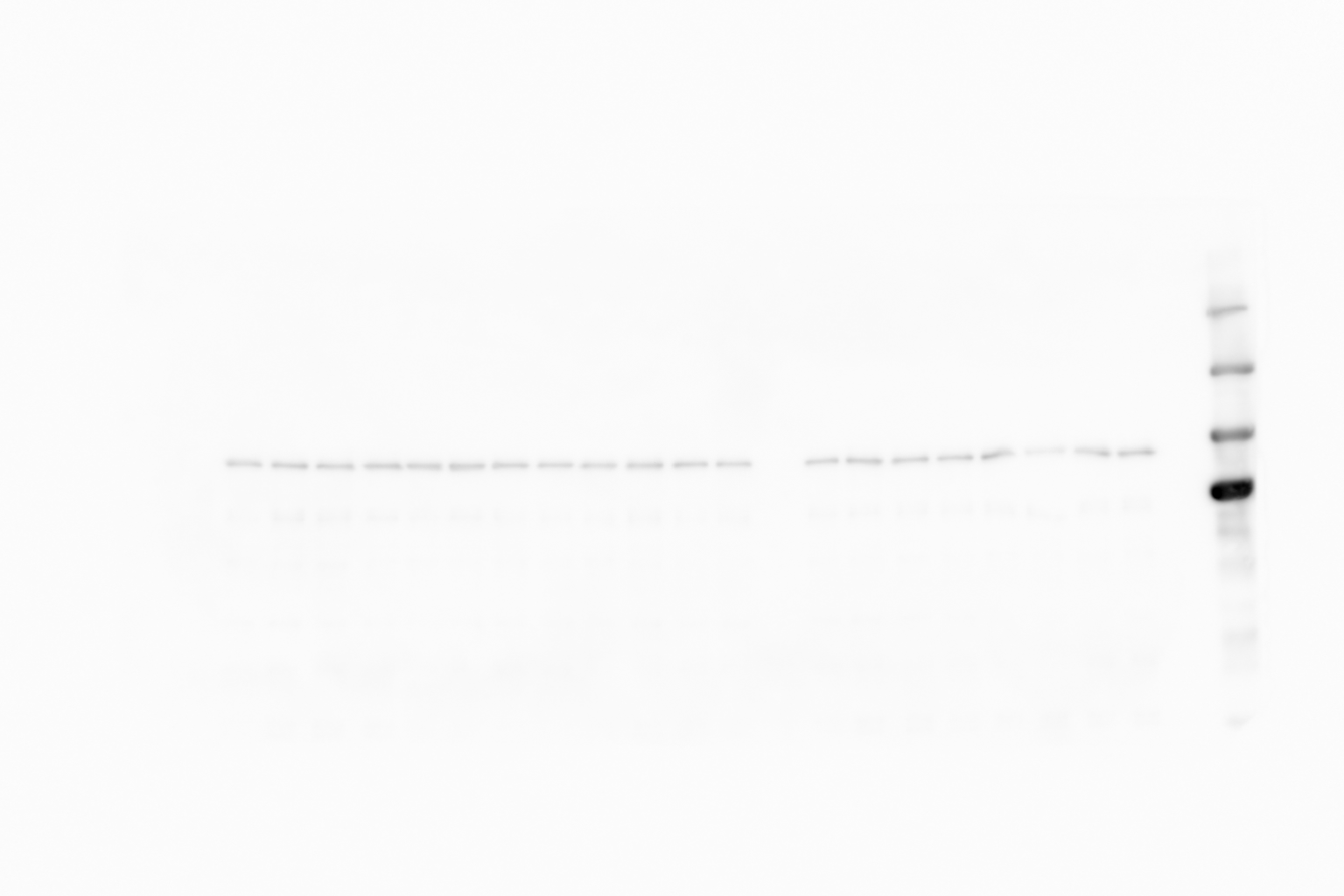

Supplement: Figure 3—source data 1. [file elife-70541-fig3-data1.zip › Figure 3 Source data files/Figure 3-Source data (raw data)/Figure 3-Source data 6 (B_raw data-His +ME).tif]

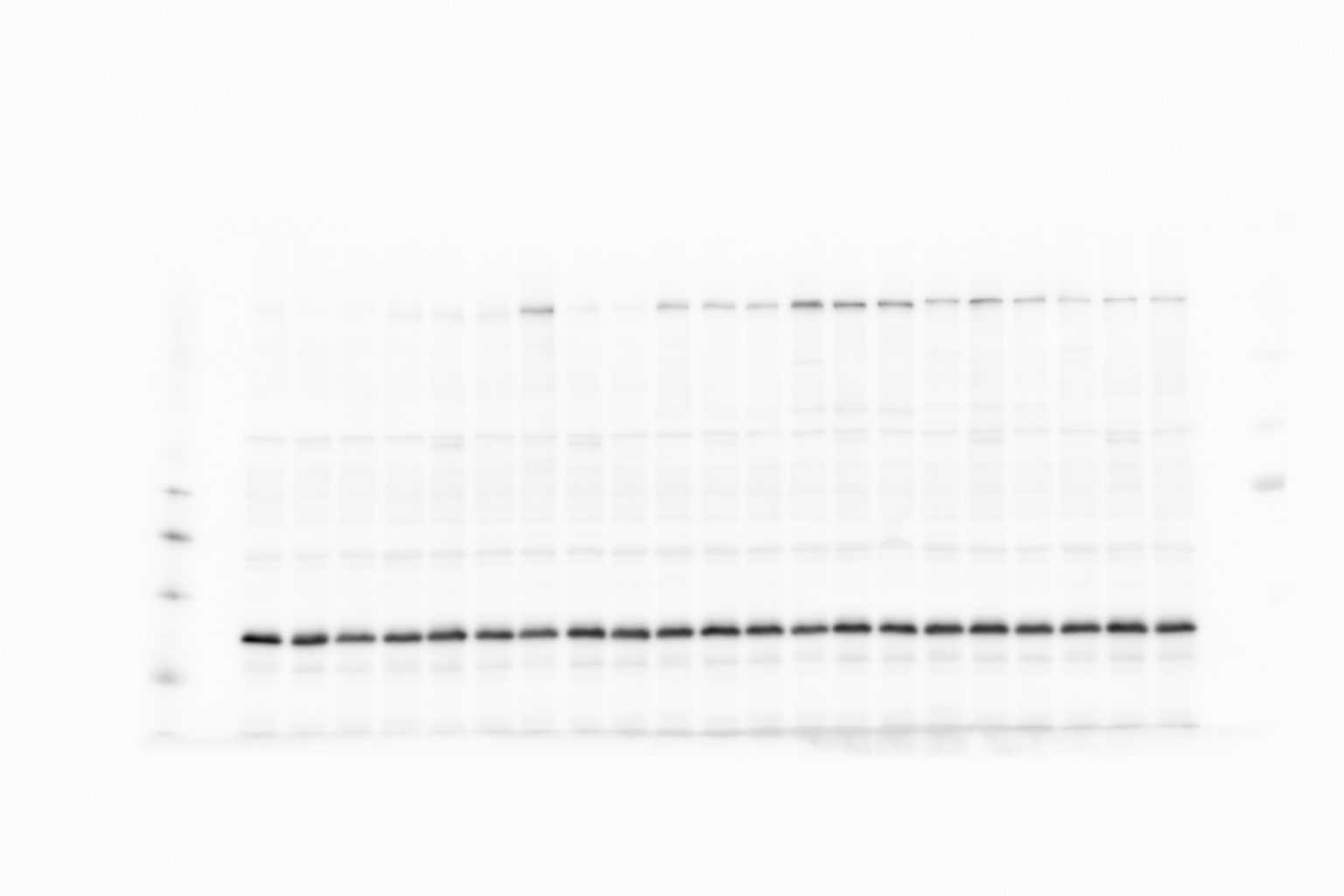

Supplement: Figure 3—source data 1. [file elife-70541-fig3-data1.zip › Figure 3 Source data files/Figure 3-Source data (raw data)/Figure 3-Source data 2 (A_raw data-BepA).tif]

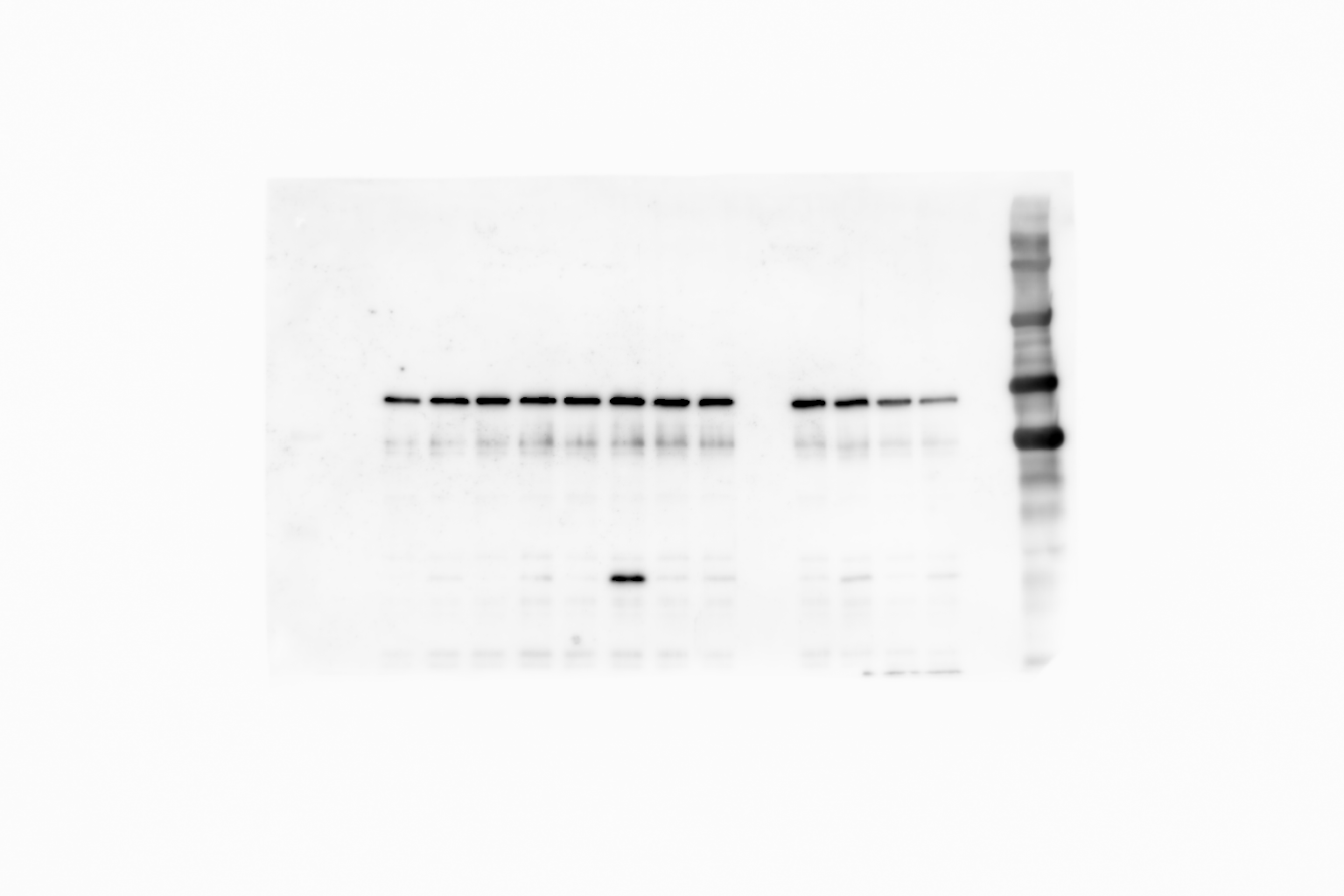

Supplement: Figure 3—source data 1. [file elife-70541-fig3-data1.zip › Figure 3 Source data files/Figure 3-Source data (raw data)/Figure 3-Source data 4 (B_raw data-BepA +ME).tif]

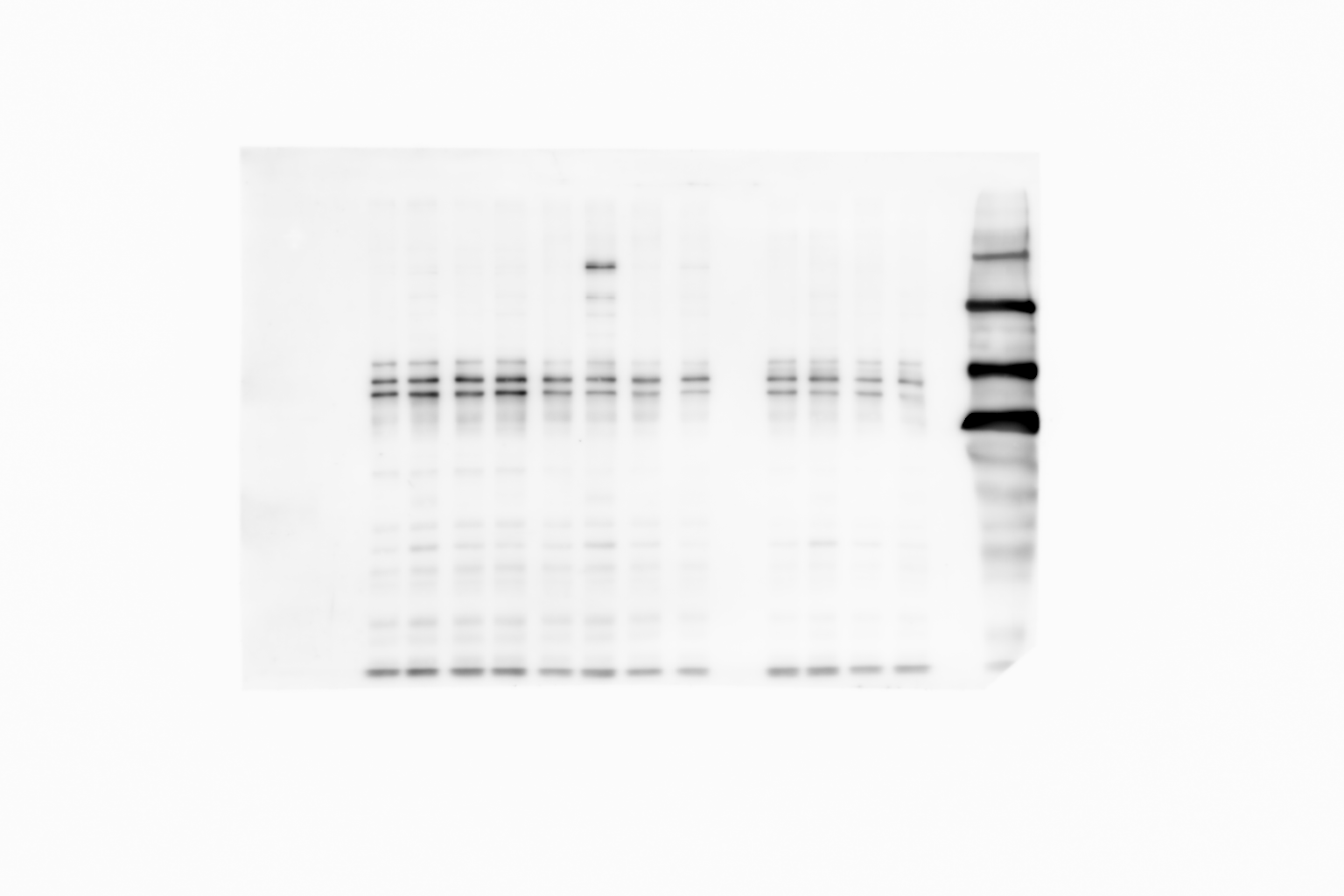

Supplement: Figure 3—source data 1. [file elife-70541-fig3-data1.zip › Figure 3 Source data files/Figure 3-Source data (raw data)/Figure 3-Source data 5 (B_raw data-BepA noME).tif]

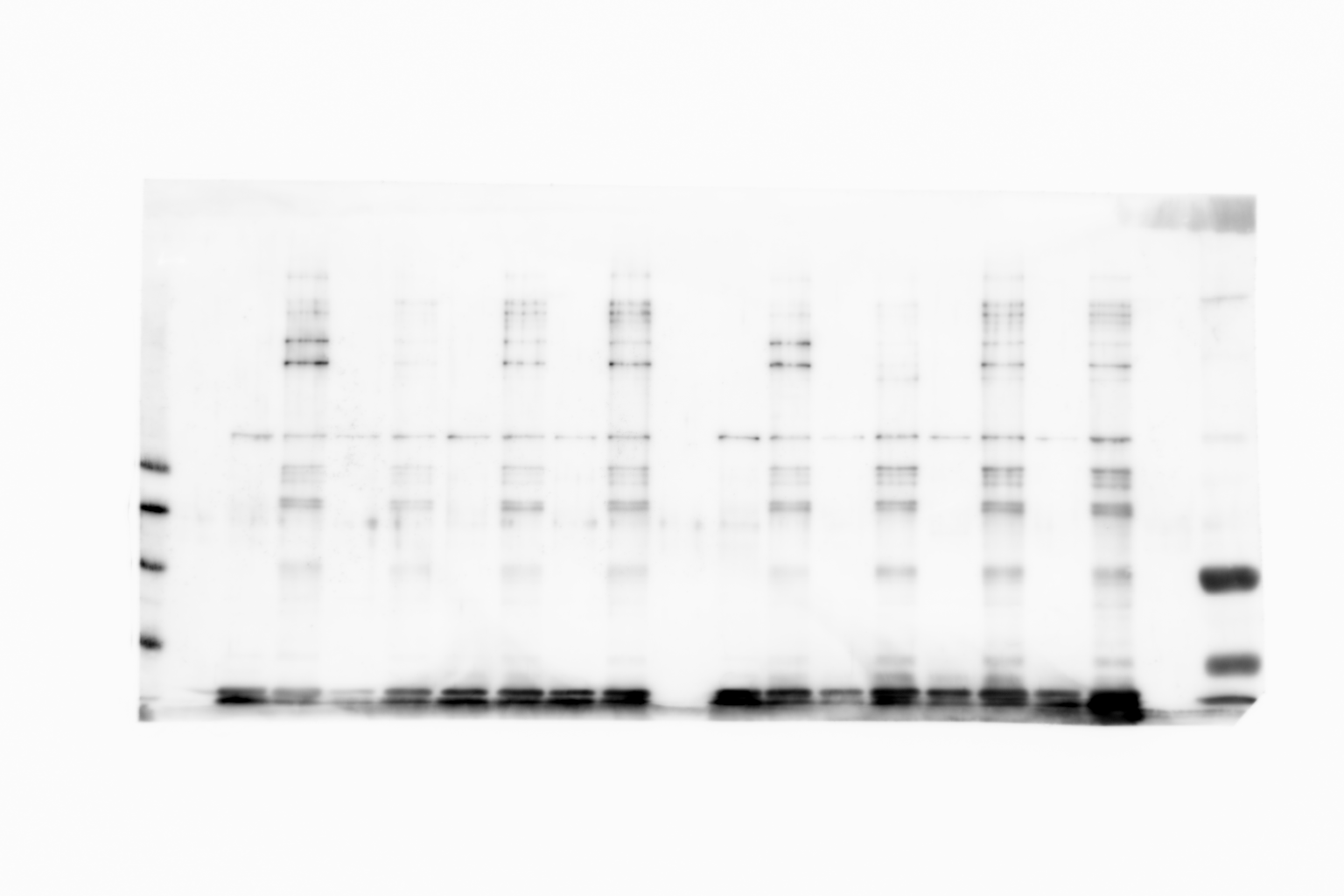

Supplement: Figure 3—figure supplement 1—source data 1. [file elife-70541-fig3-figsupp1-data1.zip › Figure 3-figure supplment 1 Source data files/Figure 3-figure supplement 1-Source data (raw data)/Figure 3-figure supplement 1-Source data 3 (raw data-pure A106-F107-LptD).tif]

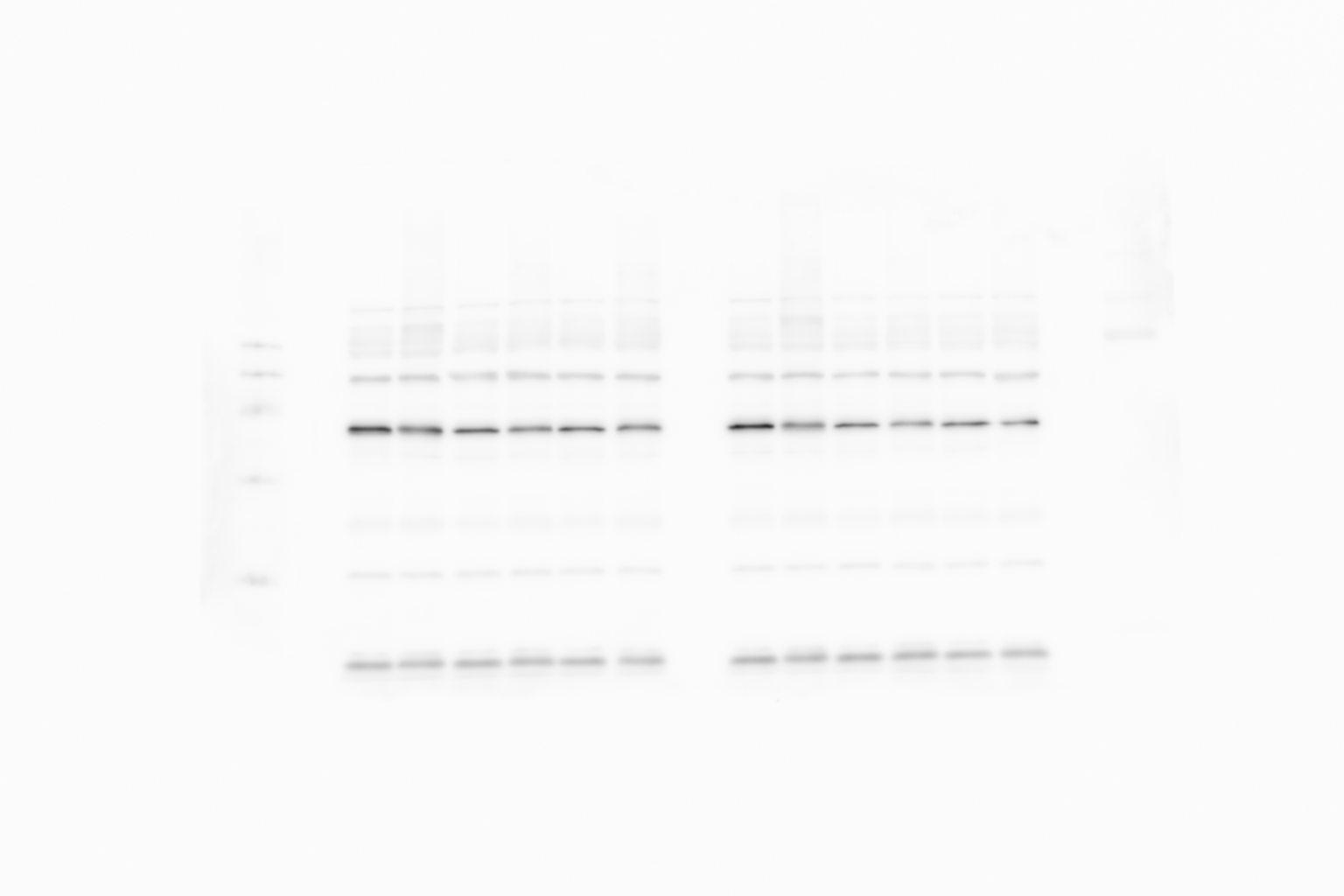

Supplement: Figure 3—figure supplement 1—source data 1. [file elife-70541-fig3-figsupp1-data1.zip › Figure 3-figure supplment 1 Source data files/Figure 3-figure supplement 1-Source data (raw data)/Figure 3-figure supplement 1-Source data 7 (raw data-whole-N105-BepA).tif]

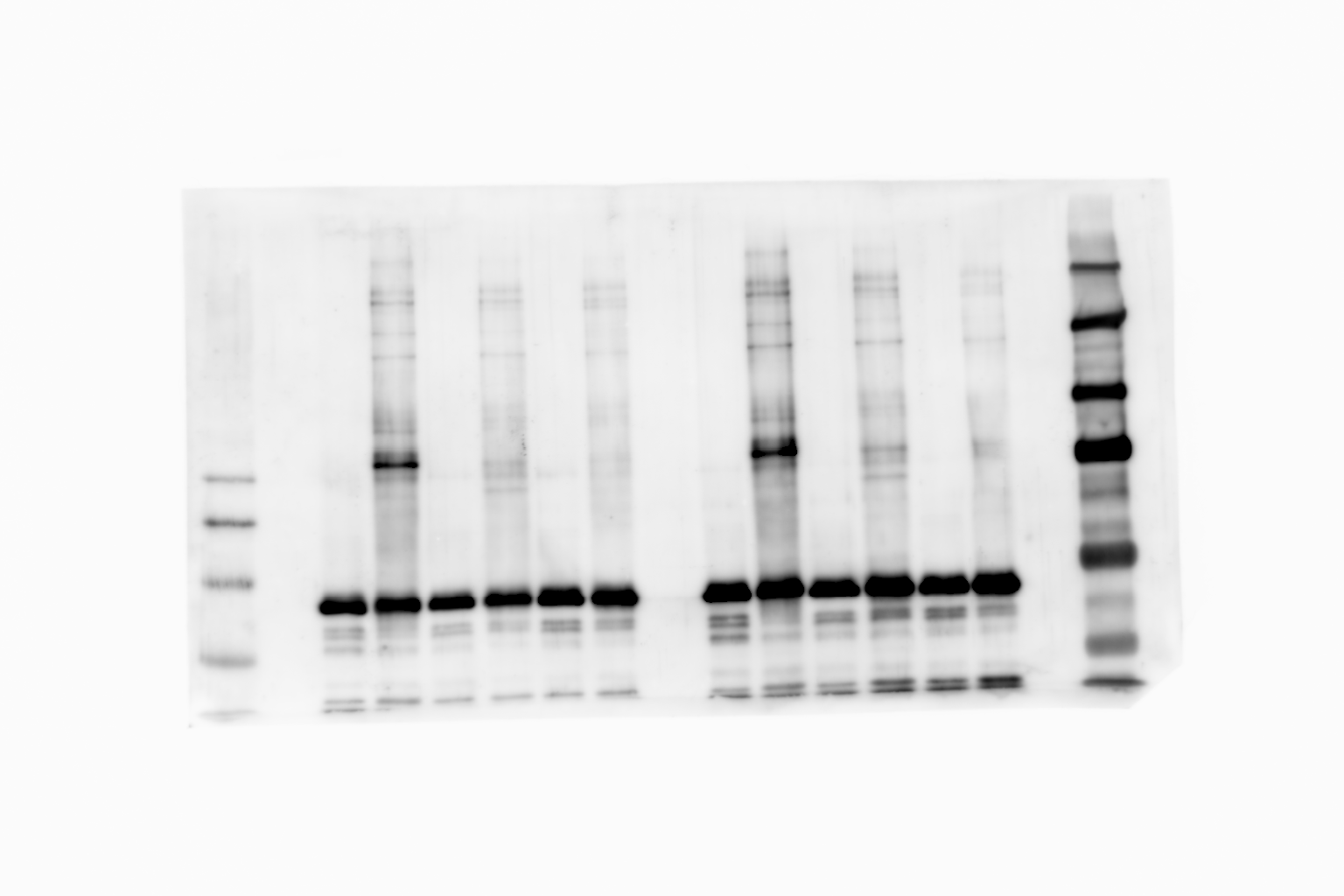

Supplement: Figure 3—figure supplement 1—source data 1. [file elife-70541-fig3-figsupp1-data1.zip › Figure 3-figure supplment 1 Source data files/Figure 3-figure supplement 1-Source data (raw data)/Figure 3-figure supplement 1-Source data 4 (raw data-pure N105-BepA).tif]

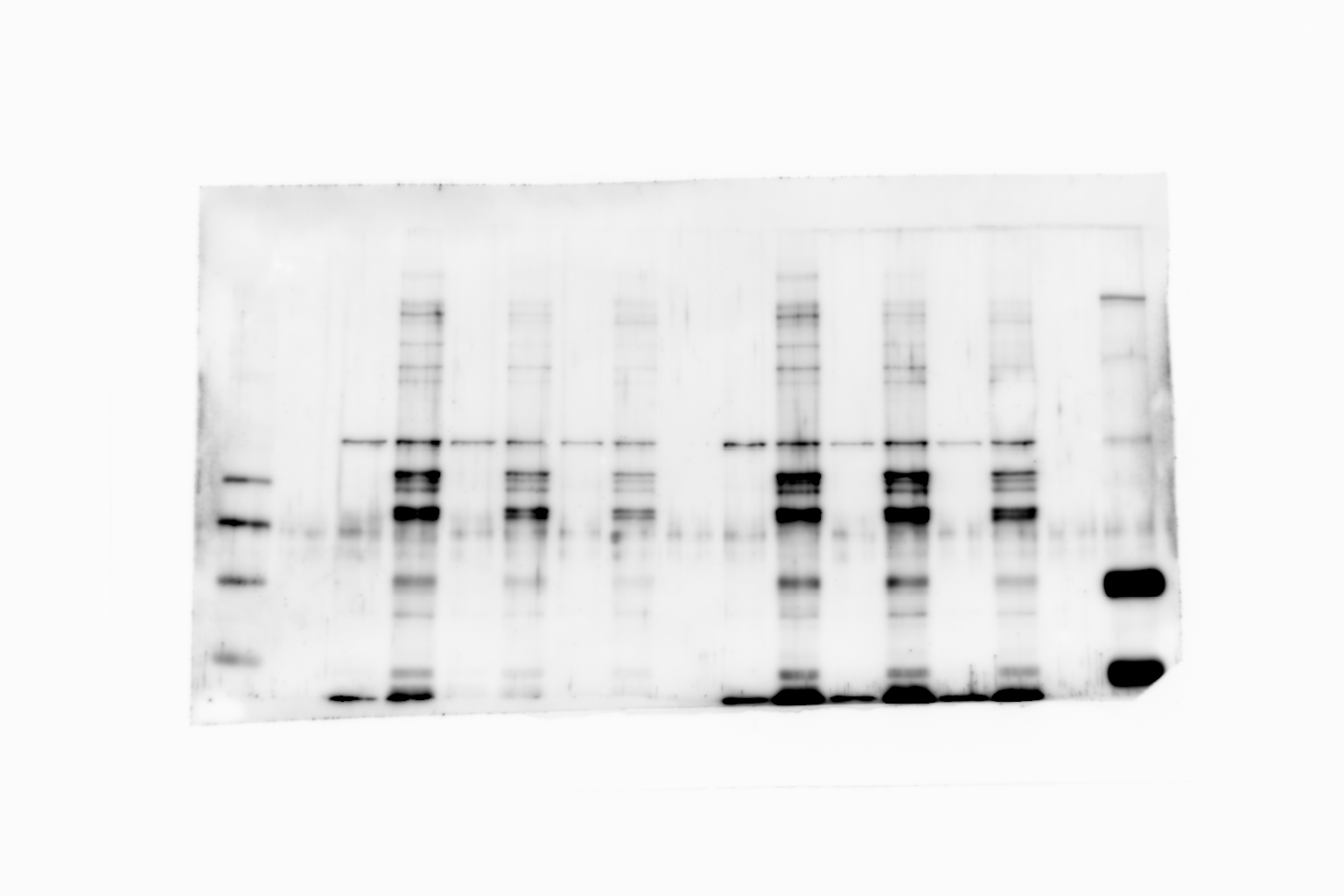

Supplement: Figure 3—figure supplement 1—source data 1. [file elife-70541-fig3-figsupp1-data1.zip › Figure 3-figure supplment 1 Source data files/Figure 3-figure supplement 1-Source data (raw data)/Figure 3-figure supplement 1-Source data 5 (raw data-pure N105-LptD).tif]

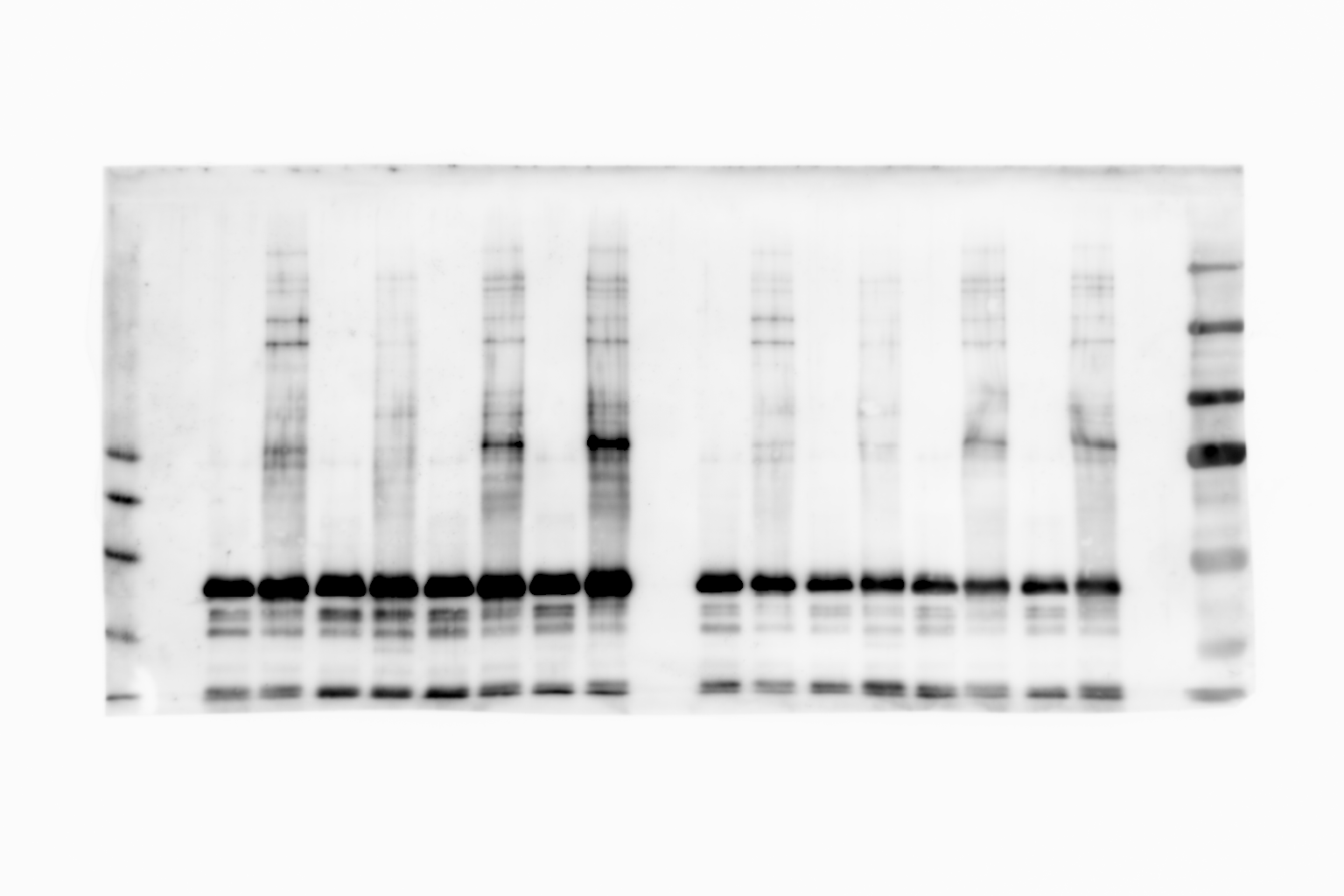

Supplement: Figure 3—figure supplement 1—source data 1. [file elife-70541-fig3-figsupp1-data1.zip › Figure 3-figure supplment 1 Source data files/Figure 3-figure supplement 1-Source data (raw data)/Figure 3-figure supplement 1-Source data 2 (raw data-pure A106-F107-BepA).tif]

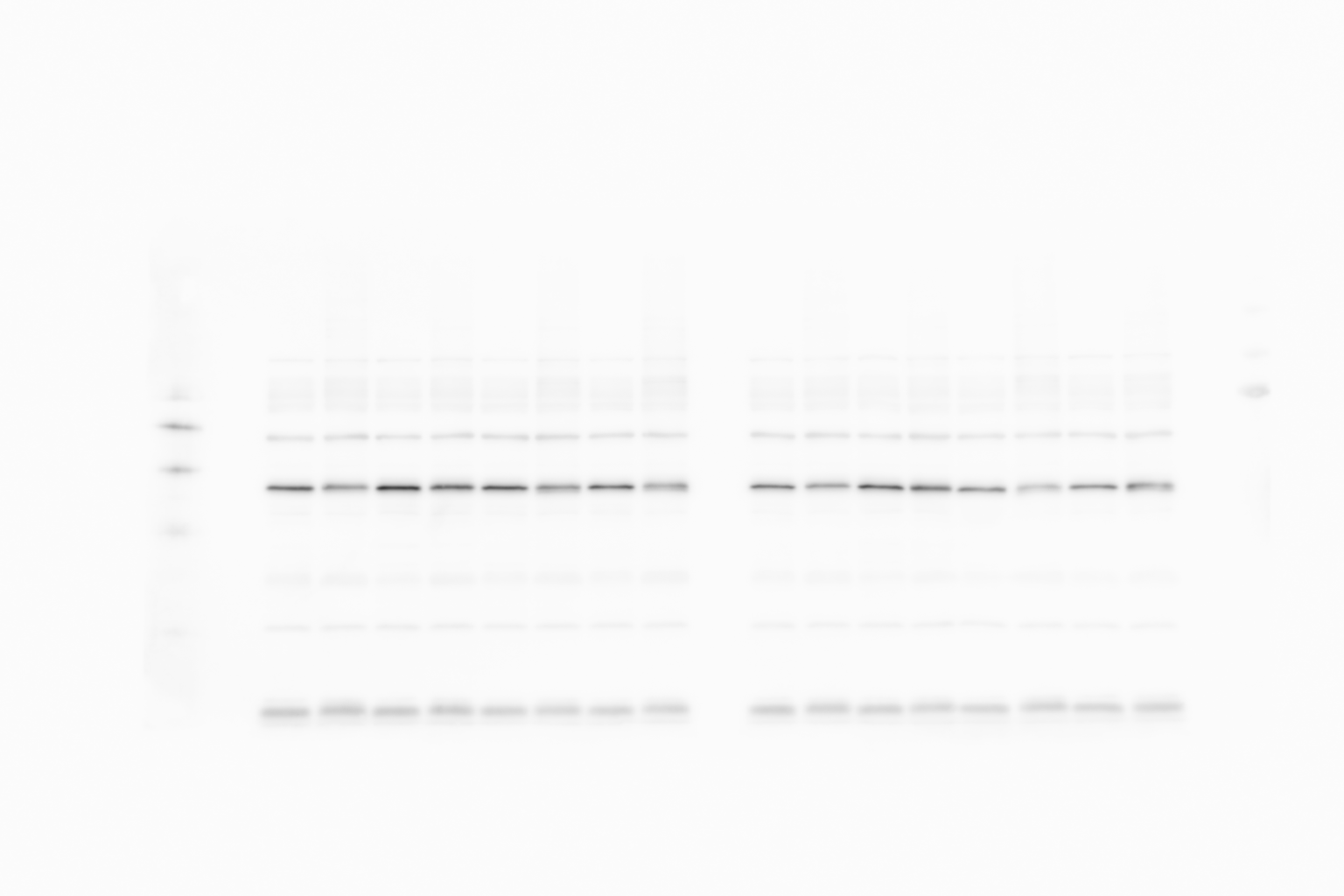

Supplement: Figure 3—figure supplement 1—source data 1. [file elife-70541-fig3-figsupp1-data1.zip › Figure 3-figure supplment 1 Source data files/Figure 3-figure supplement 1-Source data (raw data)/Figure 3-figure supplement 1-Source data 6 (raw data-whole-A106-F107-BepA).tif]

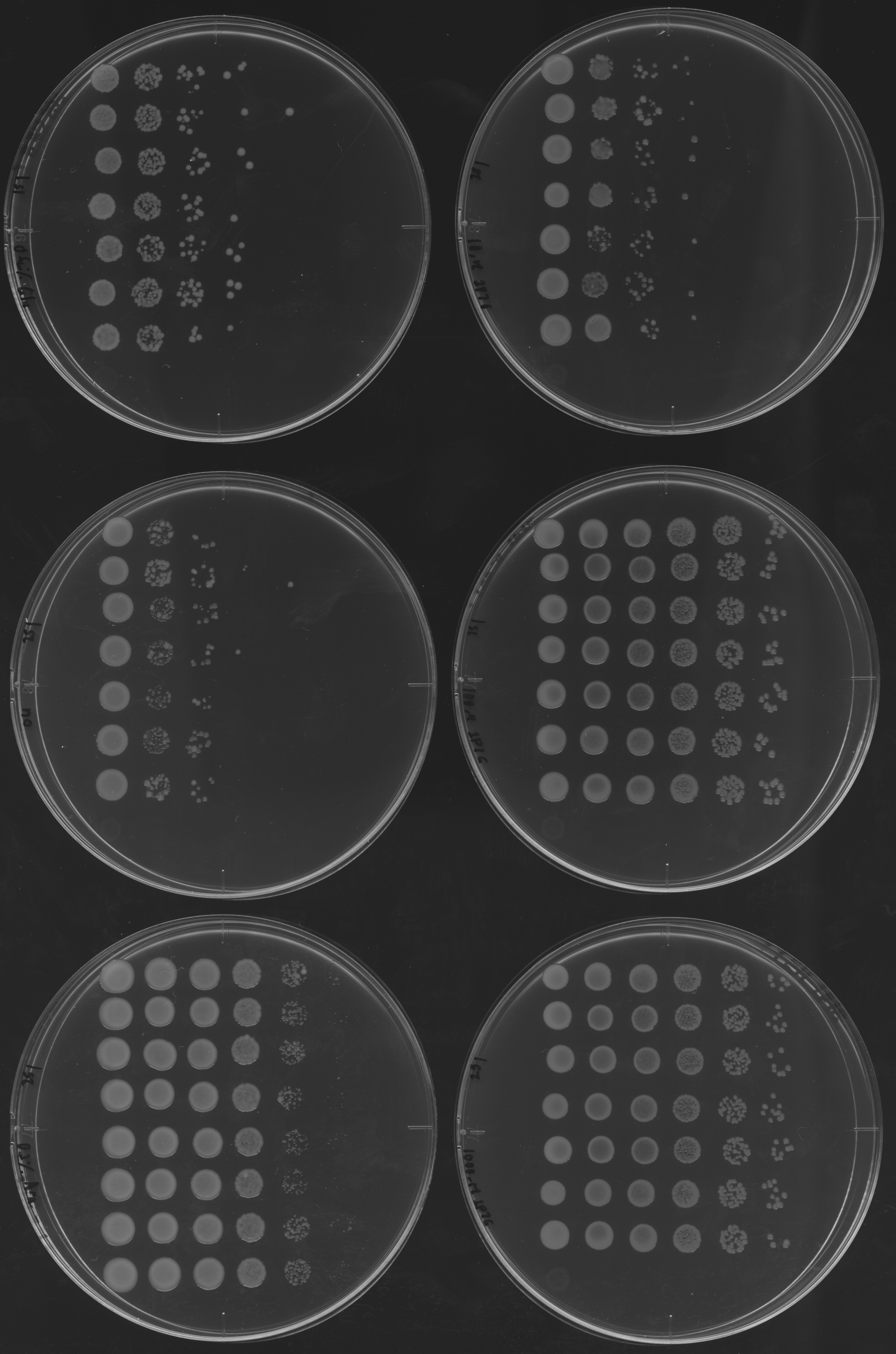

Supplement: Figure 3—figure supplement 2—source data 1. [file elife-70541-fig3-figsupp2-data1.zip › Figure 3-figure supplment 2 Source data files/Figure 3-figure supplement 2-Source data (raw data) /Figure 3-figure supplement 2-Source data 2 (A_raw data).jpg]

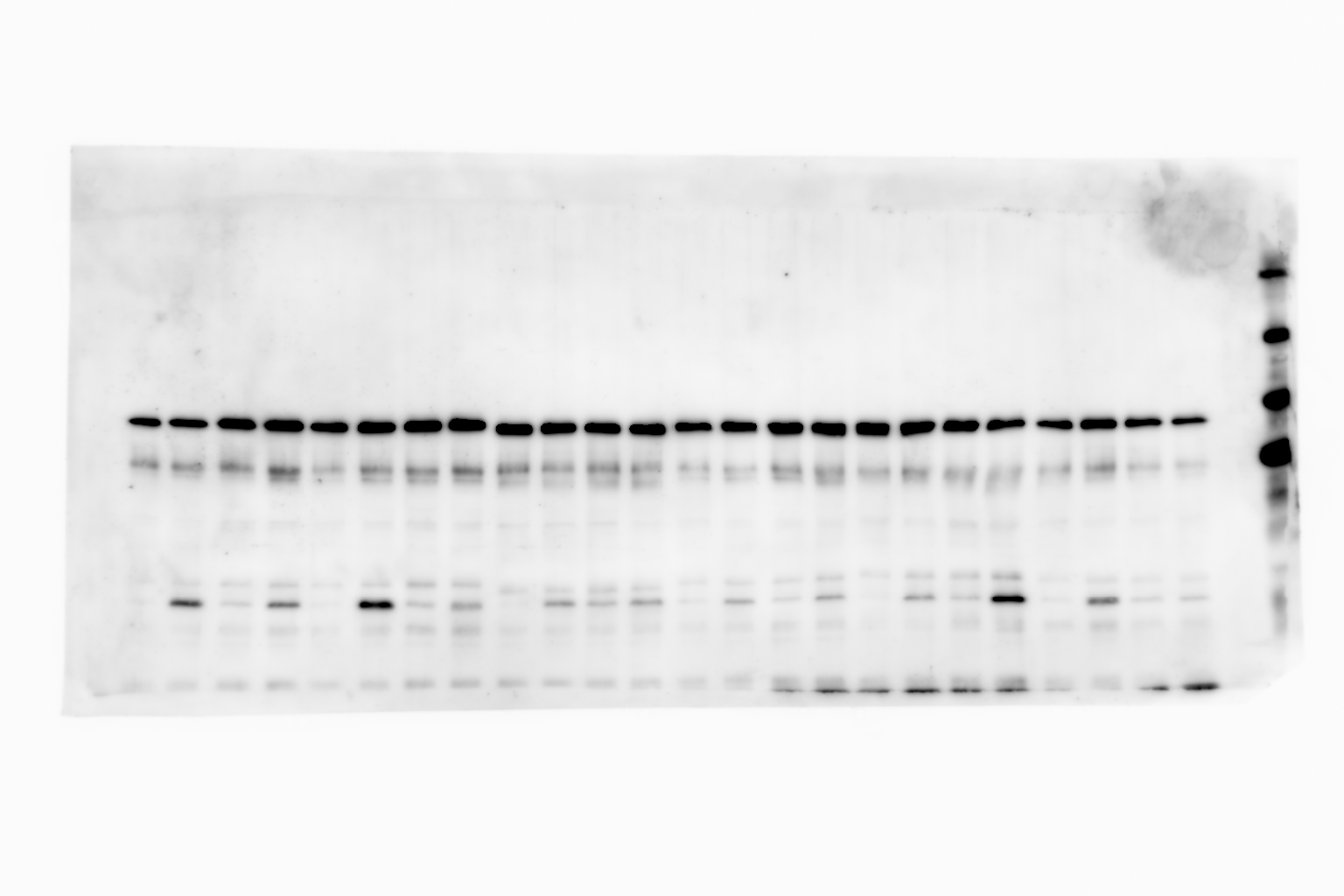

Supplement: Figure 3—figure supplement 2—source data 1. [file elife-70541-fig3-figsupp2-data1.zip › Figure 3-figure supplment 2 Source data files/Figure 3-figure supplement 2-Source data (raw data) /Figure 3-figure supplement 2-Source data 3 (B_raw data-BepA +ME).tif]

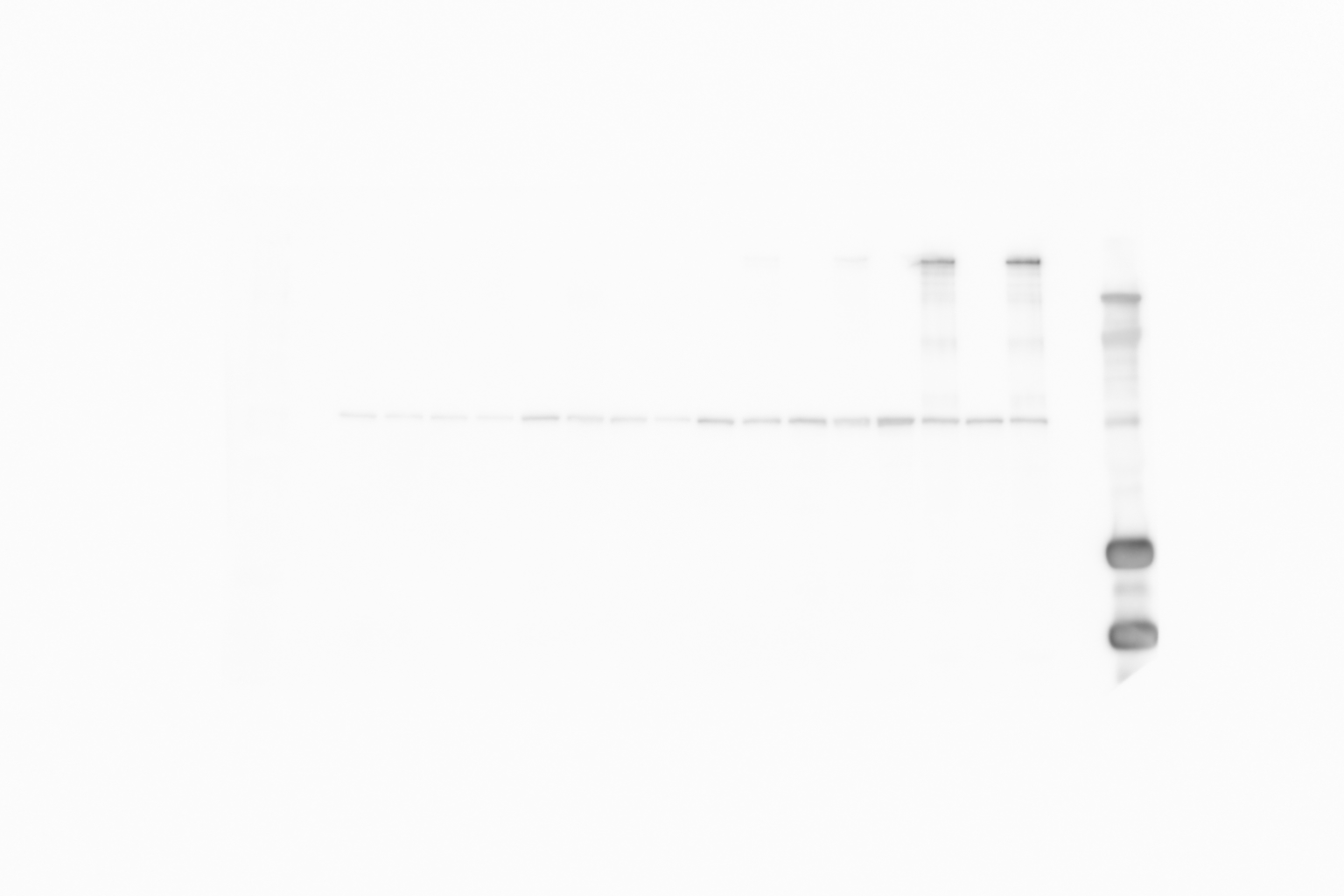

Supplement: Figure 3—figure supplement 2—source data 1. [file elife-70541-fig3-figsupp2-data1.zip › Figure 3-figure supplment 2 Source data files/Figure 3-figure supplement 2-Source data (raw data) /Figure 4-figure supplement 2-Source data 5 (B_raw data-BamA).tif]

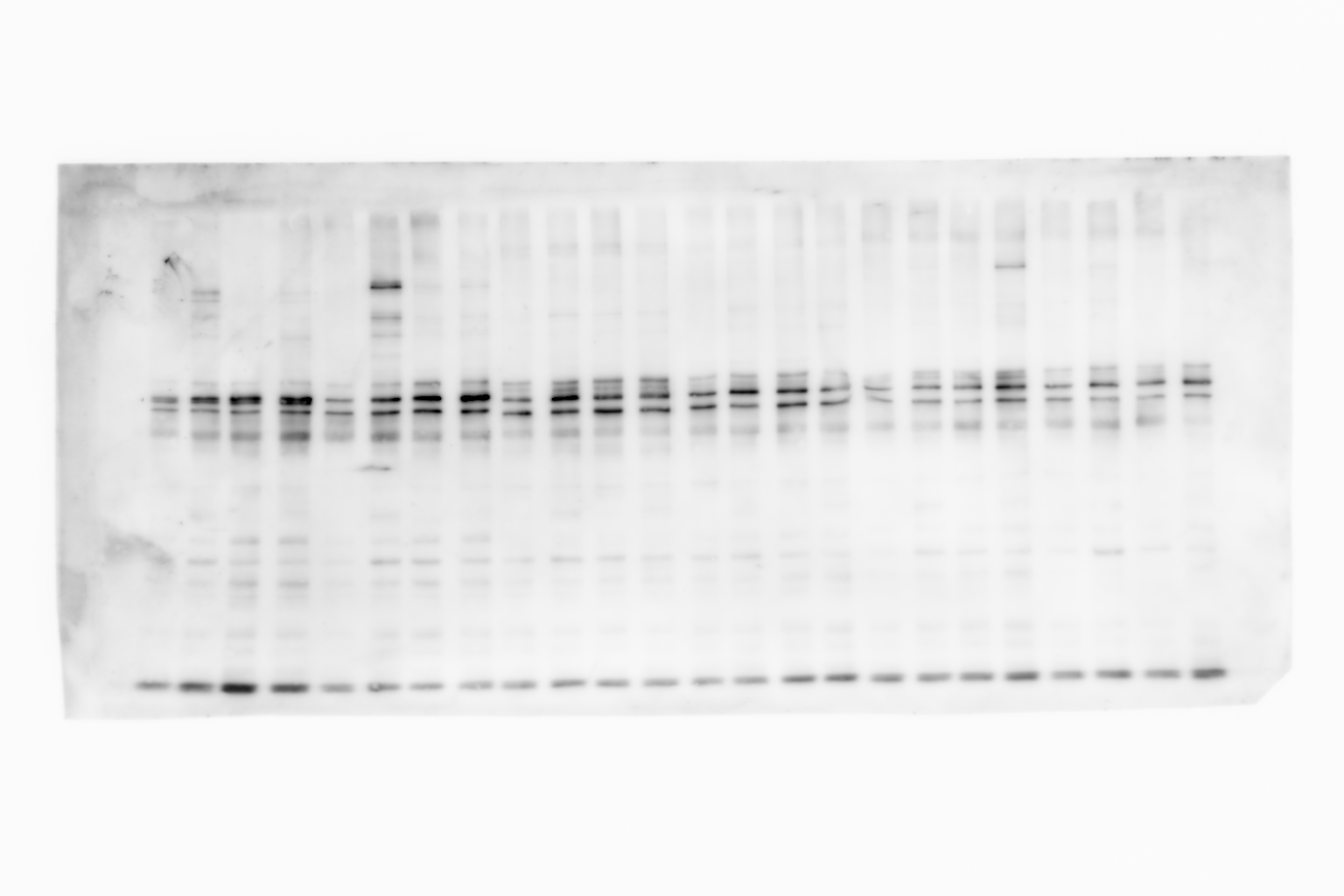

Supplement: Figure 3—figure supplement 2—source data 1. [file elife-70541-fig3-figsupp2-data1.zip › Figure 3-figure supplment 2 Source data files/Figure 3-figure supplement 2-Source data (raw data) /Figure 3-figure supplement 2-Source data 4 (B_raw data-BepA noME).tif]

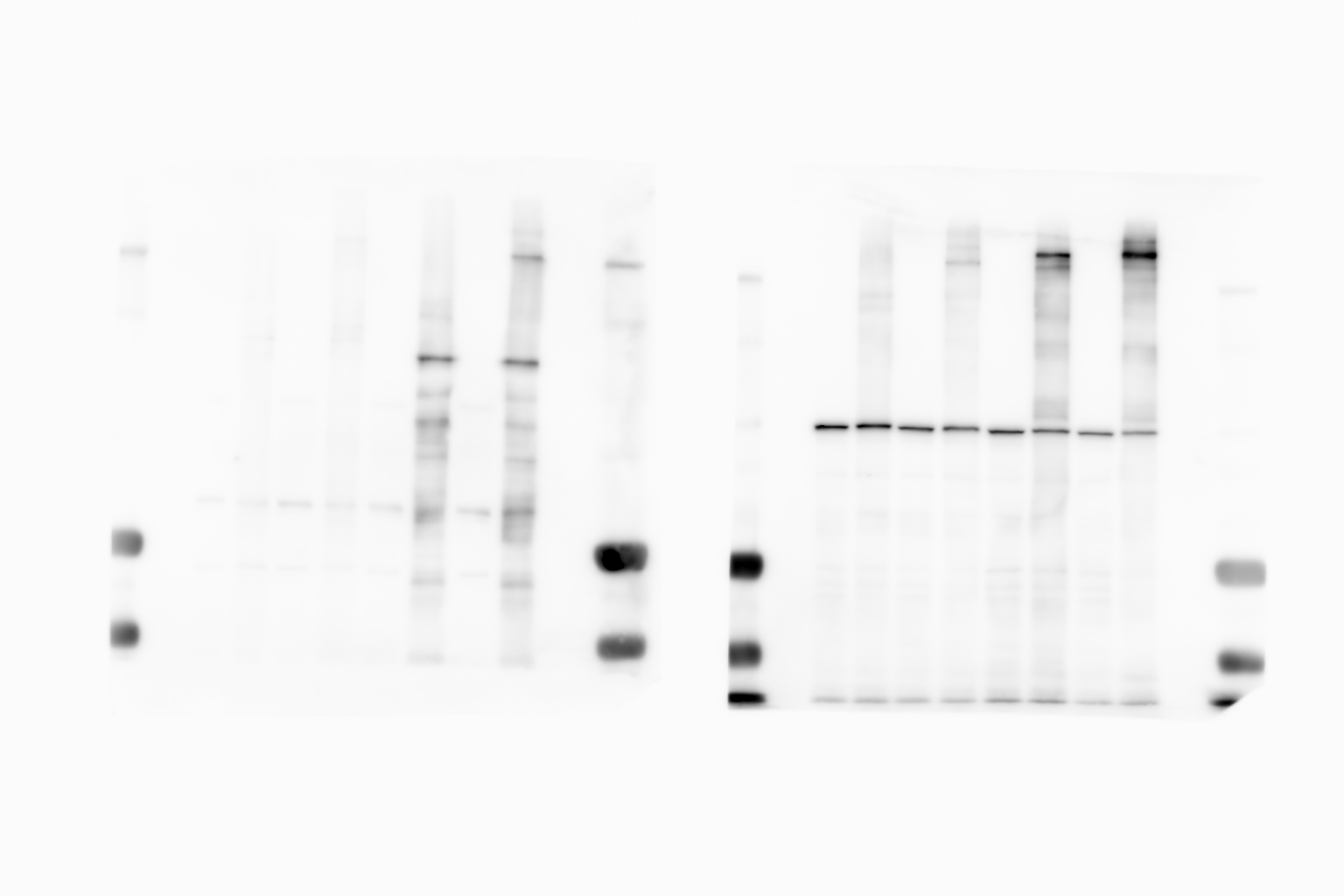

Supplement: Figure 4—source data 1. [file elife-70541-fig4-data1.zip › Figure 4 Source data files/Figure 4-Source data (raw data)/Figure 4-Source data 4 (B_raw data-BamD).tif]

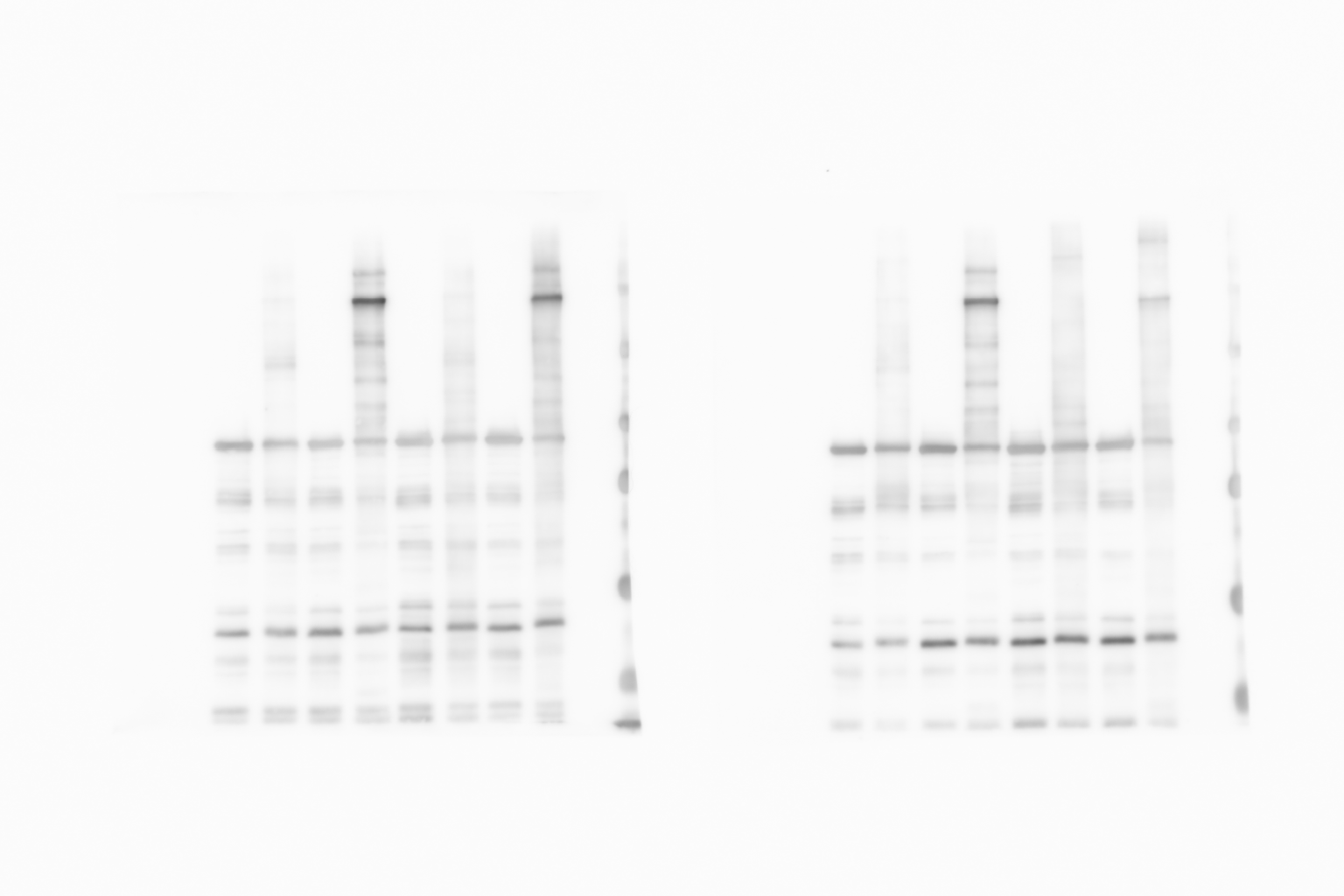

Supplement: Figure 4—source data 1. [file elife-70541-fig4-data1.zip › Figure 4 Source data files/Figure 4-Source data (raw data)/Figure 4-Source data 3 (A_raw data-BepA).tif]

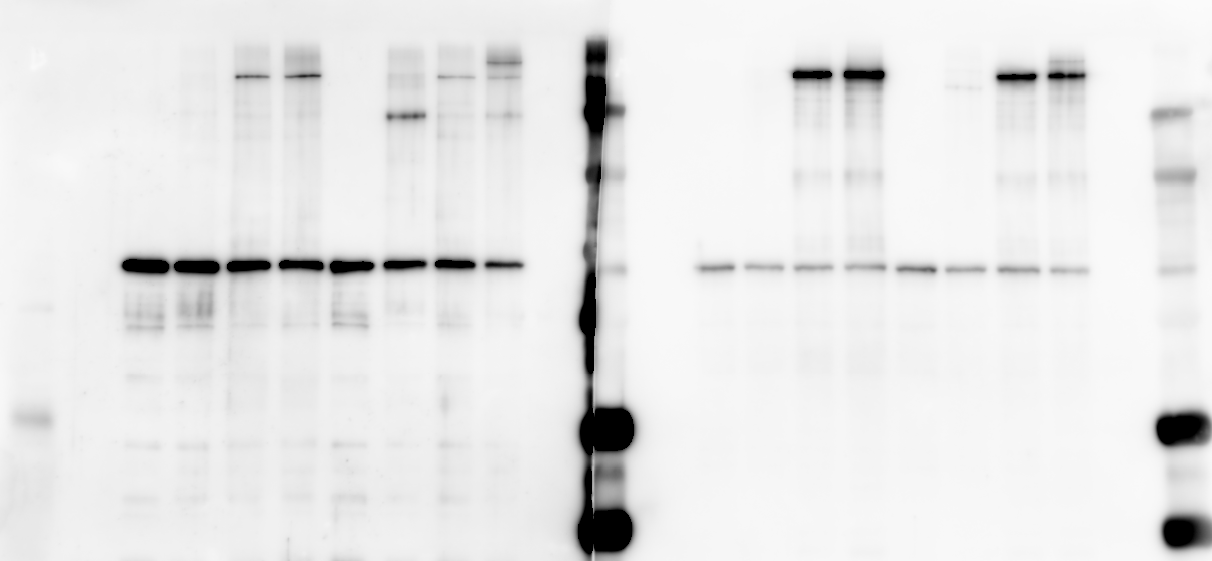

Supplement: Figure 4—source data 1. [file elife-70541-fig4-data1.zip › Figure 4 Source data files/Figure 4-Source data (raw data)/Figure 4-Source data 6 (C_raw data-BepA and BamA).tif]

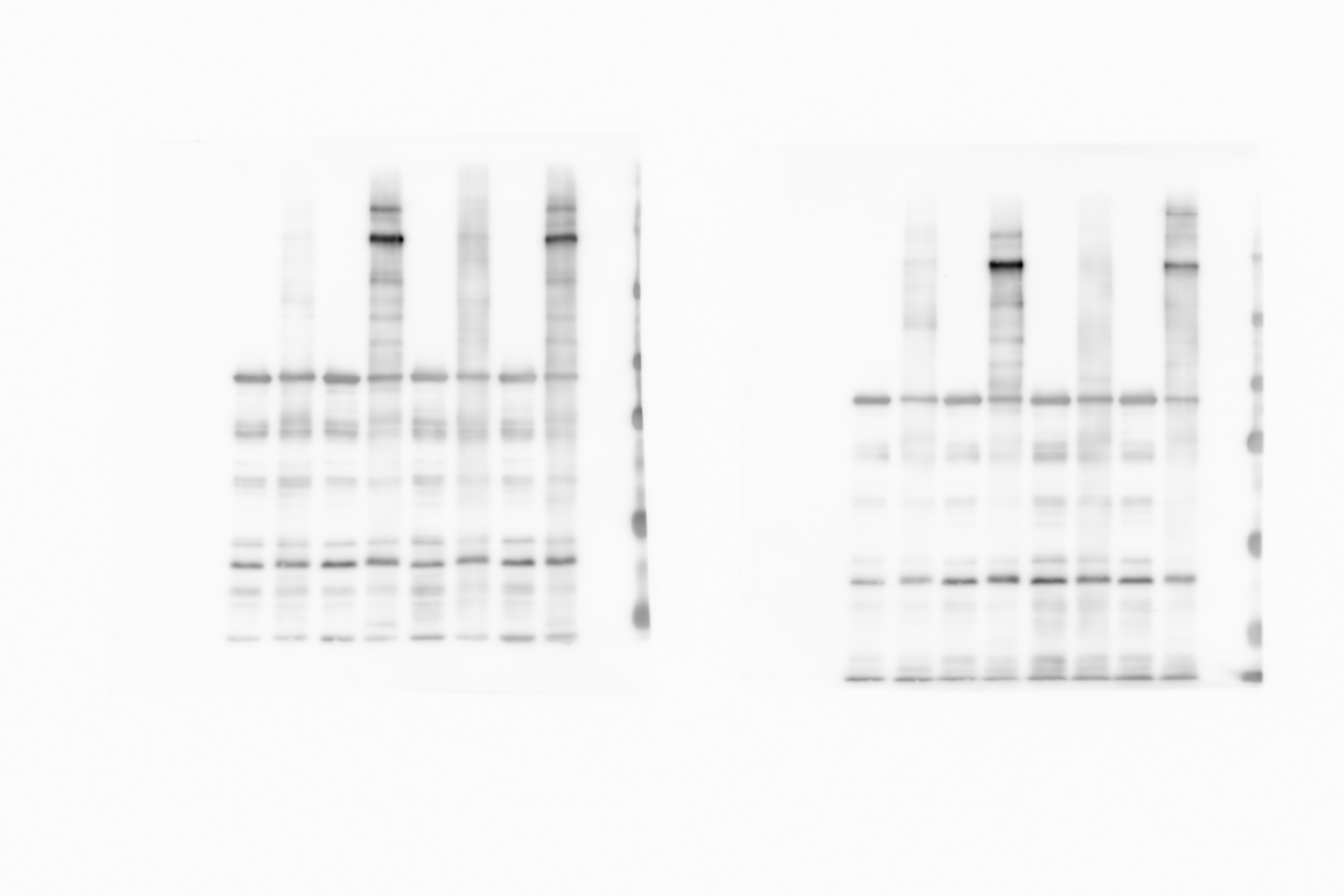

Supplement: Figure 4—source data 1. [file elife-70541-fig4-data1.zip › Figure 4 Source data files/Figure 4-Source data (raw data)/Figure 4-Source data 5 (B_raw data-BepA).tif]

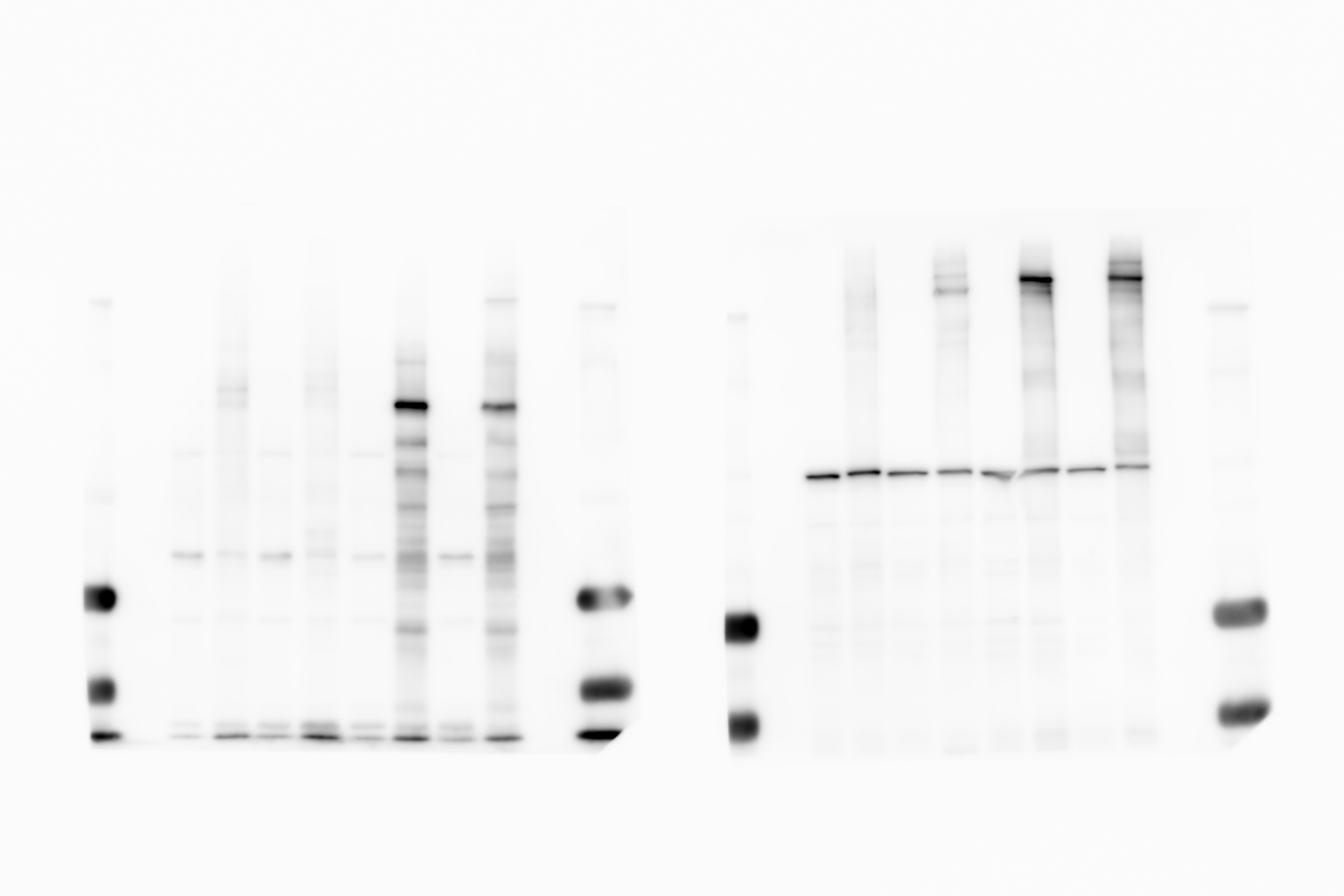

Supplement: Figure 4—source data 1. [file elife-70541-fig4-data1.zip › Figure 4 Source data files/Figure 4-Source data (raw data)/Figure 4-Source data 2 (A_raw data-BamA).tif]

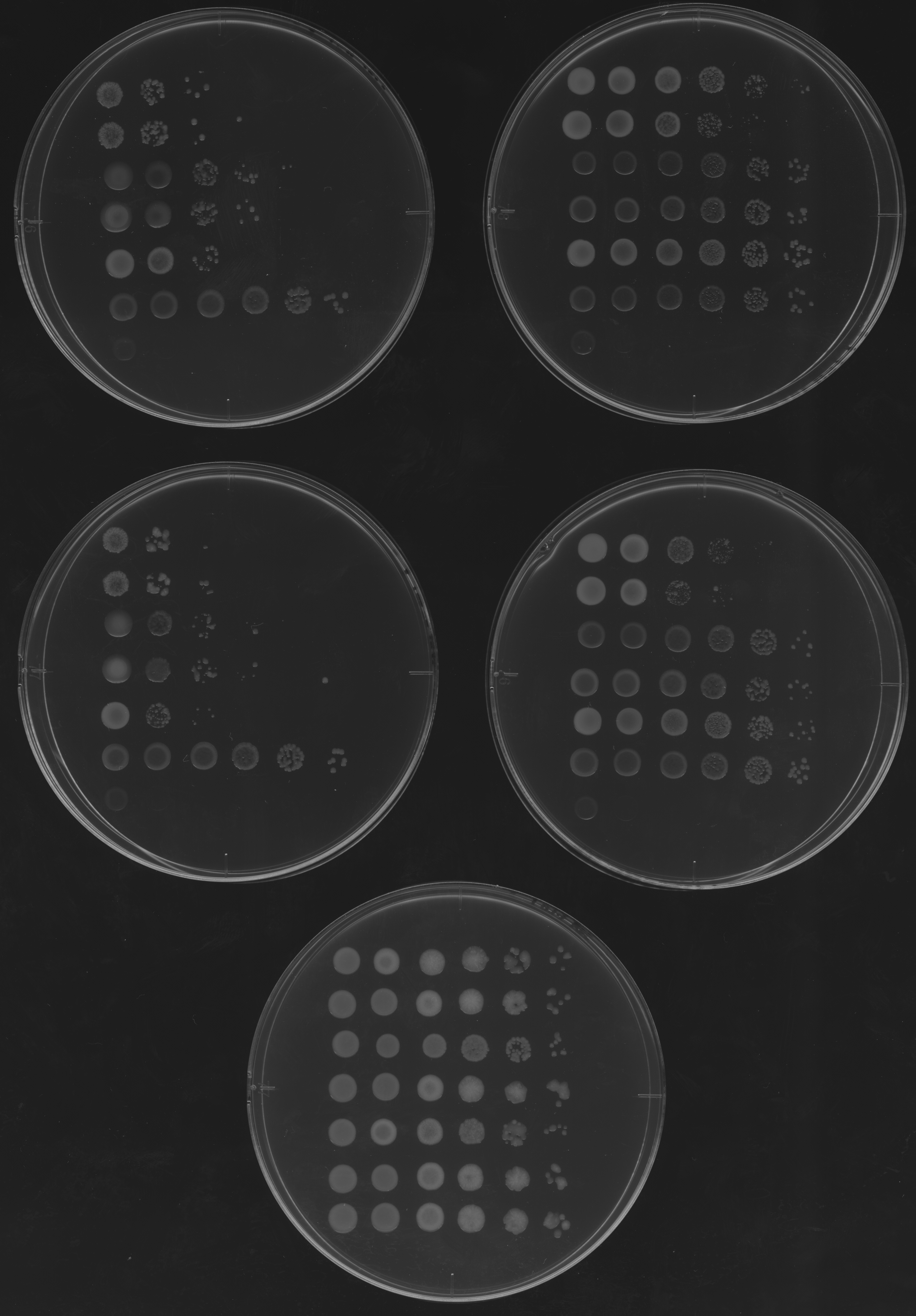

Supplement: Figure 4—figure supplement 1—source data 1. [file elife-70541-fig4-figsupp1-data1.zip › Figure 4-figure supplment 1 Source data files/Figure 4-figure supplement 1-Source data (raw data)/Figure 4-figure supplement 1-Source data 2 (raw data).jpg]

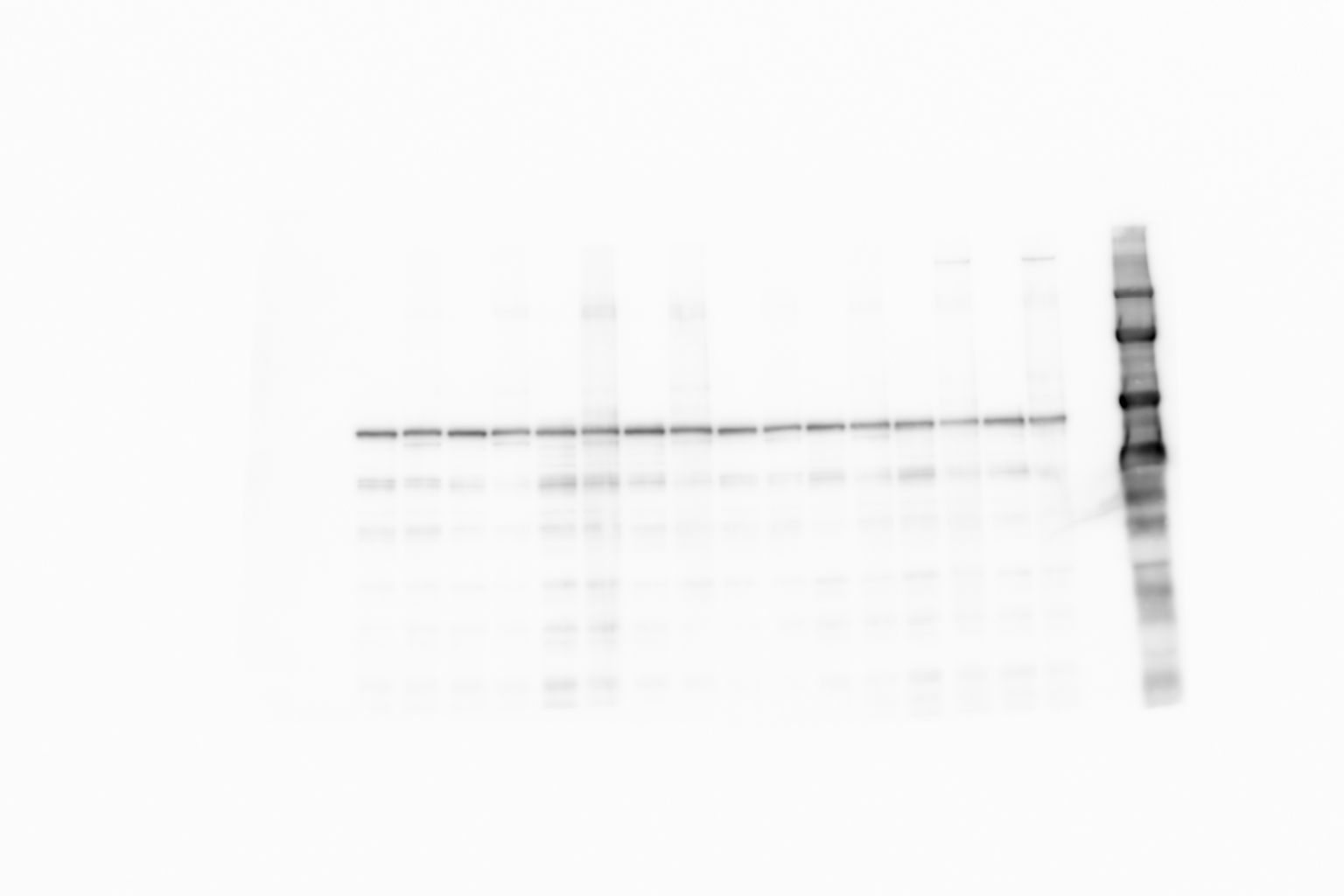

Supplement: Figure 4—figure supplement 2—source data 1. [file elife-70541-fig4-figsupp2-data1.zip › Figure 4-figure supplment 2 Source data files/Figure 4-figure supplement 2-Source data (raw data)/Figure 4-figure supplement 2-Source data 3 (A_raw data-His).tif]

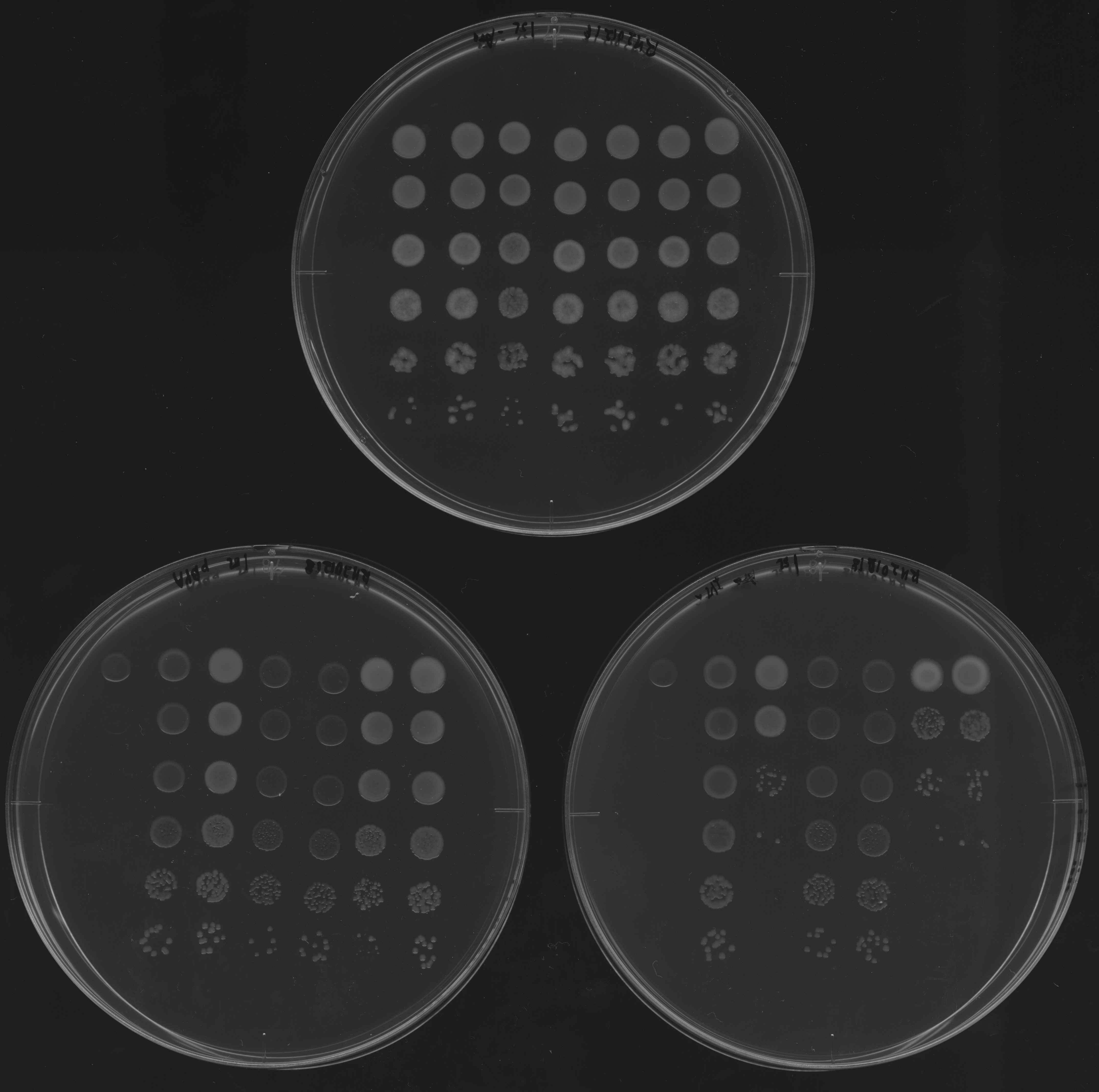

Supplement: Figure 4—figure supplement 2—source data 1. [file elife-70541-fig4-figsupp2-data1.zip › Figure 4-figure supplment 2 Source data files/Figure 4-figure supplement 2-Source data (raw data)/Figure 4-figure supplement 2-Source data 4 (B_raw data).jpg]

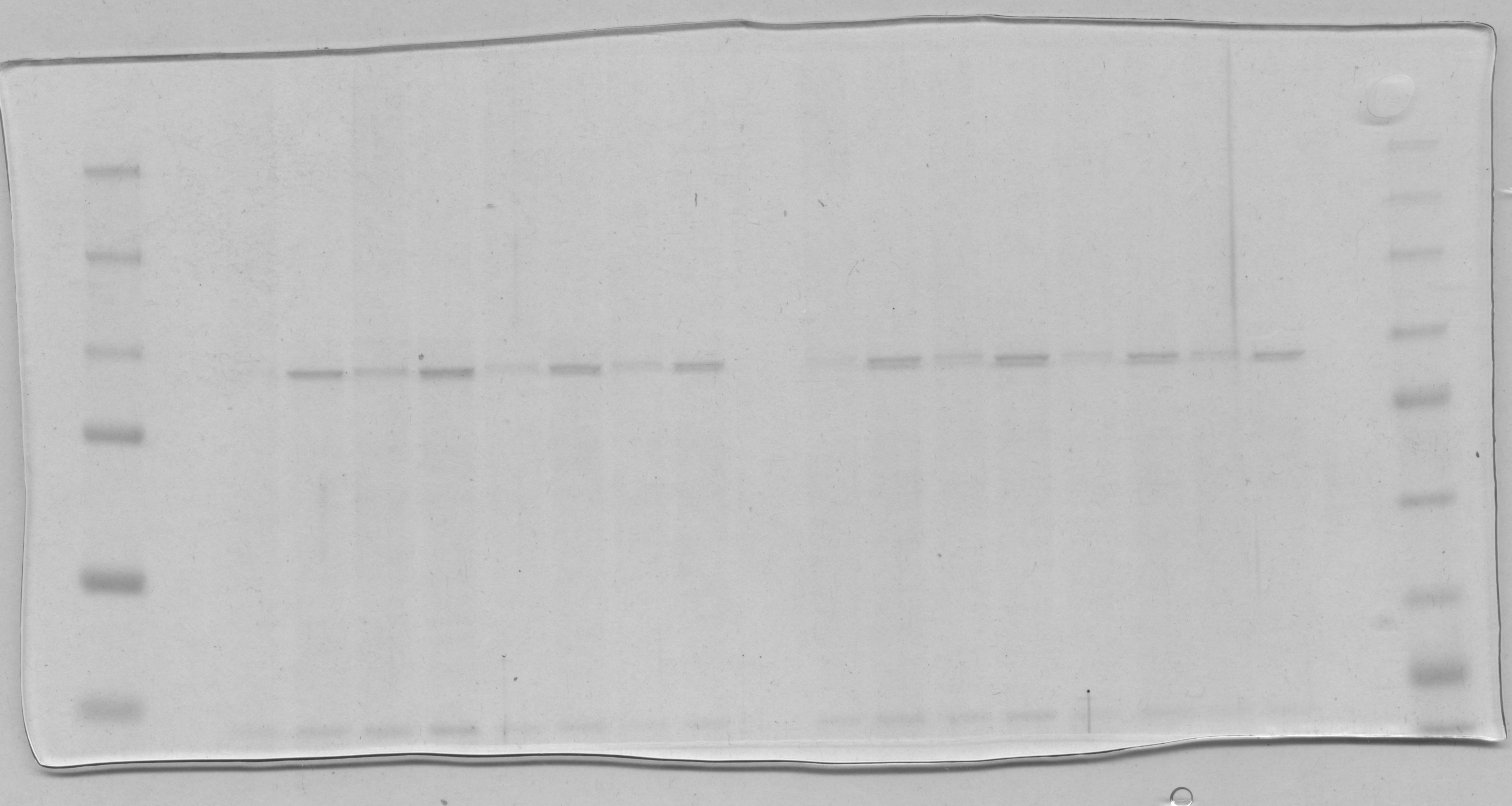

Supplement: Figure 4—figure supplement 3—source data 1. [file elife-70541-fig4-figsupp3-data1.zip › Figure 4-figure supplment 3 Source data files/Figure 4-figure supplement 3-Source data (raw data)/Figure 4-figure supplement 3-Source data 2 (A_raw data).jpg]

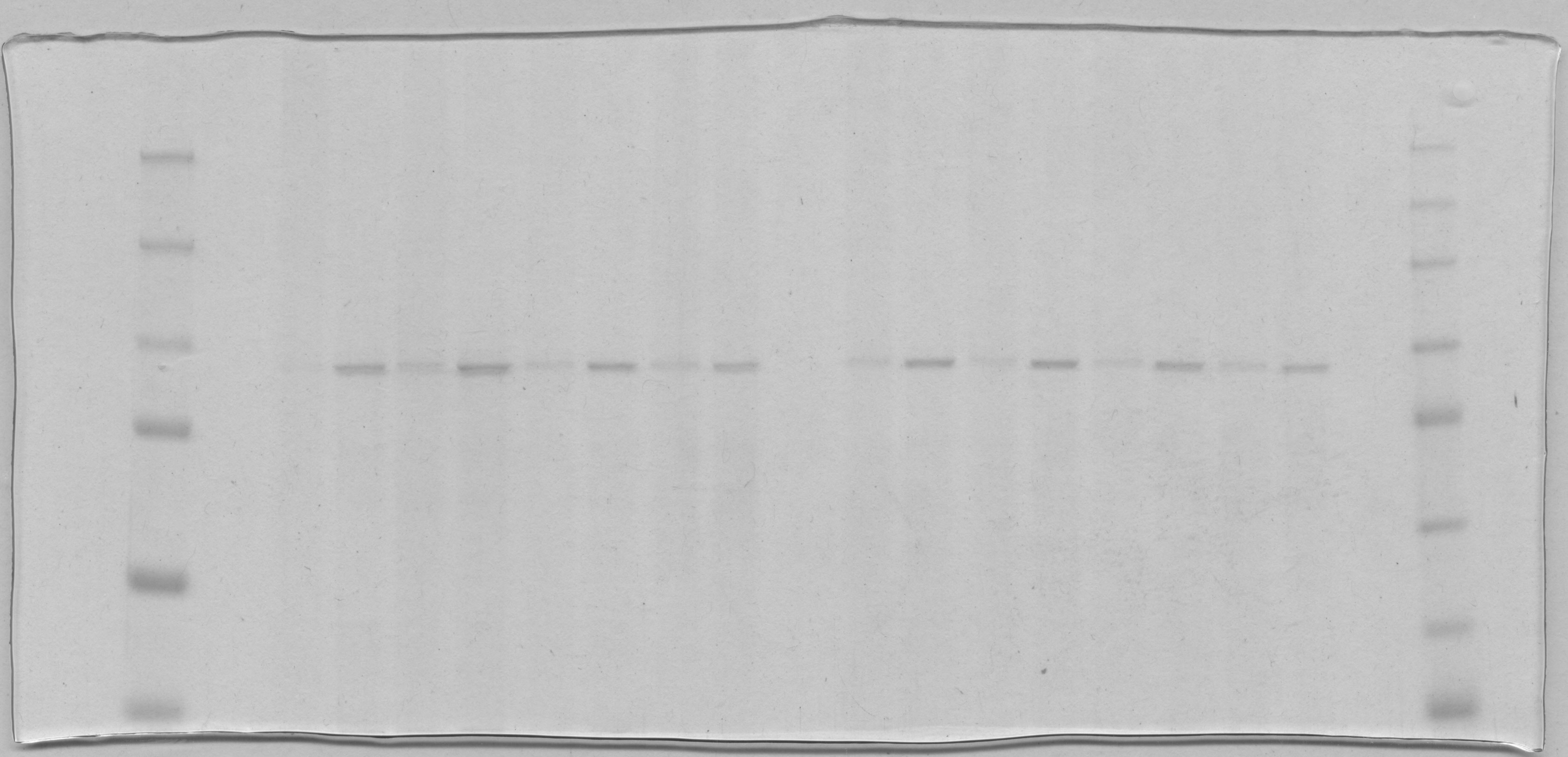

Supplement: Figure 4—figure supplement 3—source data 1. [file elife-70541-fig4-figsupp3-data1.zip › Figure 4-figure supplment 3 Source data files/Figure 4-figure supplement 3-Source data (raw data)/Figure 4-figure supplement 3-Source data 3 (B_raw data).jpg]

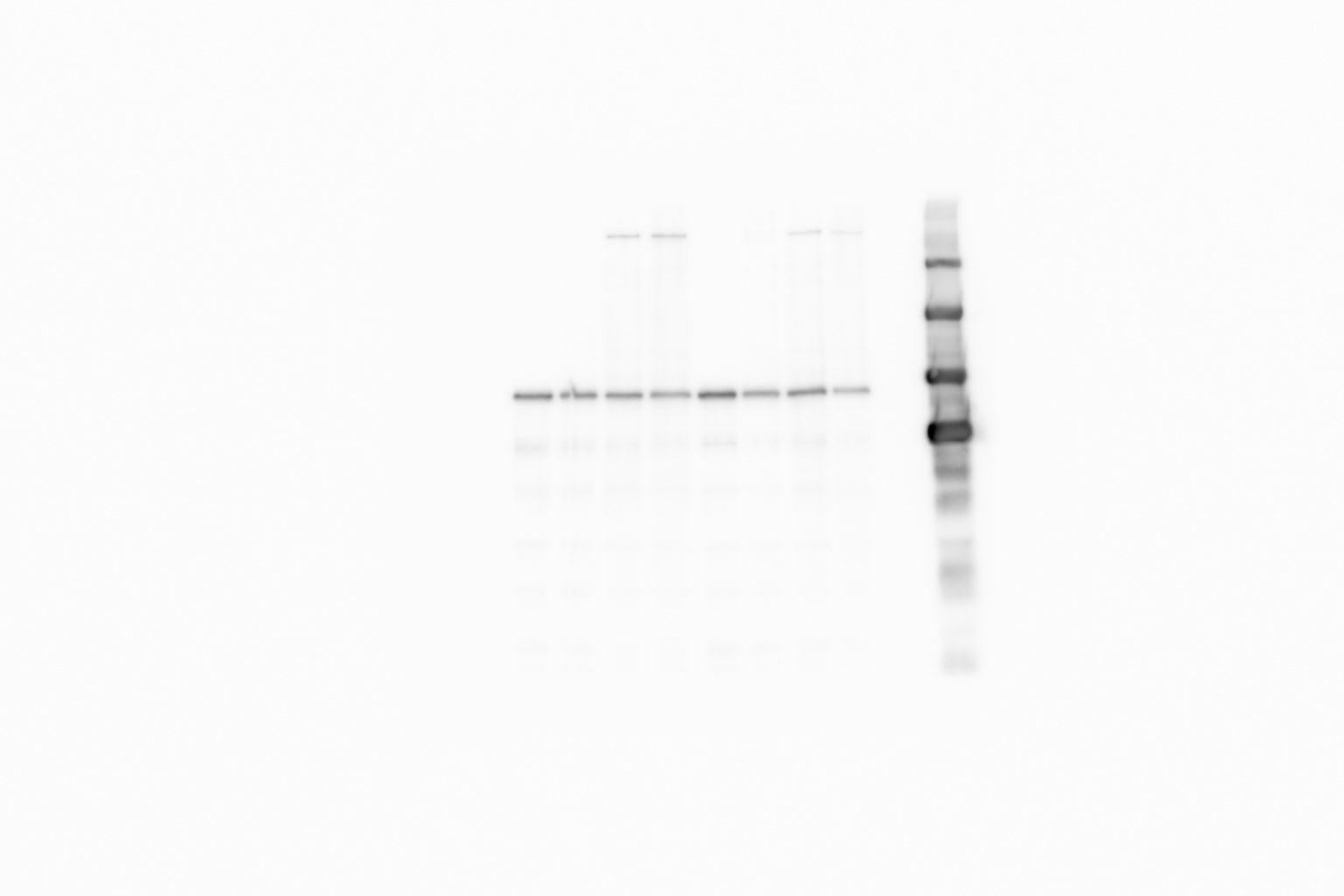

Supplement: Figure 4—figure supplement 3—source data 1. [file elife-70541-fig4-figsupp3-data1.zip › Figure 4-figure supplment 3 Source data files/Figure 4-figure supplement 3-Source data (raw data)/Figure 4-figure supplement 3-Source data 4 (C_raw data-His).tif]
