# Supplementary material for: Edge-strand of BepA interacts with immature LptD on the β-barrel assembly machine to direct it to on- and off-pathways
Source: eLife. 2021 Aug 31;10:e70541. doi: 10.7554/eLife.70541 (PMC8423444; doi:10.7554/eLife.70541)
Supplement: Figure 2—source data 1. [file elife-70541-fig2-data1.zip › Figure 2 Source data files/Fig 2-Source data 1 (A, B,_used area).pdf]

Figure 2-Source Data

Figure 2A

Peri αHis Original image Used area

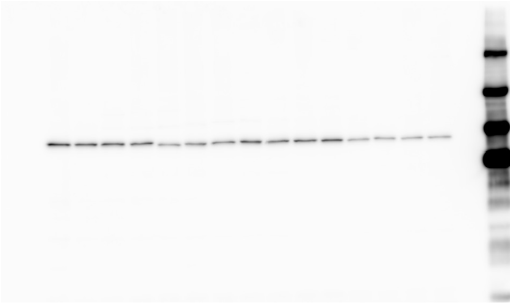

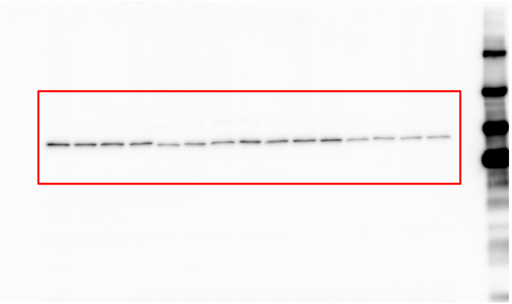

Peri αBepA Original image Used area

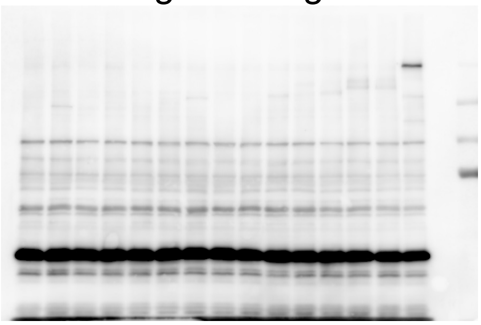

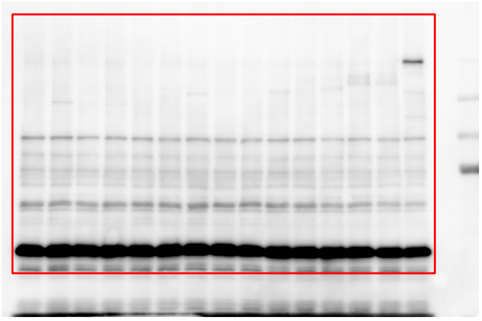

β-N αHis Original image Used area

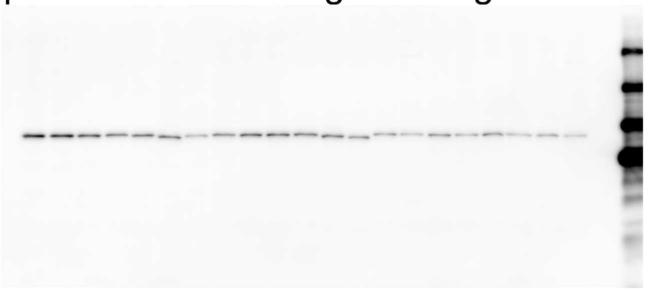

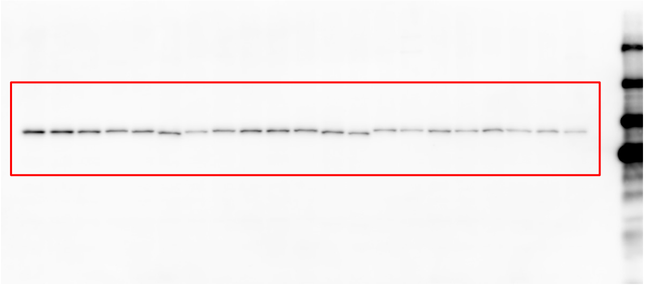

β-N αBepA Original image Used area

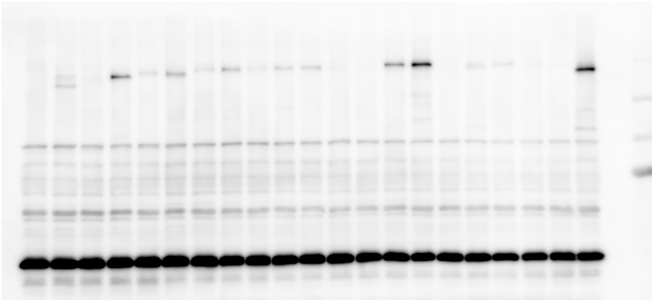

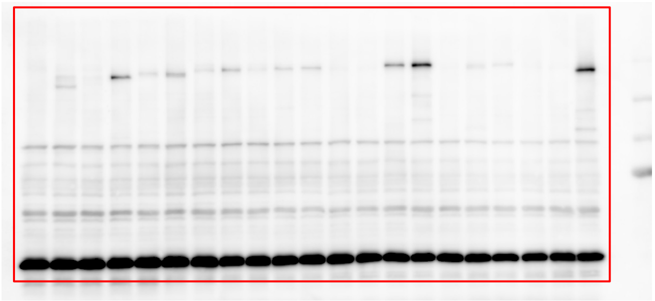

Figure 2-Source Data

β-C αHis Original image Used area

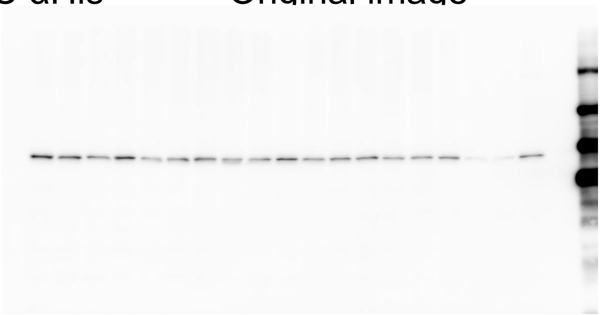

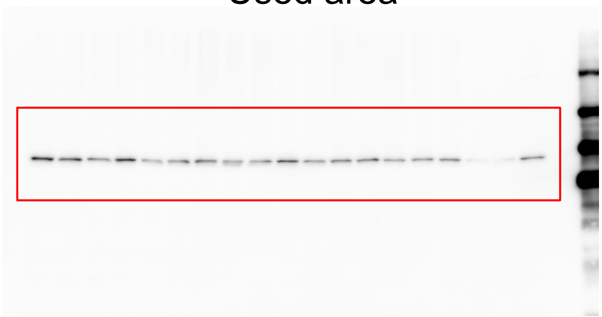

β-C αBepA Original image Used area

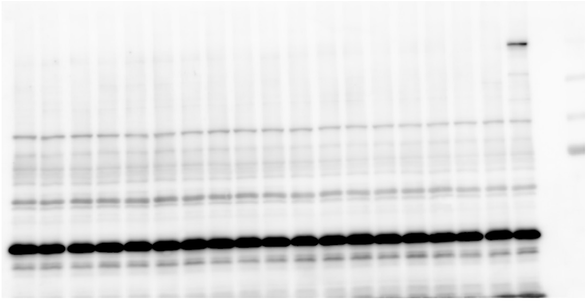

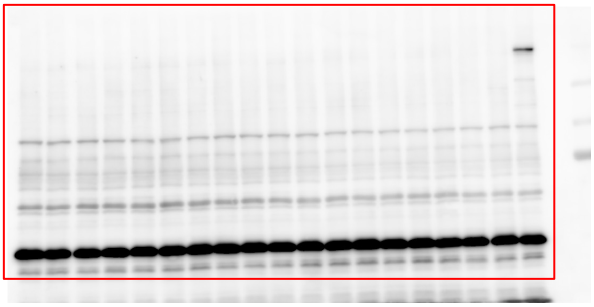

Figure 2B

αHis Original image Used area

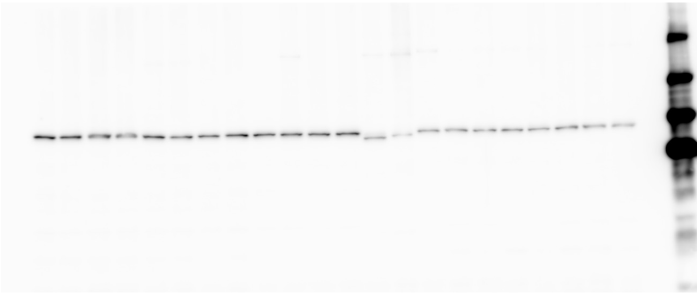

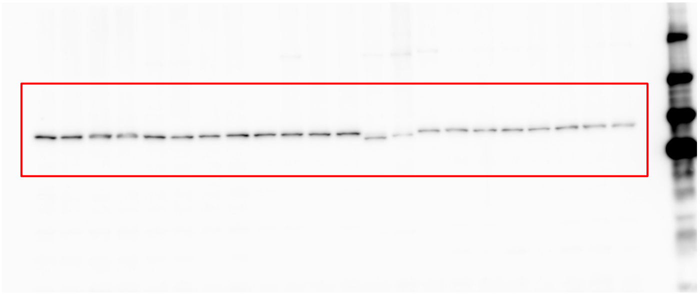

αBepA Original image Used area

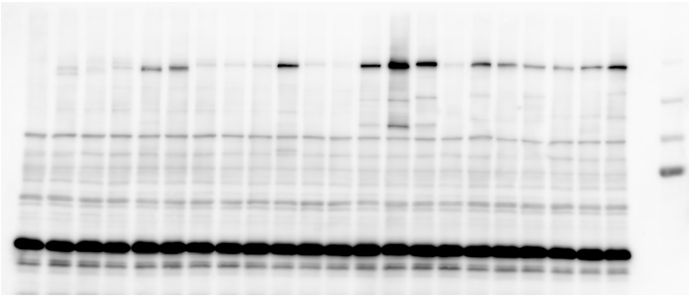

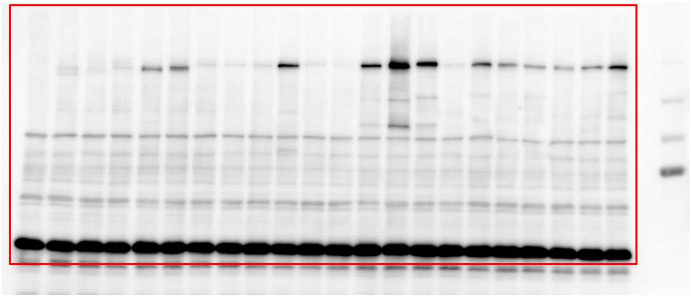

The "original" images were constructed by the image processing (including rotation, flip, contrast adjusting, and/or cropping) of the corresponding raw data.
