# Supplementary material for: Edge-strand of BepA interacts with immature LptD on the β-barrel assembly machine to direct it to on- and off-pathways
Source: eLife. 2021 Aug 31;10:e70541. doi: 10.7554/eLife.70541 (PMC8423444; doi:10.7554/eLife.70541)
Supplement: Figure 2—figure supplement 1—source data 1. [file elife-70541-fig2-figsupp1-data1.zip › Figure 2-figure supplment 1 Source data files/Figure 2-figure supplement 1-Source data 1 (used area).pdf.pdf]

Figure 2-figure supplement 1-Source Data

αLptD Original image

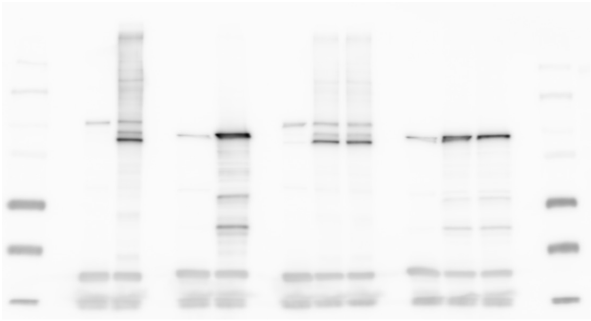

Used area

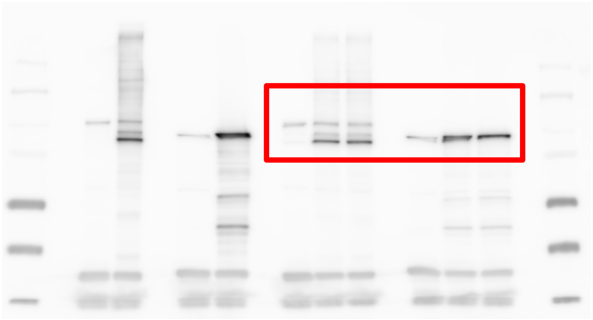

αHis Original image

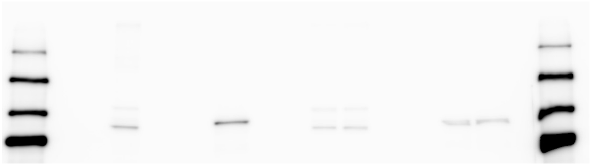

Used area

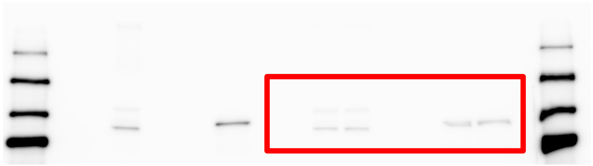

αBepA Original image

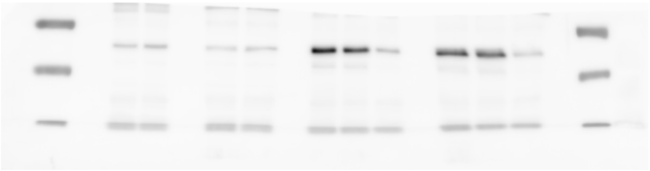

Used area

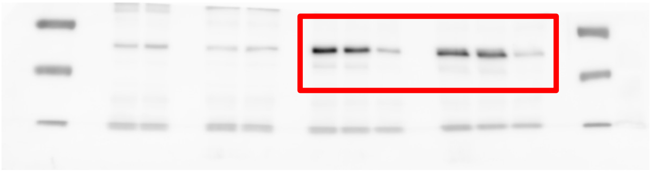

The "original" images were constructed by the image processing (including rotation, flip, contrast adjusting, and/or cropping) of the corresponding raw data.
