# Supplementary material for: Edge-strand of BepA interacts with immature LptD on the β-barrel assembly machine to direct it to on- and off-pathways
Source: eLife. 2021 Aug 31;10:e70541. doi: 10.7554/eLife.70541 (PMC8423444; doi:10.7554/eLife.70541)
Supplement: Figure 3—figure supplement 1—source data 1. [file elife-70541-fig3-figsupp1-data1.zip › Figure 3-figure supplment 1 Source data files/Figure 3-figure supplement 1-Source data 1 (used area).pdf.pdf]

Figure 3-figure supplement 1-Source Data

αBepA pure A106/F107

Original image

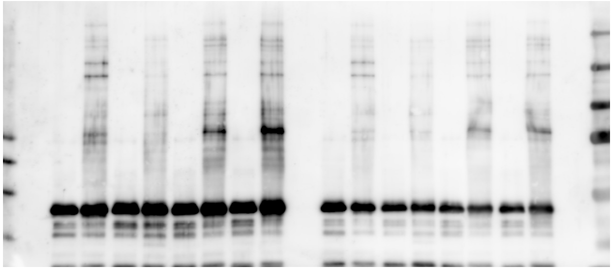

Used area

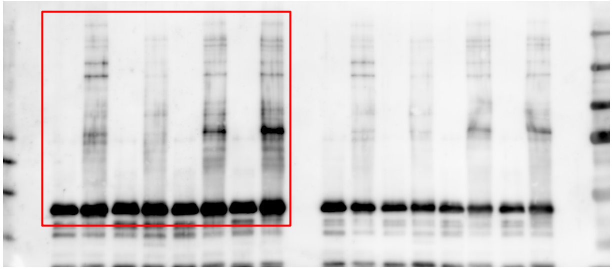

αLptD pure A106/F107

Original image

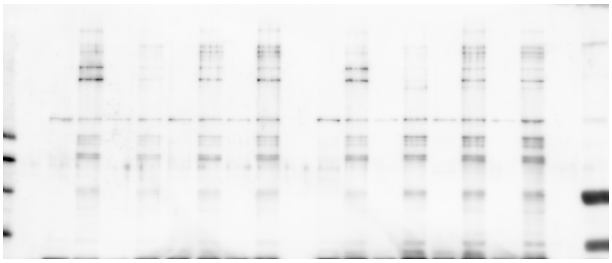

Used area

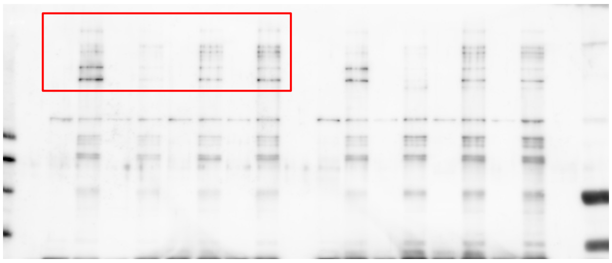

αBepA pure N105

Original image

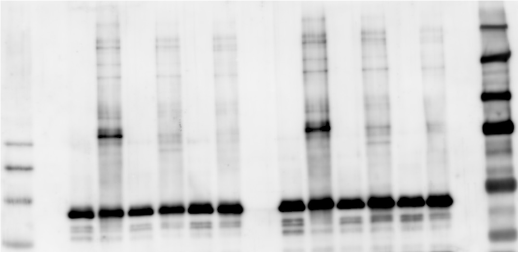

Used area

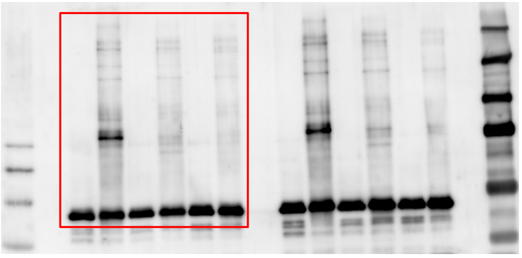

αLptD pure N105

Original image

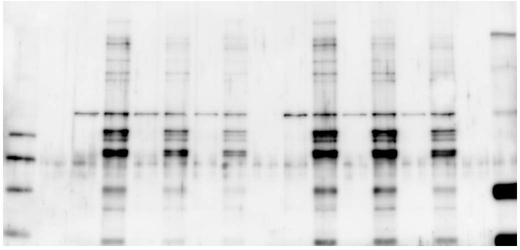

Used area

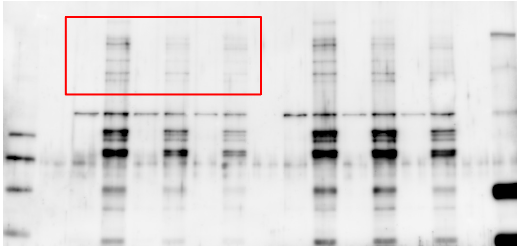

Figure 3-figure supplement 1-Source Data

αBepA$^{\text{Whole A106/F107}}$

Original image

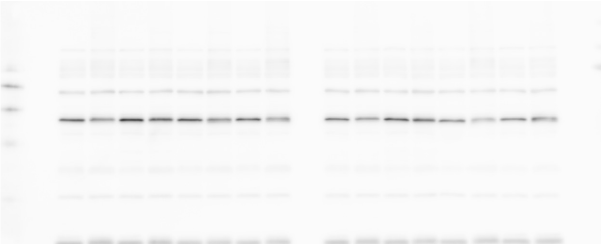

Used area

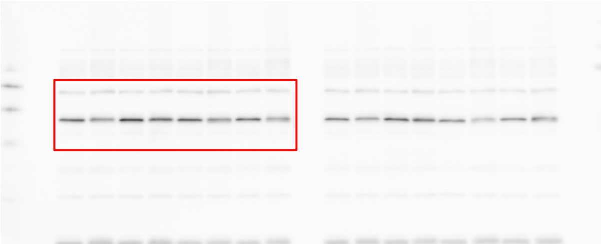

αBepA$^{\text{Whole N105}}$

Original image

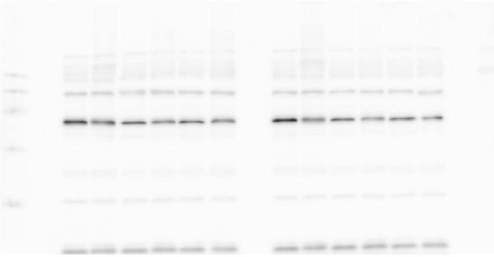

Used area

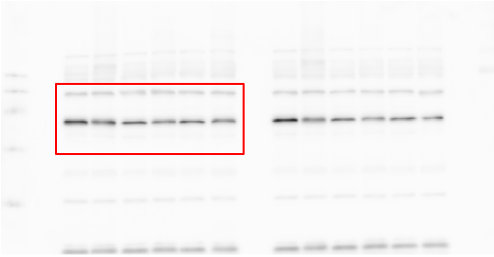

The "original" images were constructed by the image processing (including rotation, flip, contrast adjusting, and/or cropping) of the corresponding raw data.
