# Supplementary material for: Edge-strand of BepA interacts with immature LptD on the β-barrel assembly machine to direct it to on- and off-pathways
Source: eLife. 2021 Aug 31;10:e70541. doi: 10.7554/eLife.70541 (PMC8423444; doi:10.7554/eLife.70541)
Supplement: Figure 4—figure supplement 3—source data 1. [file elife-70541-fig4-figsupp3-data1.zip › Figure 4-figure supplment 3 Source data files/Figure 4-figure supplement 3-Source data 1 (A, B, C_used area).pdf.pdf]

Figure 4-figure supplement 3-Source Data

A

Original image

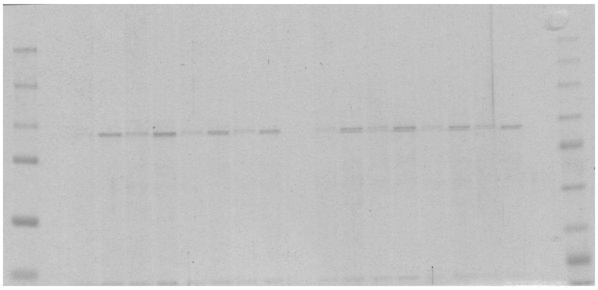

Used area

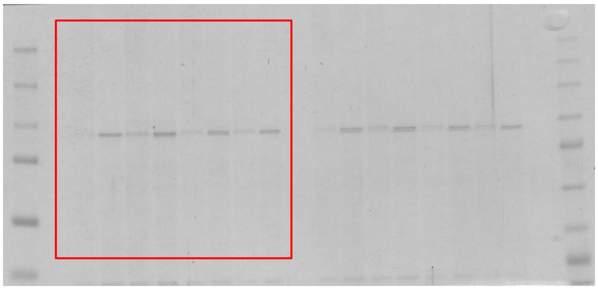

B

Original image

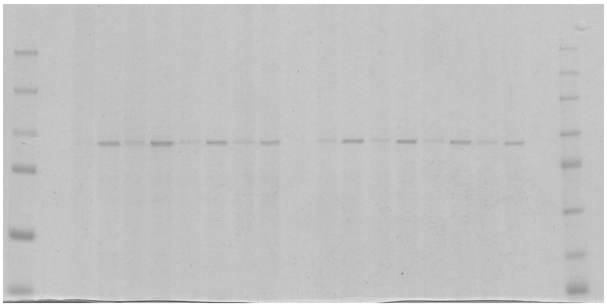

Used area

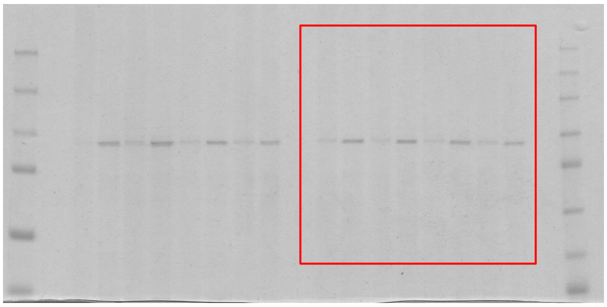

C

Original image

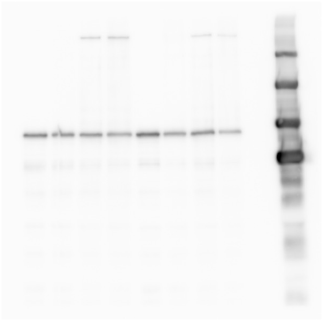

Used area

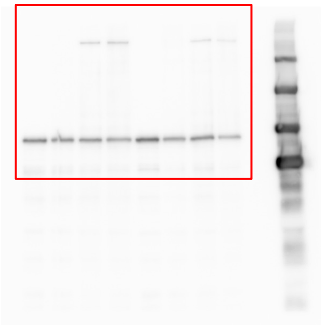

The "original" images were constructed by the image processing (including rotation, flip, contrast adjusting, and/or cropping) of the corresponding raw data.
