# Supplementary material for: Edge-strand of BepA interacts with immature LptD on the β-barrel assembly machine to direct it to on- and off-pathways
Source: eLife. 2021 Aug 31;10:e70541. doi: 10.7554/eLife.70541 (PMC8423444; doi:10.7554/eLife.70541)
Supplement: Supplementary file 1. [file elife-70541-supp1.docx]

**Table S1. Strains used in this study**

| Strain | Genotype | References |
| --- | --- | --- |
| AD16 | Δ*pro-lac* *thi*/F' *lacI^q^* *Z*Δ*M15* *Y*^+^ *pro*^+^ | (Kihara et al., 1995) |
| SN56 | AD16, Δ*bepA* | (Narita et al., 2013) |
| SN259 | AD16, Δ*bepA ΔsurA*::*kan* | (Narita et al., 2013) |
| RM2243 | AD16, *bepA(E137Q)* *purC80*::Tn*10* | This study |
| RM3654 | AD16, Δ*bepA* *bamA*^+^ *zae502*::Tn*10* | This study |
| RM3655 | AD16, Δ*bepA bamA(S439C) zae502*::Tn*10* | This study |
| JE6631 | *Hfr:str thi polA1* | (Mori et al., 2002) |
| RM2091 | JE6631, *purC80*::Tn*10* | This study |
| YH188 | JE6631, *zae502*::Tn*10* | (Hizukuri and Akiyama, 2012) |
| MC4100 | F^-^ *araD139* Δ*(argF-lac)U169 rpsL150 relA1 flbB5301 deoC1 ptsF25 rbsR* | (Casadaban, 1976) |
| CU141 | MC4100/F' *lacI^q^* *lacZ*^+^, *Y*^+^, *A*^+^ | (Akiyama et al., 1994) |
| HM1742 | CU141 *ara*^+^ | (Mori and Ito, 2006) |
| RM3588 | HM1742, *kan araC*-P*_araBAD_*-*lptD* | This study |
| DY330 | W3110, Δ*lacU169* *gal490* λ*cI857* Δ(*cro-bioA*) | (Yu et al., 2000) |
| RM3444 | DY330, *kan araC*-P*_araBAD_*-*lptD* | This study |

**(References)**

Akiyama Y, Ogura T, Ito K. 1994. Involvement of FtsH in protein assembly into and through the membrane. I. Mutations that reduce retention efficiency of a cytoplasmic reporter. *J Biol Chem* **269**:5218–5224. doi:10.1016/S0021-9258(17)37677-9

Casadaban MJ. 1976. Transposition and fusion of the *lac* genes to selected promoters in *Escherichia coli* using bacteriophage lambda and Mu. *J Mol Biol* **104**:541–555. doi:10.1016/0022-2836(76)90119-4

Hizukuri Y, Akiyama Y. 2012. PDZ domains of RseP are not essential for sequential cleavage of RseA or stress-induced σ^E^ activation *in vivo*. *Mol Microbiol* **86**:1232–1245. doi:10.1111/mmi.12053

Kihara A, Akiyama Y, Ito K. 1995. FtsH is required for proteolytic elimination of uncomplexed forms of SecY, an essential protein translocase subunit. *Proc Natl Acad Sci U S A* **92**:4532–4536. doi:10.1073/pnas.92.10.4532

Mori H, Ito K. 2006. The long α-helix of SecA is important for the ATPase coupling of translocation. *J Biol Chem* **281**:36249–36256. doi:10.1074/jbc.M606906200

Mori H, Shimizu Y, Ito K. 2002. Superactive SecY variants that fulfill the essential translocation function with a reduced cellular quantity. *J Biol Chem* **277**:48550–48557. doi:10.1074/jbc.M204436200

Narita S, Masui C, Suzuki T, Dohmae N, Akiyama Y. 2013. Protease homolog BepA (YfgC) promotes assembly and degradation of β-barrel membrane proteins in *Escherichia coli*. *Proc Natl Acad Sci U S A* **110**:E3612–E3621. doi:10.1073/pnas.1312012110

Yu D, Ellis HM, Lee EC, Jenkins NA, Copeland NG, Court DL. 2000. An efficient recombination system for chromosome engineering in *Escherichia coli.* *Proc Natl Acad Sci U S A* **97**:5978–5983. doi:10.1073/pnas.100127597
