# Supplementary material for: Edge-strand of BepA interacts with immature LptD on the β-barrel assembly machine to direct it to on- and off-pathways
Source: eLife. 2021 Aug 31;10:e70541. doi: 10.7554/eLife.70541 (PMC8423444; doi:10.7554/eLife.70541)
Supplement: Supplementary file 2. [file elife-70541-supp2.docx]

**Table S2. Plasmids used in this study**

| Plasmid | Vector | Encoded protein and description | Reference or source |
| --- | --- | --- | --- |
| pEVOL-pBpF |  | p15A-derivative encoding an evolved *M. jannaschii* aminoacyl-tRNA synthetase/ suppressor tRNA pair for incorporation of *p*BPA; CmR | (Young et al., 2010) |
| pSTD689 |  | pACYC184-derived expression vector; P*_lac_*, Spc^R^ | (Kanehara et al., 2003) |
| pRM290 | pSTD689 | BepA | (Daimon et al., 2017) |
| pRM291 | pSTD689 | BepA(E137Q) | (Daimon et al., 2017) |
| pRM512 | pSTD689 | BepA(N105P) | This study |
| pRM513 | pSTD689 | BepA(A106P) | This study |
| pRM514 | pSTD689 | BepA(F107P) | This study |
| pRM515 | pSTD689 | BepA(A108P) | This study |
| pRM516 | pSTD689 | BepA(F109P) | This study |
| pRM517 | pSTD689 | BepA(F110P) | This study |
| pRM833 | pSTD689 | BepA(N105C) | This study |
| pRM786 | pSTD689 | BepA(N105C, E137Q) | This study |
| pRM834 | pSTD689 | BepA(A106C) | This study |
| pRM787 | pSTD689 | BepA(A106C, E137Q) | This study |
| pRM835 | pSTD689 | BepA(F107C) | This study |
| pRM788 | pSTD689 | BepA(F107C, E137Q) | This study |
| pUC18 |  | Expression vector; P*_lac_*, Amp^R^ | Takara Bio |
| pUC-bepA(E137Q) | pUC18 | BepA(E137Q) | (Narita et al., 2013) |
| pUC-bepA-his_10_ | pUC18 | BepA-His_10_ | (Narita et al., 2013) |
| pUC-bepA(E137Q)-his_10_ | pUC18 | BepA(E137Q)-His_10_ | (Narita et al., 2013) |
| pYN333 | pUC18 | BepA(N105P)-His_10_ | This study |
| pYN334 | pUC18 | BepA(A106P)-His_10_ | This study |
| pYN335 | pUC18 | BepA(F107P)-His_10_ | This study |
| pYN336 | pUC18 | BepA(A108P)-His_10_ | This study |
| pYN337 | pUC18 | BepA(F109P)-His_10_ | This study |
| pYN338 | pUC18 | BepA(F110P)-His_10_ | This study |
| pYN339 | pUC18 | BepA(N105*amb*)-His_10_ | This study |
| pRM536 | pUC18 | BepA(N105*amb*, E137Q)-His_10_ | This study |
| pRM778 | pUC18 | BepA(N105*amb*, A106P, E137Q)-His_10_ | This study |
| pRM779 | pUC18 | BepA(N105*amb*, F107P, E137Q)-His_10_ | This study |
| pYN340 | pUC18 | BepA(A106*amb*)-His_10_ | This study |
| pRM537 | pUC18 | BepA(A106*amb*, E137Q)-His_10_ | This study |
| pYN345 | pUC18 | BepA(A106*amb*, F107P, E137Q)-His_10_ | This study |
| pYN341 | pUC18 | BepA(F107*amb*)-His_10_ | This study |
| pRM538 | pUC18 | BepA(F107*amb*, E137Q)-His_10_ | This study |
| pYN346 | pUC18 | BepA(F107*amb*, A106P, E137Q)-His_10_ | This study |
| pYN342 | pUC18 | BepA(A108*amb*)-His_10_ | This study |
| pRM539 | pUC18 | BepA(A108*amb*, E137Q)-His_10_ | This study |
| pYN343 | pUC18 | BepA(F109*amb*)-His_10_ | This study |
| pRM540 | pUC18 | BepA(F109*amb*, E137Q)-His_10_ | This study |
| pYN344 | pUC18 | BepA(F110*amb*)-His_10_ | This study |
| pRM541 | pUC18 | BepA(F110*amb*, E137Q)-His_10_ | This study |
| pMW118 |  | pSC101-derived expression vector; P*_lac_*, Amp^R^ | Nippon Gene |
| pNB91 | pMW118 | BepA(E137Q) | This study |
| pRM807 | pMW118 | BepA(A106P, E137Q) | This study |
| pRM808 | pMW118 | BepA(F107P, E137Q) | This study |
| pTWV228 |  | pBR322-derived expression vector; P*_lac_*, Amp^R^ | Takara Bio |
| pRM267 | pTWV228 | LptD-His_10_ (using the native SD sequence) | (Daimon et al., 2017) |
| pRM816 | pTWV228 | LptD-His_10_^SD^ (using a strong SD sequence) | This study |
| pRM810 | pTWV228 | LptD(M287C)-His_10_^SD^ | This study |
| pRM811 | pTWV228 | LptD(Y331C)-His_10_^SD^ | This study |
| pRM812 | pTWV228 | LptD(E391C)-His_10_^SD^ | This study |
| pRM813 | pTWV228 | LptD(Q393C)-His_10_^SD^ | This study |
| pRM814 | pTWV228 | LptD(H416C)-His_10_^SD^ | This study |
| pRM815 | pTWV228 | LptD(V430C)-His_10_^SD^ | This study |
| pRM294 |  | pBR322-derived expression vector; P*_lac_*, Spc^R^ | (Miyazaki et al., 2018) |
| pRM309 | pRM294 | LptD-His_10_^SD^ (using a strong SD sequence) | (Miyazaki et al., 2018) |
| pNB22 | pRM294 | LptD(R40*amb*)-His_10_^SD^ | This study |
| pNB23 | pRM294 | LptD(N55*amb*)-His_10_^SD^ | This study |
| pNB24 | pRM294 | LptD(T70*amb*)-His_10_^SD^ | This study |
| pNB25 | pRM294 | LptD(D85*amb*)-His_10_^SD^ | This study |
| pNB26 | pRM294 | LptD(P100*amb*)-His_10_^SD^ | This study |
| pNB27 | pRM294 | LptD(N115*amb*)-His_10_^SD^ | This study |
| pNB28 | pRM294 | LptD(T130*amb*)-His_10_^SD^ | This study |
| pNB29 | pRM294 | LptD(R145*amb*)-His_10_^SD^ | This study |
| pNB30 | pRM294 | LptD(N160*amb*)-His_10_^SD^ | This study |
| pNB31 | pRM294 | LptD(P175*amb*)-His_10_^SD^ | This study |
| pNB49 | pRM294 | LptD(D190*amb*)-His_10_^SD^ | This study |
| pNB50 | pRM294 | LptD(V205*amb*)-His_10_^SD^ | This study |
| pNB51 | pRM294 | LptD(V220*amb*)-His_10_^SD^ | This study |
| pNB52 | pRM294 | LptD(Y235*amb*)-His_10_^SD^ | This study |
| pNB53 | pRM294 | LptD(N250*amb*)-His_10_^SD^ | This study |
| pRM618 | pRM294 | LptD(T258*amb*)-His_10_^SD^ | This study |
| pRM619 | pRM294 | LptD(I259*amb*)-His_10_^SD^ | This study |
| pNB54 | pRM294 | LptD(H265*amb*)-His_10_^SD^ | This study |
| pRM620 | pRM294 | LptD(E273*amb*)-His_10_^SD^ | This study |
| pNB55 | pRM294 | LptD(S280*amb*)-His_10_^SD^ | This study |
| pRM621 | pRM294 | LptD(L286*amb*)-His_10_^SD^ | This study |
| pRM622 | pRM294 | LptD(M287*amb*)-His_10_^SD^ | This study |
| pNB56 | pRM294 | LptD(S295*amb*)-His_10_^SD^ | This study |
| pNB57 | pRM294 | LptD(R310*amb*)-His_10_^SD^ | This study |
| pRM623 | pRM294 | LptD(Y314*amb*)-His_10_^SD^ | This study |
| pNB58 | pRM294 | LptD(W325*amb*)-His_10_^SD^ | This study |
| pRM624 | pRM294 | LptD(F327*amb*)-His_10_^SD^ | This study |
| pRM625 | pRM294 | LptD(D330*amb*)-His_10_^SD^ | This study |
| pRM626 | pRM294 | LptD(Y331*amb*)-His_10_^SD^ | This study |
| pNB59 | pRM294 | LptD(F340*amb*)-His_10_^SD^ | This study |
| pNB60 | pRM294 | LptD(A355*amb*)-His_10_^SD^ | This study |
| pRM627 | pRM294 | LptD(K358*amb*)-His_10_^SD^ | This study |
| pNB61 | pRM294 | LptD(A370*amb*)-His_10_^SD^ | This study |
| pRM628 | pRM294 | LptD(T371*amb*)-His_10_^SD^ | This study |
| pNB62 | pRM294 | LptD(T385*amb*)-His_10_^SD^ | This study |
| pRM629 | pRM294 | LptD(E391*amb*)-His_10_^SD^ | This study |
| pRM630 | pRM294 | LptD(Q393*amb*)-His_10_^SD^ | This study |
| pNB63 | pRM294 | LptD(Q400*amb*)-His_10_^SD^ | This study |
| pNB64 | pRM294 | LptD(V415*amb*)-His_10_^SD^ | This study |
| pRM631 | pRM294 | LptD(H416*amb*)-His_10_^SD^ | This study |
| pRM632 | pRM294 | LptD(R429*amb*)-His_10_^SD^ | This study |
| pNB65 | pRM294 | LptD(V430*amb*)-His_10_^SD^ | This study |
| pRM633 | pRM294 | LptD(H431*amb*)-His_10_^SD^ | This study |
| pNB66 | pRM294 | LptD(G445*amb*)-His_10_^SD^ | This study |
| pRM634 | pRM294 | LptD(H457*amb*)-His_10_^SD^ | This study |
| pRM635 | pRM294 | LptD(Y458*amb*)-His_10_^SD^ | This study |
| pNB67 | pRM294 | LptD(Q460*amb*)-His_10_^SD^ | This study |
| pNB68 | pRM294 | LptD(D475*amb*)-His_10_^SD^ | This study |
| pRM636 | pRM294 | LptD(V478*amb*)-His_10_^SD^ | This study |
| pRM637 | pRM294 | LptD(N479*amb*)-His_10_^SD^ | This study |
| pNB69 | pRM294 | LptD(K490*amb*)-His_10_^SD^ | This study |
| pNB70 | pRM294 | LptD(T505*amb*)-His_10_^SD^ | This study |
| pNB71 | pRM294 | LptD(R520*amb*)-His_10_^SD^ | This study |
| pNB72 | pRM294 | LptD(S535*amb*)-His_10_^SD^ | This study |
| pNB73 | pRM294 | LptD(D550*amb*)-His_10_^SD^ | This study |
| pNB74 | pRM294 | LptD(R565*amb*)-His_10_^SD^ | This study |
| pNB75 | pRM294 | LptD(G580*amb*)-His_10_^SD^ | This study |
| pNB76 | pRM294 | LptD(I595*amb*)-His_10_^SD^ | This study |
| pNB77 | pRM294 | LptD(G610*amb*)-His_10_^SD^ | This study |
| pNB78 | pRM294 | LptD(G625*amb*)-His_10_^SD^ | This study |
| pNB79 | pRM294 | LptD(S640*amb*)-His_10_^SD^ | This study |
| pNB80 | pRM294 | LptD(N655*amb*)-His_10_^SD^ | This study |
| pNB81 | pRM294 | LptD(K670*amb*)-His_10_^SD^ | This study |
| pNB82 | pRM294 | LptD(V685*amb*)-His_10_^SD^ | This study |
| pNB83 | pRM294 | LptD(V700*amb*)-His_10_^SD^ | This study |
| pNB84 | pRM294 | LptD(S715*amb*)-His_10_^SD^ | This study |
| pNB32 | pRM294 | LptD(Y726*amb*)-His_10_^SD^ | This study |
| pNB85 | pRM294 | LptD(V730*amb*)-His_10_^SD^ | This study |
| pNB86 | pRM294 | LptD(H745*amb*)-His_10_^SD^ | This study |
| pRM809 | pRM294 | LptD(D749*amb*)-His_10_^SD^ | This study |
| pNB87 | pRM294 | LptD(G760*amb*)-His_10_^SD^ | This study |
| pNB88 | pRM294 | LptD(S775*amb*)-His_10_^SD^ | This study |
| pRM821 | pRM294 | LptD(Y331*amb*, D749*amb*)-His_10_^SD^ | This study |
| pRM822 | pRM294 | LptD(Y331*amb*, Y726*amb*)-His_10_^SD^ | This study |
| pRM829 | pRM294 | LptD(E733C)-His_10_^SD^ | This study |
| pRM831 | pRM294 | LptD(Y331*amb*, E733C)-His_10_^SD^ | This study |
| pUC118 |  | Expression vector; P*_lac_*, Amp^R^ | Takara Bio |
| pRM320 | pUC118 | *bepA-yfgD* | This study |
| pRM324 | pUC118 | *bepA(E137Q)-yfgD* | This study |
| pRM823 | pUC118 | *bamA* | This study |
| pRM836 | pUC118 | *bamA(S439C)* | This study |
| pK18mobsacB |  | pBR322-derived vector carrying *sacB* for chromosomal gene replacement; Km^R^ | (Schäfer et al., 1994) |
| pRM330 | pK18mobsacB | *bepA(E137Q)-yfgD* | This study |
| pRM845 | pK18mobsacB | *bamA(S439C)* | This study |

**(References)**

Daimon Y, Iwama-Masui C, Tanaka Y, Shiota T, Suzuki T, Miyazaki R, Sakurada H, Lithgow T, Dohmae N, Mori H, Tsukazaki T, Narita S-I, Akiyama Y. 2017. The TPR domain of BepA is required for productive interaction with substrate proteins and the β-barrel assembly machinery complex. *Mol Microbiol* **106**:760–776. doi:10.1111/mmi.13844

Kanehara K, Ito K, Akiyama Y. 2003. YaeL proteolysis of RseA is controlled by the PDZ domain of YaeL and a Gln-rich region of RseA. *EMBO J* **22**:6389–6398. doi:10.1093/emboj/cdg602

Miyazaki R, Myougo N, Mori H, Akiyama Y. 2018. A photo-cross-linking approach to monitor folding and assembly of newly synthesized proteins in a living cell. *J Biol Chem* **293**:677–686. doi:10.1074/jbc.M117.817270

Narita S, Masui C, Suzuki T, Dohmae N, Akiyama Y. 2013. Protease homolog BepA (YfgC) promotes assembly and degradation of β-barrel membrane proteins in *Escherichia coli*. *Proc Natl Acad Sci U S A* **110**:E3612–E3621. doi:10.1073/pnas.1312012110

Schäfer A, Tauch A, Jäger W, Kalinowski J, Thierbach G, Pühler A. 1994. Small mobilizable multi-purpose cloning vectors derived from the *Escherichia coli* plasmids pK18 and pK19: selection of defined deletions in the chromosome of *Corynebacterium glutamicum*. *Gene* **145**:69–73. doi:10.1016/0378-1119(94)90324-7

Young TS, Ahmad I, Yin JA, Schultz PG. 2010. An enhanced system for unnatural amino acid mutagenesis in *E. coli*. *J Mol Biol* **395**:361–374. doi:10.1016/j.jmb.2009.10.030
