# Supplementary material for: Edge-strand of BepA interacts with immature LptD on the β-barrel assembly machine to direct it to on- and off-pathways
Source: eLife. 2021 Aug 31;10:e70541. doi: 10.7554/eLife.70541 (PMC8423444; doi:10.7554/eLife.70541)
Supplement: Transparent reporting form [file elife-70541-transrepform.docx]

***eLife’s* transparent reporting form**

We encourage authors to provide detailed information *within their submission* to facilitate the interpretation and replication of experiments. Authors can upload supporting documentation to indicate the use of appropriate reporting guidelines for health-related research (see [EQUATOR Network](http://www.equator-network.org/%20)), life science research (see the [BioSharing Information Resource](https://biosharing.org/)), or the [ARRIVE guidelines](http://www.plosbiology.org/article/info:doi/10.1371/journal.pbio.1000412) for reporting work involving animal research. Where applicable, authors should refer to any relevant reporting standards documents in this form.

If you have any questions, please consult our Journal Policies and/or contact us: [editorial@elifesciences.org](mailto:editorial@elifesciences.org).

**Sample-size estimation**

- You should state whether an appropriate sample size was computed when the study was being designed
- You should state the statistical method of sample size computation and any required assumptions
- If no explicit power analysis was used, you should describe how you decided what sample (replicate) size (number) to use

Please outline where this information can be found within the submission (e.g., sections or figure legends), or explain why this information doesn’t apply to your submission:

We repeated experiments multiple times (see below) and present a representative data in the manuscript. Thus, the information on sample-size estimation does not apply to our submission.

**Replicates**

- You should report how often each experiment was performed
- You should include a definition of biological versus technical replication
- The data obtained should be provided and sufficient information should be provided to indicate the number of independent biological and/or technical replicates
- If you encountered any outliers, you should describe how these were handled
- Criteria for exclusion/inclusion of data should be clearly stated
- High-throughput sequence data should be uploaded before submission, with a private link for reviewers provided (these are available from both GEO and ArrayExpress)

Please outline where this information can be found within the submission (e.g., sections or figure legends), or explain why this information doesn’t apply to your submission:

We repeated experiments multiple times and found that the results are reproducible. Basically, we carried out technical replication experiments that were conducted using the same transformants. Information on the number of technical replicates for each experiment are included in the legends to Figure 1, 2, 3 and 4 and the legends to Figure1-figure supplement 2, 3 and 4, Figure 2-figure supplement 2 and Figure 3- figure supplement 1 and 2, Figure 4- figure supplement 1, 2 and 3.

In Figure 2-figure supplement 1, we carried out biological replication experiments that were conducted using the different transformants. Information on the number of biological replicates for this experiment is included in its legends.

**Statistical reporting**

- Statistical analysis methods should be described and justified
- Raw data should be presented in figures whenever informative to do so (typically when N per group is less than 10)
- For each experiment, you should identify the statistical tests used, exact values of N, definitions of center, methods of multiple test correction, and dispersion and precision measures (e.g., mean, median, SD, SEM, confidence intervals; and, for the major substantive results, a measure of effect size (e.g., Pearson's r, Cohen's d)
- Report exact p-values wherever possible alongside the summary statistics and 95% confidence intervals. These should be reported for all key questions and not only when the p-value is less than 0.05.

Please outline where this information can be found within the submission (e.g., sections or figure legends), or explain why this information doesn’t apply to your submission:

We repeated experiments multiple times (see above) and presented a representative data. In several experiments, we conducted statistical analysis using the Microsoft Excel software. We defined that center value and error bar in graphs represent mean value and S. D., respectively. Information on exact values of N and the statistical definition is presented in the legends to Figure 1D.

(For large datasets, or papers with a very large number of statistical tests, you may upload a single table file with tests, Ns, etc., with reference to sections in the manuscript.)

**Group allocation**

- Indicate how samples were allocated into experimental groups (in the case of clinical studies, please specify allocation to treatment method); if randomization was used, please also state if restricted randomization was applied
- Indicate if masking was used during group allocation, data collection and/or data analysis

Please outline where this information can be found within the submission (e.g., sections or figure legends), or explain why this information doesn’t apply to your submission:

Our research does not target animals and a human being. Thus, the information on group allocation does not apply to our submission.

**Additional data files (“source data”)**

- We encourage you to upload relevant additional data files, such as numerical data that are represented as a graph in a figure, or as a summary table
- Where provided, these should be in the most useful format, and they can be uploaded as “Source data” files linked to a main figure or table
- Include model definition files including the full list of parameters used
- Include code used for data analysis (e.g., R, MatLab)
- Avoid stating that data files are “available upon request”

Please indicate the figures or tables for which source data files have been provided:

Source data files have been provided for Figures 1, 2, 3, and 4, Figure 1-figure supplements 2, 3, and 4, Figure 2-figure supplements 1, and 2, Figure 3-figure supplements 1, and 2, and Figure 4-figure supplements 1, 2, and 3.
